# Phylogenetics of Lepidonotopodini (Macellicephalinae, Polynoidae, Annelida) and Comparative Mitogenomics of Shallow-Water vs. Deep-Sea Scaleworms (Aphroditiformia) †

**DOI:** 10.3390/biology13120979

**Published:** 2024-11-27

**Authors:** Avery S. Hiley, Nicolás Mongiardino Koch, Greg W. Rouse

**Affiliations:** Scripps Institution of Oceanography, University of California San Diego, La Jolla, CA 92093-0202, USA; nmongiardinokoch@ucsd.edu

**Keywords:** chemosynthetic-based ecosystems, deep sea, gene order rearrangement, Lepidonotopodini, Macellicephalinae, mitogenomes, molecular phylogeny, positive selection, scaleworms, taxonomic revisions

## Abstract

Within Polynoidae, a diverse aphroditiform family, the subfamily Macellicephalinae comprises anchialine cave-dwelling and deep-sea scaleworms. In this study, Lepidonotopodinae is synonymized with Macellicephalinae, and the tribe Lepidonotopodini is applied to a well-supported clade inhabiting deep-sea chemosynthetic-based ecosystems. Newly sequenced “genome skimming” data for 30 deep-sea polynoids and the comparatively shallow living *Eulagisca gigantea* is used to bioinformatically assemble their mitogenomes. When analyzed with existing scaleworm mitogenomes, deep-sea scaleworms exhibit increased gene order rearrangement events compared to shallow-water relatives. Additionally, comparative analyses of shallow-water vs. deep-sea polynoid substitution rates in mitochondrial protein-coding genes show an overall relaxed purifying selection and a positive selection of several amino acid sites in deep-sea species, indicating that polynoid mitogenomes have undergone selective pressure to evolve metabolic adaptations suited to deep-sea environments. Furthermore, the inclusion of skimming data for already known Lepidonotopodini species allowed for an increased coverage of DNA data and a representation of the taxa necessary to create a more robust phylogeny using 18 genes, as opposed to the six genes previously used. The phylogenetic results support the erection of *Cladopolynoe* gen. nov., *Mamiwata* gen. nov., *Photinopolynoe* gen. nov., *Stratigos* gen. nov., and *Themis* gen. nov., and emended diagnoses for *Branchinotogluma, Branchipolynoe*, *Lepidonotopodium*, and *Levensteiniella*.

## Simple Summary

Within Polynoidae, a diverse scaleworm family, the subfamily Macellicephalinae comprises anchialine cave-dwelling and deep-sea scaleworms and is emended herein to include all genera previously placed in the subfamily Lepidonotopodinae. Additionally, the tribe Lepidonotopodini is applied to a well-supported clade that exclusively inhabits deep-sea chemosynthetic-based ecosystems. These and other taxonomic changes are based on the analysis of newly sequenced “genome skimming” data for 30 deep-sea scaleworms and one comparatively shallow living relative, *Eulagisca gigantea*. The data were bioinformatically assembled into complete mitogenomes with high read coverage and resolution. When analyzed alongside pre-existing scaleworm mitogenomes, deep-sea scaleworms were found to exhibit increased gene order rearrangement events, a relaxed purifying selection of mitochondrial protein-coding genes, and a positive selection of amino acid sites compared to their shallow-water relatives. Furthermore, the inclusion of skimming data for already-known Lepidonotopodini species allowed for the assembly of a molecular dataset with increased coverage and taxon sampling. The phylogenies obtained support the erection of five new genera and the amendment of four pre-existing ones.

## 1. Introduction

Aphroditiformia Levinsen, 1883 is a clade of polychaete worms commonly referred to as scaleworms because they mostly possess elytra (scales) on their dorsal surface, which are hypothesized to be a protective adaptation. Polynoidae Kinberg, 1856 is a diverse family of scaleworms containing approximately 230 genera and over 1700 species from a wide range of marine habitats [1]. Within Polynoidae, the subfamilies Macellicephalinae Hartmann-Schröder, 1971 and Lepidonotopodinae Pettibone, 1983 are composed of scaleworms inhabiting extreme marine environments, including anchialine caves and deep-sea ecosystems. In 2020, Hatch et al. [2] emended Lepidonotopodinae to include members of a clade of deep-sea scaleworms inhabiting chemosynthetic-based ecosystems (CBEs), such as methane seeps, hydrothermal vents, and organic falls, based on a phylogeny constructed with six DNA loci obtained via Sanger sequencing [2]. Lepidonotopodinae currently includes eight scaleworm genera, including both non-branchiate (*Bathykurila* Pettibone, 1976, *Lepidonotopodium* Pettibone, 1983, and *Levensteiniella* Pettibone, 1985) and branchiate (*Branchinotogluma* Pettibone, 1985, *Branchiplicatus* Pettibone, 1985, *Branchipolynoe* Pettibone, 1984, *Peinaleopolynoe* Desbruyères & Laubier, 1988, and *Thermopolynoe* Miura, 1994) taxa.

More recently, Gonzalez et al. [3] published phylogenomic analyses of Polynoidae based on 12 transcriptomes, which consistently recovered the included Lepidonotopodinae taxa (*Branchipolynoe pettiboneae* Miura & Hashimoto, 1991 and *Lepidonotopodium* sp.) as nested within Macellicephalinae, indicating a need for a taxonomic reassessment of these clades. Furthermore, several phylogenetic studies [2,4,5,6,7,8,9] have corroborated the non-monophyly of certain Lepidonotopodinae genera. Phylogenetic analyses performed in Zhang et al. [4] and Hatch et al. [2] recovered *Branchinotogluma* as a non-monophyletic group. In the former study, *Branchinotogluma japonicus* (Miura & Hashimoto, 1991) and *Branchinotogluma sandersi* Pettibone, 1985 formed a paraphyletic grade with respect to a clade of *Branchipolynoe* species [4]. In the latter study, *Branchinotogluma* was scattered across Lepidonotopodinae in seven different positions; additionally, non-monophyly of *Lepidonotopodium* was determined [2]. However, no taxonomic revisions were made in either study. Uncertainties regarding the precise delineation of these genera persist and have been since amplified by the description of several new *Branchinotogluma* species from deep-sea CBEs [5,6,9,10]. These findings call for long-overdue taxonomic revisions of deep-sea scaleworms at the subfamiliar and generic levels.

The most recent molecular phylogenies of Lepidonotopodinae [2,6,10] included DNA data for representatives of all eight genera comprised within the subfamily. However, species-level sampling was relatively low. Herein, we generate new molecular data which, alongside previous sequences, allows us to build phylogenies that incorporate 46 known and several new Lepidonotopodinae species. The former includes *Branchinotogluma marianus* (Pettibone, 1989), *Branchinotogluma segonzaci* (Miura & Desbruyères, 1995), *Branchinotogluma tunnicliffeae* (Pettibone, 1988), *Lepidonotopodium* cf. *riftense* Pettibone, 1984, *Lepidonotopodium piscesae* Pettibone, 1988, *Lepidonotopodium williamsae* Pettibone, 1984, *Levensteiniella intermedia* Pettibone, 1990, *Levensteiniella kincaidi* Pettibone, 1985, and *Levensteiniella plicata* Hourdez & Desbruyères, 2000, all of which were sampled at or near their corresponding type localities. Furthermore, we also incorporate the type species for seven of the eight genera currently classified within Lepidonotopodinae, allowing for the investigation of the phylogenetic placement of most genera.

Relative to Sanger sequencing technology, which was used in previous phylogenetic studies of Lepidonotopodinae [2,5,6,7,8,9,10], Next Generation Sequencing (NGS) technologies have drastically reduced the cost and time for DNA sequencing, allowing for the sequencing in parallel of millions of small fragments of DNA spanning the target genome [11]. Low-coverage whole genome sequencing, also referred to as “genome skimming”, has become a common NGS tool utilized to sequence millions of fragments of DNA, which can then be used to bioinformatically assemble and annotate full mitochondrial genomes (mitogenomes) with high read coverage and high-quality resolution [12,13,14,15]. As revealed in Gonzalez et al.’s transcriptome-based phylogenomics study, the construction of phylogenies from larger NGS datasets allows for the better resolution of evolutionary relationships and the identification of problematic systematics within unresolved and understudied taxonomic groups, such as Lepidonotopodinae [3]. Therefore, 31 scaleworm species were sequenced via genome skimming in this study, and the data were utilized to assemble their corresponding mitogenomes. Of the 31 species skimmed, 30 species are native to deep-sea habitats and *Eulagisca gigantea* Monro, 1939 is comparatively shallow living. The combination of these newly generated datasets with pre-existing ones allowed for the investigation of the Lepidonotopodinae phylogeny using a matrix with increased coverage, larger representation of deep-sea polynoids, and an expansion of dataset size from the six loci previously used to 18 genes.

In this study, we resolve the previously noted non-monophyly of *Branchinotogluma* and *Lepidonotopodium*, as well as the genus *Levensteiniella,* which is shown here to be non-monophyletic as well, by erecting five new genera, *Cladopolynoe* gen. nov., *Mamiwata* gen. nov., *Photinopolynoe* gen. nov., *Stratigos* gen. nov., and *Themis* gen. nov., as well as emending diagnoses for *Branchinotogluma, Branchipolynoe*, *Lepidonotopodium*, and *Levensteiniella*. Additionally, we use well-supported phylogenetic results to regard Lepidonotopodinae as a junior synonym of Macellicephalinae and apply the tribe name Lepidonotopodini Pettibone, 1983 to a clade exclusively inhabiting deep-sea CBEs.

In addition to using the aphroditiform mitogenomes for phylogenetics and systematics, we characterize the distinct mitochondrial gene orders present within Aphroditiformia and identify the most parsimonious gene order rearrangement events. Furthermore, we explore the molecular evolution of mitogenomes in anchialine cave-dwelling and deep-sea polynoids. Although the deep sea is generally characterized by immense pressure, perpetual darkness, scarcity of food (non-CBEs), extreme temperatures (4 °C average), and variable oxygen levels; it is also largely heterogeneous, comprising diverse ecosystems with additional selective pressures [16,17,18,19]. For example, deep-sea CBEs may contain steep temperature gradients [19,20,21] and high concentrations of dissolved metals and toxic substances (e.g., hydrogen sulfide and methane) that are lethal to most aerobic metazoans [19]. Anchialine cave systems share intriguing connections to the deep sea: isolation from surface conditions, complete darkness, limited food resources, hypoxic zones, chemosynthesis by chemoautotrophs, steep chemoclines, and the presence of hydrothermal or volcanic features in some cases [22,23,24,25]. To investigate the mitogenomic adaptations of polynoids to these extreme conditions, we compared the substitution rates of mitochondrial protein-coding genes (PCGs) in non-extreme vs. extreme species (native to anchialine caves or deep-sea habitats) along both individual branches of the phylogeny and at individual amino acid sites.

## 2. Materials and Methods

### 2.1. Sample Collection and Morphology

Polynoids were collected at various localities on research cruises from 2005 to 2021 using remotely operated vehicles (ROVs) and human operated vehicles (HOVs) (Table 1). Table S1 provides the additional collection locality details, deposition of vouchers, and GenBank accession numbers for all polynoids analyzed herein. *Branchinotogluma sandersi*, *Branchinotogluma* sp. nov. 1, *Branchinotogluma* sp. nov. 2, and *Lepidonotopodium williamsae* are deposited at Harvard University’s Museum of Comparative Zoology, Invertebrate Zoology (MCZ-IZ), Cambridge, MA, USA. All remaining specimen vouchers are deposited at the Scripps Institution of Oceanography, Benthic Invertebrate Collection (SIO-BIC), La Jolla, CA, USA.

Prior to preservation, whole specimens were generally relaxed with 7% MgCl_2_ in fresh water and photographed alive using either Leica MZ8 or MZ9.5 stereomicroscopes (Leica Microsystems Inc., Deerfield, IL, USA) with a Canon EOS Rebel T6i attachment (Canon USA Inc., Huntington, NY, USA) or a Canon EOS M5 digital camera (Canon USA Inc., Huntington, NY, USA). They were then fixed in either 95% ethanol for DNA extraction or 10% formaldehyde in filtered seawater for morphological work. For those fixed in formalin, the tissue subsamples of elytra or parapodia were also fixed in 95% ethanol for DNA extraction. After a day, the specimens fixed in formalin were rinsed and transferred to 50% ethanol for long-term preservation.

### 2.2. DNA Extraction, Single-Locus Amplification, and Sanger Sequencing

DNA from specimens fixed and preserved in 95% ethanol was extracted using the Zymo Research Quick-DNA^TM^ Miniprep or Microprep Plus Kit (Zymo Research, Irvine, CA, USA), following the protocol supplied by the manufacturer. Specimens of *Branchinotogluma segonzaci* (SIO-BIC A13118), *Lepidonotopodium* cf. *riftense* (SIO-BIC A13202), *Lepidonotopodium piscesae* (SIO-BIC A7978), *Levensteiniella plicata* (SIO-BIC A14234), *Macellicephaloides alvini* (SIO-BIC A14221), and *Themis* sp. nov. (SIO-BIC A14236) were sequenced for nuclear *18S rRNA* (*18S*). The latter three species were also sequenced for nuclear *28S rRNA* (*28S*) and *histone h3* (*H3*). Amplification was carried out using a PCR mixture of 12.5 µL Apex 2.0x Taq Red DNA Polymerase Master Mix (Genesee Scientific, El Cajon, CA, USA) or 12.5 µL Conquest PCR 2.0x Master Mix 1 (Lamda Biotech, St. Louis, MO, USA) when amplification failed with the former reagent, 1 µL each of the appropriate forward and reverse primers (10 µM), 8.5 µL of ddH_2_O, and 2 µL of eluted DNA. DNA sequencing was completed with the following PCR primers and temperature profiles, performed in a thermal cycler (Eppendorf North America, Enfield, CT, USA).

Up to 1972 nucleotide base pairs (nt bp) of *18S* were amplified using the following primer sets and reaction protocols. 18S-1F/18S-5R [39] and 18S-a2.0 [40]/18S-9R [39]: 95 °C/180 s–(95 °C/30 s–50 °C/30 s–72 °C/90 s) × 40 cycles–72 °C/480s. 18S-3F [39]/18S-bi [40]: 95 °C/180 s–(95 °C/30 s–52 °C/30 s–72 °C/90 s) × 40 cycles–72 °C/480 s. Up to 1102 nt bp of *28S* were amplified using the primer set Po28F1/Po28R4 [41] with the reaction protocol: 95 °C/180 s–(95 °C/30 s–55 °C/40 s–72 °C/75 s) × 40 cycles–72 °C/300 s. Up to 333 nt bp of *H3* were amplified using the primer set H3F/H3R [42] with the reaction protocol: 95 °C/180 s–(95 °C/30 s–53 °C/45 s–72 °C/45 s) × 40 cycles–72 °C/300 s.

Furthermore, a specimen of *Branchinotogluma marianus* (SIO-BIC A16396) was sequenced for mitochondrial *cytochrome c oxidase subunit I* (*COX1*) and nuclear *H3*. Given that DNA quality was degraded, primers were designed to amplify minimal sequence data for the two genes. Amplification of *COX1* was carried out using a PCR mixture of PuReTaq^TM^ Ready-To-Go^TM^ PCR Beads (Cytiva, Marlborough, MA, USA), 1 µL of 10 µM COX1-148For (5′-GGA GCC CCT GAT ATA GCT TTC C-3′), 1 µL of 10 µM COX1-267Rev (5′-TGT TCA ACC AGT ACC AGC ACC-3′), 20 µL of ddH_2_O, and 3 µL of eluted DNA. Amplification of *H3* was carried out using a PCR mixture of 12.5 µL Conquest PCR 2.0x Master Mix 1 (Lamda Biotech, St. Louis, MO, USA), 1 µL of 10 µM H3-153For (5′-GAT CCG TCG TTA CCA AAA GTC-3′), 1 µL of 10 µM H3-300Rev (5′-GTA AGC CTC GCT CGC CTC-3′), 6.5 µL of ddH_2_O, and 4 µL of eluted DNA.

Up to 85 nt bp of *COX1* were amplified with the reaction protocol: 95 °C/180 s–(95 °C/30 s–54 °C/45 s–72 °C/45 s) × 40 cycles–72 °C/300 s. Up to 149 nt bp of *H3* were amplified with the reaction protocol: 95 °C/180 s–(95 °C/30 s–52 °C/45 s–72 °C/45 s) × 40 cycles–72 °C/300 s. *COX1* and *H3* primers were designed with *Design New Primers* in Geneious Prime v. 2022.1.1 [43] with *Branchinotogluma* cf. *marianus* sequences used as the template.

Final PCR products were purified with the ExoSAP-IT protocol (USB Affymetrix, Cleveland, OH, USA) and Sanger sequencing was performed by Eurofins Genomics (Louisville, KY, USA). Consensus sequences for mitochondrial *COX1* and nuclear *18S*, *28S*, and *H3* loci were created via *de novo* assembly in Geneious Prime v. 2022.1.1 [43] with default settings. All new sequences obtained via Sanger sequencing are deposited in GenBank and identified in Table S1 with an asterisk next to the corresponding accession number.

### 2.3. Whole Genome Library Preparation and Next Generation Sequencing

Prior to genome sequencing, total DNA concentration for 31 genomic DNA (gDNA) extractions was estimated using the Qubit dsDNA BR Assay Kit with a Qubit fluorometer (Invitrogen, Waltham, MA, USA). Additionally, DNA quality was assessed for samples with agarose gel electrophoresis at 100 V for 65 min. Libraries for *Branchinotogluma sandersi* and *Branchiplicatus cupreus* were self-prepared using the KAPA HyperPlus Kit (KAPA Biosystems, Wilmington, MA, USA) following the manufacturer’s instructions, including sample customization based on total gDNA quantity and quality values. An insert size of circa 500 base pairs (bp) was targeted using enzymatic fragmentation, and adapter ligation was performed with universal iTru Y-yoke adapter “stubs” [44]. Libraries were amplified using paired-end, uniquely dual-indexed iTru5 and iTru7 primers [44] containing custom 8-nucleotide (nt) sequence tags developed by Faircloth & Glenn [45]. Post-amplification, concentration, quality, and molecular weight distribution of gDNA libraries (range = 500–565 bp) was assessed using qPCR and a Genomic DNA1000 ScreenTape (Agilent Technologies, Santa Clara, CA, USA) with an Agilent 4200 TapeStation (Agilent Technologies, Santa Clara, CA, USA) at the IGM Genomics Center (University of California San Diego, La Jolla, CA, USA). *Branchinotogluma sandersi* and *Branchiplicatus cupreus* were sequenced on the Illumina MiSeq (Illumina, San Diego, CA, USA) at the IGM Genomics Center (University of California San Diego, La Jolla, CA, USA), resulting in a sequencing depth of 6,590,612 and 392,731 reads, respectively (Table S2).

After checking that the quantity and quality values for the remaining 29 gDNA extractions met acceptable standards for library preparation, these samples were shipped overnight on dry ice to Novogene Corporation Inc. (Sacramento, CA, USA), where the following molecular lab services were performed. DNA sample QC was assessed using agarose gel electrophoresis, and DNA sample quantification was assessed using a Qubit Fluorometer (Invitrogen, Waltham, MA, USA). Animal whole genome libraries were prepared targeting an insert size of circa 350 bp, and approximately 2 Gb of raw data per sample were sequenced on the Illumina NovaSeq 6000 (Illumina, San Diego, CA, USA) using 150 bp paired-end reads. Samples had on average 10.1 million paired-end reads (range = 5.9–15.9 million reads; Table S2). Raw genome skimming datasets have been deposited in the National Center for Biotechnology Information (NCBI) sequence read archive (SRA) with BioProject accession number PRJNA994258, which includes BioSample accession numbers SAMN36418903-SAMN36418933.

### 2.4. Mitogenome Assembly and Annotation

Raw genome skimming data statistics, including total number and total length of paired-end reads, and average individual length of raw reads, were obtained using SeqKit v. 0.13.2 [46] with the *stats* command and recorded in Table S2. Sequence reads were trimmed (leading and trailing low quality or N bases below quality 3 were removed; reads were scanned with a 4-base wide sliding window and cut when the average quality per base dropped below 15; and reads under 36 bp long were dropped) and cleaned of adapters using Trimmomatic v. 0.39 [47]. Number of reads and percentage of raw reads retained are recorded in Table S2. Mitogenomes were assembled and annotated using MitoFinder v. 1.4 [15], with The Invertebrate Mitochondrial Code (NCBI; *transl_table = 5*) specified as the organism genetic code used for translation of the 13 PCGs. Either MEGAHIT v. 1.2.9 [48] or metaSPAdes v. 3.13.0 [49] metagenomic assemblers and ARWEN v. 1.2 [50] or MiTFi v. 0.1 [51] tRNA gene annotators were specified. Complete records for all RefSeq annelid mitogenomes publicly available on NCBI (79 total; accessed 7 November 2020) were used as the reference file.

Unlike the other species, the assembly of *Branchinotogluma sandersi* and *Branchinotogluma* sp. nov. 2 failed with both metagenomic assemblers using post-Trimmomatic reads as input. Therefore, post-Trimmomatic reads for these species were down-sampled using the *downsample.py* script included with MITObim v. 1.9.1 [13] and post-processed with the *reformat.sh* script included with BBMap v. 38.87 [52]. Mitogenomes were then successfully assembled via MitoFinder v. 1.4 with metaSPAdes v. 3.13.0, using the down-sampled reads as input. MitoFinder v. 1.4 parameters and resulting mitogenome statistics (circularization, mitogenome length and coverage, and GC/AT content) for each species are recorded in Table S3. Resulting mitochondrial genes recovered in the mitogenome contigs were checked for contamination using NCBI’s Nucleotide BLAST online database [53,54,55,56,57].

Resulting MitoFinder v. 1.4 annotations were checked against those obtained using MITOS WebServer [58]. The boundaries of PCGs and ribosomal RNAs (rRNAs) were determined in Geneious Prime v. 2022.1.1 [43] by alignment with the corresponding genes from scaleworm mitogenomes published in Zhang et al. [4], and they were manually edited to reflect the final annotations. Incomplete or missing genes (if any) in the final mitogenome contigs are recorded in Table S3. All newly assembled and annotated mitogenomes obtained in this study are deposited in GenBank (Table S1). *Branchinotogluma* sp. nov. 1 and *Branchinotogluma trifurcus* were each assigned two accession numbers (Table S1) because the corresponding mitogenome data were split into two contigs (Table S3).

### 2.5. Mitogenome Calculations

Nucleotide frequencies for each mitogenome were retrieved from Geneious Prime v. 2022.1.1 statistics files and used for calculating GC and AT contents, as well as GC- and AT-skews (GC-skew = (G − C)/(G + C) and AT-skew = (A − T)/(A + T); Perna & Kocher [59]). Resulting values are recorded in Table S3 for entire mitogenomes and in Table S4 for the 13 PCGs and two rRNAs. Finally, we also estimated codon usage in the 13 PCGs. Preferred (codon bias) and avoided codons per species were investigated using Relative Synonymous Codon Usage (RSCU) values [60] with the CAI calculator online server [61]. Resulting RSCU values and codon frequencies (%) per amino acid are recorded in Table S5. Codons with RSCU values greater than 1.6 or smaller than 0.6 are considered to be over- and under-represented, respectively [62].

### 2.6. Extracting Nuclear Genes from NGS Data

Nuclear *H3*, *18S*, and *28S* genes were extracted from the genome skimming reads of all 31 newly sequenced scaleworms. This relied on sequences for closely related polynoids, retrieved from NCBI as separate FASTA files pertaining to each of the three genes. For all skimmed taxa, their sanitized reads were mapped individually to the *H3*, *18S*, and *28S* reference FASTA files using Minimap2 v. 2.17 [63]. Resulting mapped reads were extracted using SAMtools v. 1.11 [64]. Read coverage was visualized using *Map to Reference* in Geneious Prime v. 2022.1.1, and consensus sequences for each locus were extracted. Nuclear DNA sequences obtained from genome skimming data via this mapping approach are deposited in GenBank (Table S1).

### 2.7. Phylogenetic Analyses

Newly generated data were combined with pre-existing sequences after revising their taxonomic assignments as detailed below:

- An unpublished mitogenome of *Branchinotogluma segonzaci* (GenBank accession number OK136373) and Sanger sequences of *B. segonzaci* generated in Wu et al. [9] and Lee et al. [65] are referred to as *B.* cf. *segonzaci* (Miura & Desbruyères, 1995) because the specimens were collected from the North Fiji Basin and Manus Back-Arc Basin in the West Pacific Ocean, respectively, as opposed to being collected from the type locality of the Lau Back-Arc Basin in the West Pacific Ocean.
- *Lepidonotopodium williamsae* sequences sourced from Goffredi et al. [66] and Hatch et al. [2] are referred to as *L.* cf. *williamsae* Pettibone, 1984, because the specimen was collected from the Alarcon Rise in the Gulf of California, Mexico, as opposed to from the type locality of hydrothermal vents off the Galapagos Islands.
- *Lepidonotopodium* sp. nov. sequences generated in Goffredi et al. [66] and Hatch et al. [2] are referred to as *Levensteiniella kincaidi* because the morphology matches that of the original species description and because the specimen was collected in the Gulf of California, Mexico (23° N), which is geographically close to the type locality of hydrothermal rift areas off of western Mexico (21° N).
- *Bathykurila guaymasensis* Pettibone, 1989 sequences generated in Glover et al. [67] and Hatch et al. [2] are referred to as *Bathykurila* sp. because the specimens were collected from a whalefall in the Santa Cruz Basin (CA, USA) and from Rosebud whalefall off of San Diego (CA, USA), respectively, as opposed to being collected from the type locality at hydrothermal vents in the Guaymas Basin, Gulf of California, Mexico.
- As in Hatch et al. [2], we refer to the *Branchinotogluma sandersi* sequences generated in Norlinder et al. [68] as *B.* cf. *sandersi* Pettibone, 1985 because the specimen was collected from Juan de Fuca vents in the northeast Pacific as opposed to from the type locality along the Galapagos Rift.
- Lastly, we refer to the *Levensteiniella iris* Hourdez & Desbruyères, 2003 sequences generated in Zhang et al. [4] as *L.* cf. *iris* Hourdez & Desbruyères, 2003 because the specimen was collected from East Scotia Ridge in the South Atlantic Ocean as opposed to from the type locality along the Mid-Atlantic Ridge.

Abbreviations for the 13 mitochondrial PCGs and 2 rRNAs are as follows: *ATP synthase F0 subunit 6* (*ATP6*), *ATP synthase F0 subunit 8* (*ATP8*), *cytochrome c oxidase subunit I* (*COX1*), *cytochrome c oxidase subunit II* (*COX2*), *cytochrome c oxidase subunit III* (*COX3*), *cytochrome b* (*CYTB*), *NADH dehydrogenase subunit 1* (*ND1*), *NADH dehydrogenase subunit 2* (*ND2*), *NADH dehydrogenase subunit 3* (*ND3*), *NADH dehydrogenase subunit 4* (*ND4*), *NADH dehydrogenase subunit 4L* (*ND4L*), *NADH dehydrogenase subunit 5* (*ND5*), *NADH dehydrogenase subunit 6* (*ND6*), *12S rRNA* (*12S* or *rrnS*), and *16S rRNA* (*16S* or *rrnL*). Abbreviations for the 22 transfer RNAs (tRNAs) are as follows: *tRNA-Ala* (*A*), *tRNA-Cys* (*C*), *tRNA-Asp* (*D*), *tRNA-Glu* (*E*), *tRNA-Phe* (*F*), *tRNA-Gly* (*G*), *tRNA-His* (*H*), *tRNA-Ile* (*I*), *tRNA-Lys* (*K*), *tRNA-Leu* (*L1*), *tRNA-Leu2* (*L2*), *tRNA-Met* (*M*), *tRNA-Asn* (*N*), *tRNA-Pro* (*P*), *tRNA-Gln* (*Q*), *tRNA-Arg* (*R*), *tRNA-Ser* (*S1*), *tRNA-Ser2* (*S2*), *tRNA-Thr* (*T*), *tRNA-Val* (*V*), *tRNA-Tyr* (*Y*), and *tRNA-Trp* (*W*).

#### 2.7.1. Six-Gene Macellicephalinae Phylogeny

**Figure A1:**
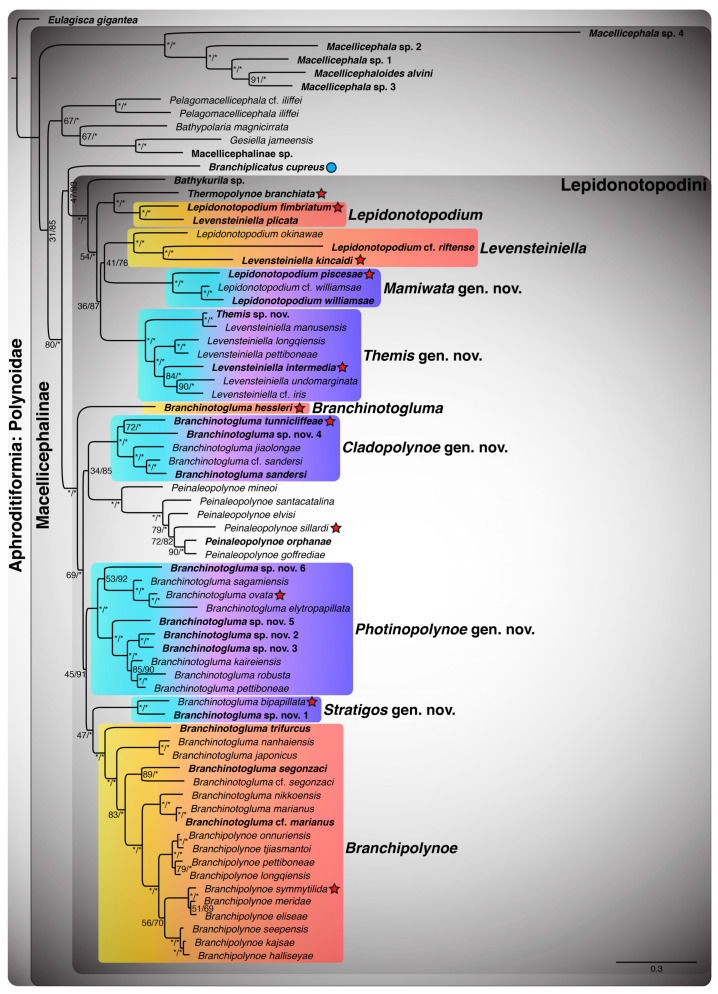
Macellicephalinae maximum likelihood (ML) tree resulting from the combined analysis of six nucleotide (NT) alignments, which is referred to as the 6NT analysis. Numbers next to nodes are ML bootstrap and Bayesian inference (BI) posterior probabilities, both expressed as percentages. Key: * indicates support values ≥ 95%. The five new Lepidonotopodini genera that are erected herein are highlighted in blue/purple. Four existing Lepidonotopodini genera that are emended herein are highlighted in yellow/orange. If present, type species of Lepidonotopodini genera are indicated with a red star. The blue circle next to *Branchiplicatus cupreus* indicates that it is not included in Lepidonotopodini due to its uncertain phylogenetic position. Taxon names set in bold denote the 31 scaleworm species for which new mitochondrial genomes (mitogenomes) were assembled.

**Figure A2:**
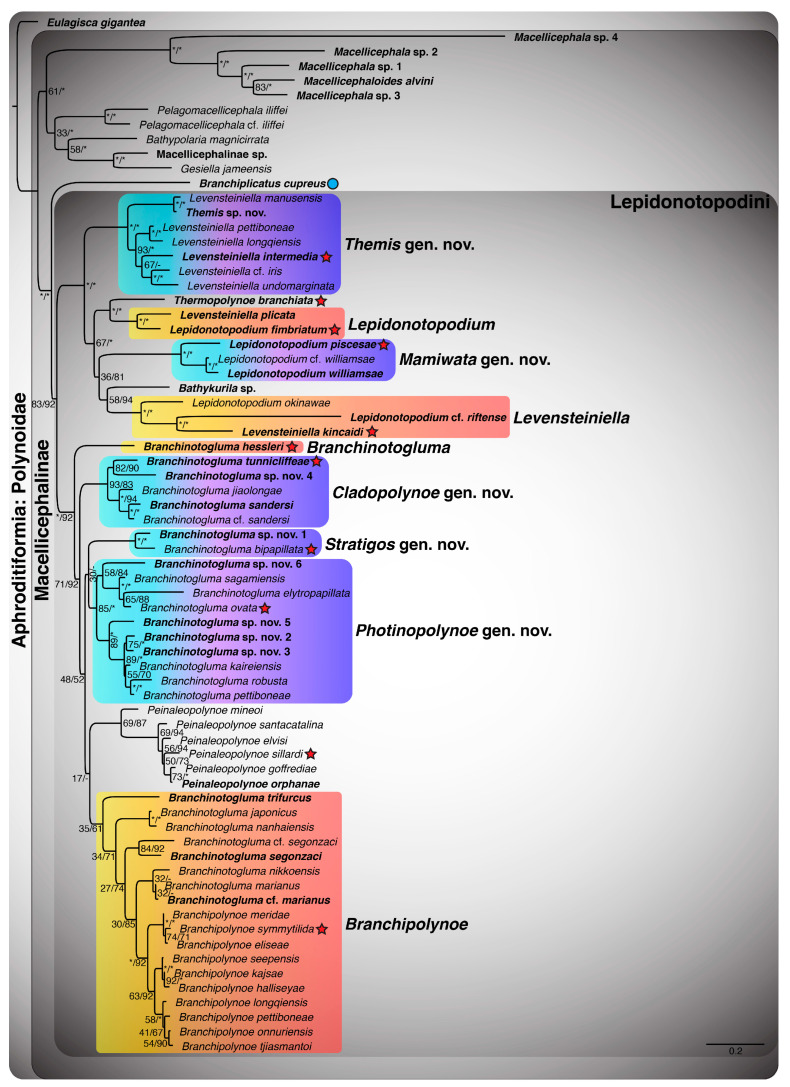
Macellicephalinae ML tree resulting from the combined analysis of three NT alignments and three amino acid (AA) alignments, which is referred to as the 3NT-3AA analysis. Numbers next to nodes are ML bootstrap and BI posterior probabilities, both expressed as percentages. Key: * indicates support values ≥ 95%; - indicates the node was not found. The five new Lepidonotopodini genera that are erected herein are highlighted in blue/purple. Four existing Lepidonotopodini genera that are emended herein are highlighted in yellow/orange. If present, type species of Lepidonotopodini genera are indicated with a red star. The blue circle next to *Branchiplicatus cupreus* indicates that it is not included in Lepidonotopodini due to its uncertain phylogenetic position. Taxon names set in bold denote the 31 scaleworm species for which new mitogenomes were assembled.

A six-gene NT dataset (mitochondrial *COX1*, *16S*, and *CYTB*, and nuclear *18S*, *28S*, and *H3*) was generated by combining the newly generated sequences with data available on GenBank from several different studies (Table S1). Alignments were performed in Mesquite v. 3.61 [69] for each gene using MAFFT v. 7 [70] with the G-INSI-i method. The PCGs (*COX1*, *CYTB*, and *H3*) were translated into amino acids (AAs) in Geneious Prime v. 2022.1.1 using either the invertebrate mitochondrial (*COX1* and *CYTB*) or standard (*H3*) genetic codes, with final stop codons removed. The resulting AA sequences were aligned for each loci in Mesquite v. 3.61 using the MUSCLE [71] algorithm with default settings. Maximum likelihood (ML) analyses with 100 random starting trees were performed on two separate concatenated datasets using raxmlGUI v. 2.0.10 [72] with RAxML-NG v. 1.1.0 [73]: (1) Six NT alignments (referred to as 6NT; Figure A1), and (2) three NT alignments for rRNAs plus three AA alignments for PCGs (referred to as 3NT-3AA; Figure A2). For both analyses, the data were partitioned by gene, with the best-fit models for these partitions selected using ModelTest-NG v. 0.1.7 [74]. Node support was assessed via non-parametric bootstrapping with 1000 pseudoreplicates.

Additionally, partitioned Bayesian inference (BI) analyses of the concatenated 6NT and 3NT-3AA datasets were conducted using Mr. Bayes v. 3.2.7a [75] with 20,000,000 generations, four parallel chains sampling every 1000th generation, and three independent runs; burn-in was set to 10% after inspection with Tracer v. 1.7.2 [76]. Best-fit models from among those available in Mr. Bayes v. 3.2.7a [75] were selected using ModelFinder [77] in IQ-TREE v. 2.2.0 [78] with the *-mset mrbayes* command. Support was estimated using posterior probabilities (expressed as percentages). Outgroups and best-fit models selected for each locus are listed for all phylogenetic analyses in Table S6.

#### 2.7.2. Eighteen-Gene “Mitogenome” Aphroditiformia Phylogeny

**Figure A3:**
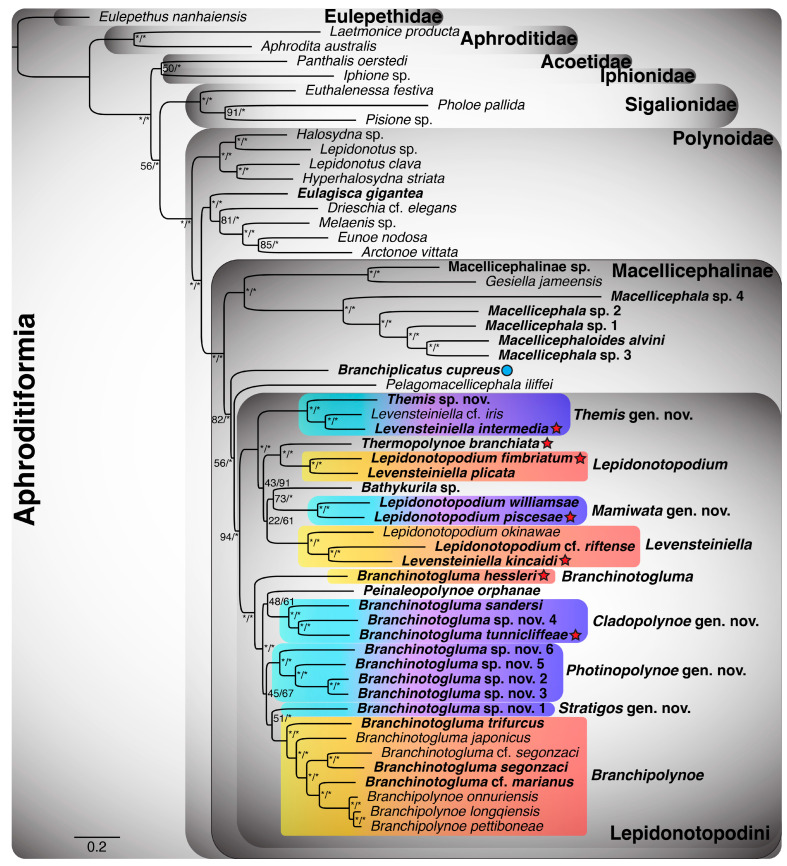
Aphroditiformia ML tree resulting from the combined analysis of 18 NT alignments, which is referred to as the 18NT analysis. Numbers next to nodes are ML bootstrap and BI posterior probabilities, both expressed as percentages. Key: * indicates support values ≥ 95%. The five new Lepidonotopodini genera that are erected herein are highlighted in blue/purple. Four existing Lepidonotopodini genera that are emended herein are highlighted in yellow/orange. If present, type species of Lepidonotopodini genera are indicated with a red star. The blue circle next to *Branchiplicatus cupreus* indicates that it is not included in Lepidonotopodini due to its uncertain phylogenetic position. Taxon names set in bold denote the 31 scaleworm species for which new mitogenomes were assembled.

**Figure A4:**
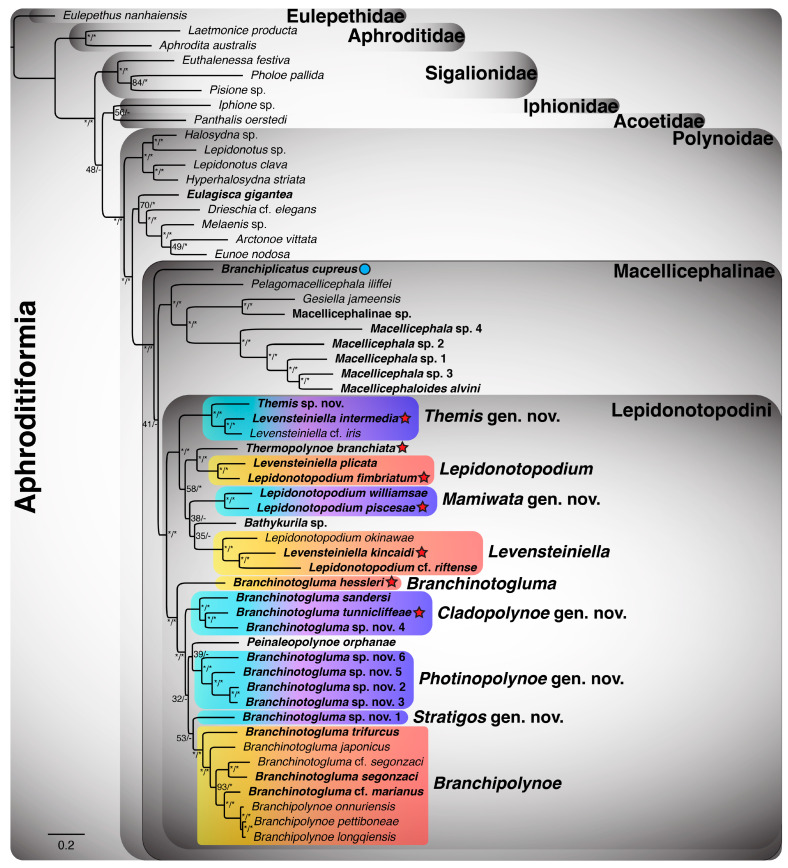
Aphroditiformia ML tree resulting from the combined analysis of four NT alignments and 14 AA alignments, which is referred to as the 4NT-14AA analysis. Numbers next to nodes are ML bootstrap and BI posterior probabilities, both expressed as percentages. Key: * indicates support values ≥ 95%; - indicates the node was not found. The five new Lepidonotopodini genera that are erected herein are highlighted in blue/purple. Four existing Lepidonotopodini genera that are emended herein are highlighted in yellow/orange. If present, type species of Lepidonotopodini genera are indicated with a red star. The blue circle next to *Branchiplicatus cupreus* indicates that it is not included in Lepidonotopodini due to its uncertain phylogenetic position. Taxon names set in bold denote the 31 scaleworm species for which new mitogenomes were assembled.

An 18-gene “mitogenome” Aphroditiformia dataset was built for the 56 species with available mitogenomes (Table S1, see “Mitogenome” column). As before, two datasets were assembled, changing whether the PCGs were NTs (referred to as 18NT; Figure A3) or translated to AAs (referred to as 4NT-14AA; Figure A4). Translation, alignment, model selection, and analysis under ML and BI were performed as described above.

#### 2.7.3. Eighteen-Gene “All Taxa” Aphroditiformia Phylogeny

**Figure 1:**
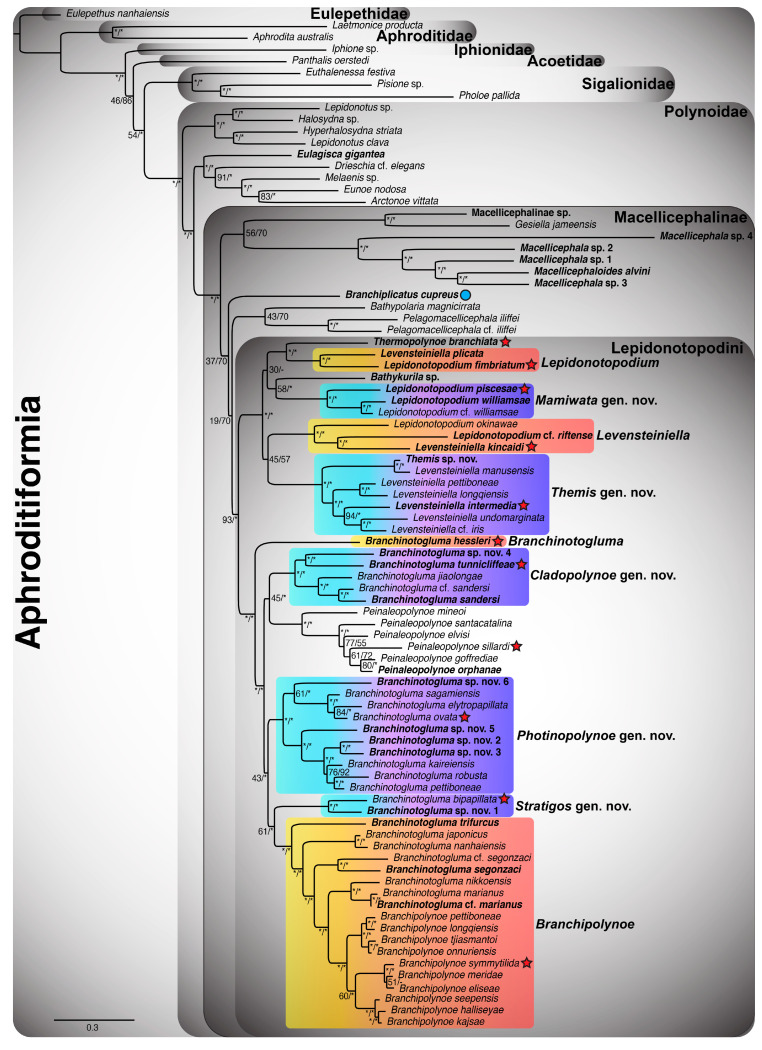
Aphroditiformia maximum likelihood (ML) tree resulting from the combined analysis of 18 nucleotide (NT) alignments, referred to as the 18NT-All-Taxa analysis. All taxa are included in this analysis regardless of whether they were represented by a complete mitochondrial genome (mitogenome) or only Sanger-sequenced individual loci. Numbers next to nodes are ML bootstrap and Bayesian inference (BI) posterior probabilities, both expressed as percentages. Key: * indicates support values ≥ 95%; - indicates the node was not found. The five new Lepidonotopodini genera that are erected herein are highlighted in blue/purple. Four existing Lepidonotopodini genera that are emended herein are highlighted in yellow/orange. Type species of Lepidonotopodini genera are indicated with a red star (all genera except *Bathykurila*). This includes presently named species that are herein designated as the type species of five new genera. The blue circle next to *Branchiplicatus cupreus* indicates that it is not included in Lepidonotopodini, owing to its uncertain phylogenetic position (see Figure 2). Taxon names set in bold denote the 31 scaleworm species for which new mitogenomes were assembled.

**Figure 2:**
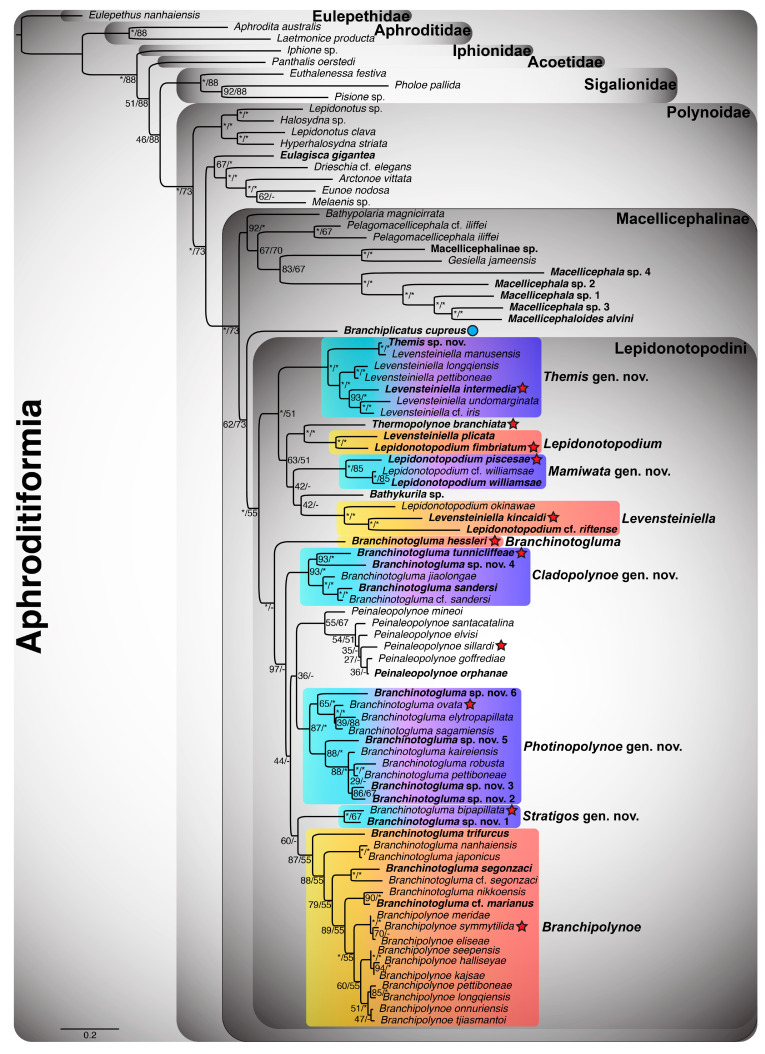
Aphroditiformia ML tree resulting from the combined analyses of 4 NT alignments and 14 amino acid (AA) alignments, referred to as the 4NT-14AA-All-Taxa analysis. Analytical details and key are as detailed in the caption of Figure 1. *Branchinotogluma marianus* was pruned from this figure, as its position was unstable in the 4NT-14AA-All-Taxa analysis.

An expanded 18-gene “all taxa” Aphroditiformia dataset was built by combining all of the mitogenomes with the data for species represented only with Sanger sequences, resulting in 87 terminals in total. The same approach and methods as previously described were followed to produce two datasets, referred to as 18NT-All-Taxa (Figure 1) and 4NT-14AA-All-Taxa (Figure 2), both of which were analyzed under ML and BI. *Branchinotogluma marianus* was pruned from the 4NT-14AA-All-Taxa tree a posteriori due to its unstable phylogenetic position, a consequence of its highly degraded DNA and the resulting fragmented loci (only 85 nt bp of *COX1* and 149 nt bp of *H3*). This relied on the *drop.tip()* function from the ape v. 5.7-1 [79,80] package in R v. 4.2.3 [81].

### 2.8. Aphroditiformia Mitochondrial Gene Order Analyses

In addition to documenting mitochondrial gene order with and without the inclusion of tRNAs (37 and 15 genes, respectively), the following analyses were performed on both gene order datasets. TreeREx v. 1.85 [82] was used to reconstruct the most parsimonious scenarios of mitochondrial gene order evolution and to determine putative ancestral gene orders. Taxa with missing mitochondrial genes (see Table S3) were pruned from the 4NT-14AA ML tree beforehand. Three different reconstruction settings implemented in TreeREx were explored, including: *-s* (strong consistency method), *-w* (weak consistency method), and *-W* (parsimonious weak consistency method). Subsequently, the CREx [83] webserver was used to conduct pair-wise comparisons (based on common intervals) of the Aphroditiformia mitochondrial gene orders to determine rearrangement events, considering reversals, transpositions, reverse transpositions, and tandem duplication–random losses (TDRLs).

### 2.9. Polynoidae Selection Analyses of Mitochondrial PCGs

The PAML v. 4.10.7 [84] codeml package was used to conduct base substitution analyses for each of the 13 mitochondrial PCGs across taxa with contrasting ecologies. Two alternative categorizations of species were explored, involving comparisons between (A) deep-sea and shallow-water polynoids (using a depth cutoff of 500 m) and (B) polynoids from “extreme” (anchialine caves plus deep-sea habitats) and non-extreme marine environments. In both cases, the former of the two categories was defined as the set of foreground branches. The only difference between these analyses was the categorization of the shallow anchialine cave-dwelling *Gesiella jameensis* (Hartmann-Schröder, 1974) and *Pelagomacellicephala iliffei* Pettibone, 1985, which were either considered background “shallow-water” or foreground “extreme” dwellers. Thus, the “extreme” categorization included all Macellicephalinae taxa, as emended here (see below). PAL2NAL v. 14 [85] was used to convert NT sequences into codon alignments for each PCG with the AA alignments used as input, using the invertebrate mitochondrial code for translation and retaining gaps. Given the large genetic divergence within Aphroditiformia, the unrooted 4NT-14AA ML tree was pruned of aphroditiform taxa not within the family Polynoidae and used as codeml input for the analysis of all PCGs except *ND1*. For this locus, two taxa missing *ND1* sequences were further pruned (*Lepidonotopodium* cf. *riftense* and *Themis* sp. nov.; see Table S3). Foreground branches of the input trees were labeled with ‘#1’ using phylotree.js v. 2.0.1 [86].

Using these data, we performed both branch model and branch-site model A tests. PAML codeml control files that exemplify the parameter settings necessary to replicate all analyses are provided as Supplementary Files S1–S4. For the first, the M0 model (one-ratio) served as the null hypothesis, which assumes one nonsynonymous/synonymous substitutions rate ratio (ω = d_N_/d_S_) for all sites and across all branches [87]. The M2 branch model (two-ratio) served as the alternative hypothesis, which assumes a different ω for background vs. foreground branches, but no difference across sites [87].

For the branch-site model A tests, the alternative hypothesis (M2a model) assumed heterogenous pressure across sites and across branches [87]. This M2a model estimates the proportions for the four site classes assumed (0, 1, 2a, 2b), with details for each site class specified in Álvarez-Carretero et al. [87], along with corresponding ω estimates for each site class. Furthermore, the M2a model reports codon sites that have a probability higher than either 95% or 99% to be under positive selection (ω > 1) in foreground lineages according to the Bayes Empirical Bayes (BEB) analysis [88]. The null hypothesis for the branch-site model A tests did not estimate ω for the foreground branches in site class 2a, but instead fixed ω to 1 [87].

Likelihood ratio tests (LRTs) were performed to compare null vs. alternative models in both branch model and branch-site model A tests using a significance threshold for the chi-square test of *p* < 0.01 [87]. For the branch model tests, genes were considered as undergoing relaxed purifying selection in foreground branches if their estimated ω was larger than for background branches; for the branch-site model A tests, genes were considered as evolving under positive selection in foreground branches if ω > 1 for the proportion of foreground branches in site classes 2a and 2b. Amino acid sites were considered as evolving under positive selection if the above conditions were met for positive selection occurring along the corresponding gene and if BEB values were ≥ 0.99 for the given site, indicating a 99% probability under positive selection (ω > 1) in foreground lineages.

Using the same codon alignments and input trees, except for foreground branches labeled with ‘{Foreground}’ as opposed to ‘#1’ using phylotree.js v. 2.0.1 [86], selection analyses were run with Hypothesis Testing using Phylogenies (HyPhy) v. 2.5.62 [89,90]. For all HyPhy analyses, *--code Invertebrate-mtDNA* was specified and four replicates were run of the following tests for each mitochondrial PCG: adaptive Branch-Site Random Effects Likelihood (aBSREL) v. 2.5 [91], Branch-Site Unrestricted Statistical Test for Episodic Diversification (BUSTED) v. 4.5 [92], and Mixed Effects Model of Evolution (MEME) v. 4.0 [93]. Evidence of positive selection in foreground lineages at the level of phylogenetic branches (aBSREL), entire PCGs (BUSTED), or individual sites (MEME) was considered only in cases where the same result was obtained across all four replicates at *p* < 0.01.

## 3. Results

### 3.1. Phylogenetic Results

Given that the 18-gene “all taxa” Aphroditiformia phylogenies (18NT-All-Taxa and 4NT-14AA-All-Taxa) contained the greatest number of genes and taxa, the results of these trees will be discussed in detail. All other phylogenetic results are reported in Figure A1, Figure A2, Figure A3 and Figure A4. The ML analyses of the 18NT-All-Taxa (Figure 1) and the 4NT-14AA-All-Taxa (Figure 2) datasets recovered the same phylogenetic relationships among all of the included shallow-water Aphroditiformia families. When rooted with a member of Eulepethidae Chamberlin, 1919, the families Aphroditidae Malmgren, 1867, Iphionidae Kinberg, 1856, Acoetidae Kinberg, 1856, and Sigalionidae Kinberg, 1856 formed successive branching clades subtending Polynoidae, the monophyly of which was strongly supported. Shallow-water polynoids formed two clades that were well-supported: one containing *Halosydna* sp., *Hyperhalosydna striata* (Kinberg, 1856), *Lepidonotus clava* (Montagu, 1808), and *Lepidonotus* sp., whose relationships proved stable across analyses; and another including *Arctonoe vittata* (Grube, 1855), *Drieschia* cf. *elegans* Seidler, 1924, *Eulagisca gigantea*, *Eunoe nodosa* (M. Sars, 1861), and *Melaenis* sp., whose internal organization showed weaker support and less stability across analyses. The latter of these formed the sister group to a revised Macellicephalinae (Figure 1, Figure 2, Figure A1, Figure A2, Figure A3 and Figure A4), representing a strongly supported clade including deep-sea and anchialine cave-dwelling polynoids.

Initial splits within Macellicephalinae proved unstable and hard to resolve with confidence. In the 4NT-14AA-All-Taxa ML tree (Figure 2), a single clade containing *Bathypolaria magnicirrata* (Neal, Barnich, Wiklund & Glover, 2012), *Gesiella jameensis*, *Macellicephala* sp. 1–4, Macellicephalinae sp., *Macellicephaloides alvini*, *Pelagomacellicephala* cf. *iliffei*, and *P. iliffei* was recovered as a sister to the subfamily Lepidonotopodinae, as delineated by Hatch et al. [2], i.e., including the genera *Bathykurila*, *Branchinotogluma*, *Branchiplicatus*, *Branchipolynoe*, *Lepidonotopodium*, *Levensteiniella*, *Peinaleopolynoe*, and *Thermopolynoe*. However, in the 18NT-All-Taxa ML tree (Figure 1), these taxa are reorganized in a manner that renders Lepidonotopodinae *sensu* Hatch et al. [2] paraphyletic, requiring either the removal of *Branchiplicatus* from the subfamily or the incorporation of *Bathypolaria* and *Pelagomacellicephala*. Given the low support values and unstable phylogenetic position of *Branchiplicatus cupreus* (indicated with a blue circle next to the taxon name in Figure 1, Figure 2, Figure A1, Figure A2, Figure A3 and Figure A4), we favor the former option and instate the tribe Lepidonotopodini, containing all of the taxa previously classified as Lepidonotopodinae *sensu* Hatch et al. [2], with the exception of *Branchiplicatus cupreus*. Lepidonotopodini includes deep-sea polynoids known exclusively from CBEs. Within Lepidonotopodini, two well-supported clades were recovered as sister to each other in both 18-gene “all taxa” trees (Figure 1 and Figure 2).

One clade contains *Bathykurila*, *Lepidonotopodium*, and *Levensteiniella* (all of which are non-branchiate taxa), plus *Thermopolynoe branchiata*. *Lepidonotopodium* and *Levensteiniella* were recovered as non-monophyletic, requiring the erection of two new genera: *Mamiwata* gen. nov., containing *Lepidonotopodium piscesae* (type species; see Table 2), *Lepidonotopodium* cf. *williamsae*, and *Lepidonotopodium williamsae*; and *Themis* gen. nov., containing *Levensteiniella intermedia* (type species; see Table 2), *Levensteiniella* cf. *iris, Levensteiniella longqiensis* Han, Zhou, Chen & Wang, 2023, *Levensteiniella manusensis Wu & Xu*, 2018, *Levensteiniella pettiboneae* Han, Zhou, Chen & Wang, 2023, *Levensteiniella undomarginata* Zhang, Chen & Qiu, 2018, and *Themis* sp. nov. *Lepidonotopodium* is emended here to include *Lepidonotopodium fimbriatum* (type species) and *Levensteiniella plicata*. *Levensteiniella* is emended to include *Levensteiniella kincaidi* (type species), *Lepidonotopodium* cf. *riftense*, and *Lepidonotopodium okinawae* Sui & Li, 2017. Relationships among these six genera (*Bathykurila*, *Lepidonotopodium*, *Levensteiniella*, *Mamiwata* gen. nov., *Themis* gen. nov., and *Thermopolynoe*) were poorly resolved, yet their respective monophyly was strongly corroborated by all analyses.

The second clade within Lepidonotopodini contains *Branchinotogluma*, *Branchipolynoe*, and *Peinaleopolynoe*, all of which are branchiate deep-sea polynoids. *Branchinotogluma* was recovered as non-monophyletic, with the type species *B. hessleri* placed by itself as sister to the remaining taxa in this clade. *Branchinotogluma* is thus emended here to be monotypic. The remaining *Branchinotogluma* species were recovered within four clades, requiring the erection of three new genera (*Cladopolynoe* gen. nov., *Photinopolynoe* gen. nov., and *Stratigos* gen. nov.) and the amendment of *Branchipolynoe*. *Cladopolynoe* gen. nov. consists of a clade including *Branchinotogluma tunnicliffeae* (type species; see Table 2), *Branchinotogluma* cf. *sandersi*, *Branchinotogluma jiaolongae* Han, Zhou, Chen & Wang, 2023, *Branchinotogluma sandersi*, and *Branchinotogluma* sp. nov. 4. *Photinopolynoe* gen. nov. consists of two smaller clades sister to one another: a clade with *Branchinotogluma ovata* Wu, Zhan & Xu, 2019 (type species; see Table 2), *Branchinotogluma elytropapillata* Zhang, Chen & Qiu, 2018, *Branchinotogluma sagamiensis* Jimi, Chen & Fujiwara, 2022, and *Branchinotogluma* sp. nov. 6, and a clade with *Branchinotogluma kaireiensis* Han, Zhou, Chen & Wang, 2023, *Branchinotogluma pettiboneae* Wu, Zhan & Xu, 2019, *Branchinotogluma robusta* Wu, Zhen, Kou & Xu, 2023, *Branchinotogluma* sp. nov. 2, *Branchinotogluma* sp. nov. 3, and *Branchinotogluma* sp. nov. 5. *Stratigos* gen. nov. is a small clade incorporating *Branchinotogluma bipapillata* Zhou, Wang, Zhang & Wang, 2018 (type species; see Table 2) and *Branchinotogluma* sp. nov. 1. Finally, *Branchipolynoe* is revised to include eight free-living *Branchinotogluma* taxa (*B.* cf. *marianus*, *B.* cf. *segonzaci*, *B. japonicus*, *B. marianus*, *Branchinotogluma nanhaiensis* Wu, Zhen, Kou & Xu, 2023, *Branchinotogluma nikkoensis* Jimi, Chen & Fujiwara, 2022, *B. segonzaci*, and *B. trifurcus*) that form a grade with respect to a well-supported clade containing the ten currently described symbiotic *Branchipolynoe* taxa. *Peinaleopolynoe*, on the other hand, was always recovered as monophyletic, with high support in the 18NT-All-Taxa ML tree (Figure 1).

The main phylogenetic structure within Lepidonotopodini (as delineated here) was stable across analyses (Figure 1 and Figure 2), composed of two major clades incorporating 12 genera (as defined above). Furthermore, only two of these genera were resolved in different positions between the 18NT-All-Taxa (Figure 1) and 4NT-14AA-All-Taxa (Figure 2) ML trees: *Levensteiniella* (sister group to either *Themis* gen. nov. or *Bathykurila*) and *Peinaleopolynoe* (sister group to *Cladopolynoe* gen. nov. or *Photinopolynoe* gen. nov.). Removal of these two clades rendered all other genus-level relationships within Figure 1 and Figure 2 identical, although not always exhibiting high support. Internal relationships within the smaller Lepidonotopodini clades *Cladopolynoe* gen. nov., *Lepidonotopodium*, *Levensteiniella*, *Mamiwata* gen. nov., *Peinaleopolynoe*, and *Themis* gen. nov. were stable. *Photinopolynoe* gen. nov. comprised two smaller clades composed of identical taxa in both analyses, yet species-level relationships varied between them. Within *Branchipolynoe*, relationships amongst the ten symbiotic taxa were also unstable. Henceforth, terminals will be referred to using their new combinations or amended names.

### 3.2. Taxonomy

**Polynoidae Kinberg, 1856** [94]
**Macellicephalinae Hartmann-Schröder, 1971** [95]

**Type genus:** *Macellicephala* McIntosh, 1885 [96].

**Diagnosis (emended):** Polynoids inhabiting shallow-water anchialine caves and deep-sea environments. Prostomium with paired palps. Median antenna absent or present; lateral antennae absent; minute frontal filaments or frontal horns may be present. Achaetous segment one with a pair of enlarged dorsal and ventral anterior cirri (=tentacular cirri). Notopodia with or without branchiae, either plicate or arborescent if present.

**Remarks:** Hatch et al. [2] applied the subfamily name Lepidonotopodinae to a clade consisting of the following eight deep-sea genera known from CBEs: *Bathykurila*, *Branchinotogluma*, *Branchiplicatus*, *Branchipolynoe*, *Lepidonotopodium*, *Levensteiniella*, *Peinaleopolynoe*, and *Thermopolynoe*. However, with the increased amount of data and taxonomic sampling attained here, it is evident that Lepidonotopodinae should be synonymized with Macellicephalinae for stability of taxonomic names. Therefore, Macellicephalinae is emended to allow for the inclusion of the genera from Hatch et al.’s [2] emended diagnosis of Lepidonotopodinae: *Bathykurila*, *Branchinotogluma*, *Branchiplicatus*, *Branchipolynoe*, *Lepidonotopodium*, *Levensteiniella*, *Peinaleopolynoe*, and *Thermopolynoe*. Furthermore, the following new genera are added to Macellicephalinae: *Cladopolynoe* gen. nov., *Mamiwata* gen. nov., *Photinopolynoe* gen. nov., *Stratigos* gen. nov., and *Themis* gen. nov.

Bonifácio and Menot [97] emended Macellicephalinae to include anchialine cave-dwelling and deep-sea polynoids with or without a median antenna and lacking lateral antennae. Although we agree with this diagnosis, we acknowledge Hatch et al.’s [2] previous argument for frontal filaments/horns being homologous with lateral antennae, given the insertion of these structures terminally on the prostomium, which is the same placement as the lateral antennae in other polynoid subfamilies (e.g., Lepidastheniinae Pettibone, 1989 and Lepidonotinae Wiley, 1902). However, this homology is uncertain given the description of the unique polynoid *Bathynotalia perplexa* Levenstein, 1982, which has both frontal filaments (inserted terminally) and lateral antennae (inserted ventrally) on the prostomium.

**Lepidonotopodini Pettibone, 1983 [33]**

**Diagnosis:** Macellicephalins inhabiting deep-sea CBEs, with 14 to 28 segments and 7 to 11 pairs of elytra and elytrophores; elytra large covering dorsum, moderately large leaving middle of dorsum uncovered, or small leaving a large portion of dorsum uncovered (e.g., symbiotic *Branchipolynoe* species). Elytra smooth (sometimes with branched veins) or with diverse textured morphologies involving micro/macro tubercles, micro/macro papillae, prominent longitudinal folds, and/or foveolae. Nearly unilobed or bilobed prostomium with paired tapering palps; lateral antennae absent; median antenna with ceratophore in anterior notch; frontal filaments absent or present; eyes lacking. Achaetous segment one with a pair of enlarged dorsal and ventral anterior cirri (=tentacular cirri). Notopodia with or without arborescent branchiae. If present, arborescent branchiae in single or paired groups; paired groups separated into either anterior/posterior (e.g., *Thermopolynoe branchiata*) or upper/lower groups on notopodia; branchiae start on segment two or three. Sexual dimorphism, with opposite sexes having diverse morphologies involving presence or absence of ventral segmental papillae and/or lamellae. Pharynx with various numbers of border papillae; minimum three dorsal and two ventral border papillae; maximum nine dorsal and nine ventral border papillae. Two pairs of dorsal and ventral jaws; without teeth (smooth), serrated with very small teeth (minute denticles), or with various numbers and positioning of large distinct teeth.

**Remarks:** As currently delineated, all Lepidonotopodini species possess a median antenna. Contrary to Hatch et al.’s [2] emended diagnosis of Lepidonotopodinae, Lepidonotopodini branchiate polynoids may only possess arborescent branchiae, not plicate branchiae (*Branchiplicatus cupreus*). Furthermore, the tribe Lepidonotopodini is applied to a consistently well-supported clade containing *Bathykurila, Branchinotogluma, Branchipolynoe, Cladopolynoe* gen. nov., *Lepidonotopodium, Levensteiniella, Mamiwata* gen. nov., *Peinaleopolynoe, Photinopolynoe* gen. nov.*, Stratigos* gen. nov., *Themis* gen. nov., and *Thermopolynoe*, which are deep-sea polynoids known from CBEs. *Branchiplicatus cupreus* is not included in Lepidonotopodini due to its uncertain phylogenetic position (Figure 1, Figure 2, Figure A1, Figure A2, Figure A3 and Figure A4). Table S7 contains descriptive information for several morphological traits of species placed within the Lepidonotopodini genera revised or newly erected herein. Table S8 provides morphological diagnostic characters of the 12 Lepidonotopodini genera. Table 2 lists the species placed in each of these genera. *Bathykurila*, *Peinaleopolynoe*, and *Thermopolynoe* are the only Lepidonotopodini genera not emended here.

**Table 2:** Updated classification of previously described species placed within the Lepidonotopodini genera revised or newly erected in this study. Key: ** indicates the type species for each genus, which are also set in bold. -- indicates species that are assigned to the corresponding genus *incertae sedis*, which are also underlined.

Assigned Lepidonotopodini Genus	Previously Described Species	New Combination
***Branchinotogluma*** **Pettibone, 1985** [27]	***Branchinotogluma hessleri*** Pettibone, 1985 ** [27]	N/A
***Branchipolynoe*** **Pettibone, 1984** [98]	***Branchipolynoe symmytilida*** **Pettibone, 1984** ** [98]	N/A
*Branchipolynoe*	*Branchinotogluma japonicus* (Miura & Hashimoto, 1991) [99]	*Branchipolynoe japonicus* comb. nov. (Miura & Hashimoto, 1991)
*Branchipolynoe*	*Branchinotogluma marianus* (Pettibone, 1989) [26]	*Branchipolynoe marianus* comb. nov. (Pettibone, 1989)
*Branchipolynoe*	*Branchinotogluma nanhaiensis* Wu, Zhen, Kou & Xu, 2023 [6]	*Branchipolynoe nanhaiensis* comb. nov. (Wu, Zhen, Kou & Xu, 2023)
*Branchipolynoe*	*Branchinotogluma nikkoensis* Jimi, Chen & Fujiwara, 2022 [5]	*Branchipolynoe nikkoensis* comb. nov. (Jimi, Chen & Fujiwara, 2022)
*Branchipolynoe*	*Branchinotogluma segonzaci* (Miura & Desbruyères, 1995) [28]	*Branchipolynoe segonzaci* comb. nov. (Miura & Desbruyères, 1995)
*Branchipolynoe*	*Branchinotogluma trifurcus* (Miura & Desbruyères, 1995) [28]	*Branchipolynoe trifurcus* comb. nov. (Miura & Desbruyères, 1995)
*Branchipolynoe*	*Branchipolynoe eliseae* Lindgren, Hatch, Hourdez, Seid & Rouse, 2019 [100]	N/A
*Branchipolynoe*	*Branchipolynoe halliseyae* Lindgren, Hatch, Hourdez, Seid & Rouse, 2019 [100]	N/A
*Branchipolynoe*	*Branchipolynoe kajsae* Lindgren, Hatch, Hourdez, Seid & Rouse, 2019 [100]	N/A
*Branchipolynoe*	*Branchipolynoe longqiensis* Zhou, Zhang, Lu & Wang, 2017 [101]	N/A
*Branchipolynoe*	*Branchipolynoe meridae* Lindgren, Hatch, Hourdez, Seid & Rouse, 2019 [100]	N/A
*Branchipolynoe*	*Branchipolynoe onnuriensis* Kim, Choi, Eyun, Kim & Yu, 2022 [102]	N/A
*Branchipolynoe*	*Branchipolynoe pettiboneae* Miura & Hashimoto, 1991 [99]	N/A
*Branchipolynoe*	*Branchipolynoe seepensis* Pettibone, 1986 [103]	N/A
*Branchipolynoe*	*Branchipolynoe tjiasmantoi* Lindgren, Hatch, Hourdez, Seid & Rouse, 2019 [100]	N/A
***Cladopolynoe*** **Hiley & Rouse, gen. nov.**	***Branchinotogluma tunnicliffeae*** (Pettibone, 1988) ** [29]	***Cladopolynoe tunnicliffeae* comb. nov.** (Pettibone, 1988) **
*Cladopolynoe*	*Branchinotogluma jiaolongae* Han, Zhou, Chen & Wang, 2023 [10]	*Cladopolynoe jiaolongae* comb. nov. (Han, Zhou, Chen & Wang, 2023)
*Cladopolynoe*	*Branchinotogluma sandersi* Pettibone, 1985 [27]	*Cladopolynoe sandersi* comb. nov. (Pettibone, 1985)
***Lepidonotopodium*** **Pettibone, 1983** [33]	***Lepidonotopodium fimbriatum*** Pettibone, 1983 ** [33]	N/A
*Lepidonotopodium*	*Levensteiniella plicata* Hourdez & Desbruyères, 2000 [36]	*Lepidonotopodium plicata* comb. nov. (Hourdez & Desbruyères, 2000)
***Levensteiniella*** **Pettibone, 1985** [35]	***Levensteiniella kincaidi*** Pettibone, 1985 ** [35]	N/A
*Levensteiniella*	*Lepidonotopodium minutum* Pettibone, 1989 -- [26]	*Levensteiniella minutum * comb. nov. (Pettibone, 1989) --
*Levensteiniella*	*Lepidonotopodium okinawae* Sui & Li, 2017 [104]	*Levensteiniella okinawae* comb. nov. (Sui & Li, 2017)
*Levensteiniella*	*Lepidonotopodium riftense* Pettibone, 1984 -- [32]	*Levensteiniella riftense * comb. nov. (Pettibone, 1984) --
***Mamiwata*** **Hiley & Rouse, gen. nov.**	***Lepidonotopodium piscesae*** Pettibone, 1988 ** [29]	***Mamiwata piscesae* comb. nov.** (Pettibone, 1988) **
*Mamiwata*	*Lepidonotopodium jouinae* Desbruyères & Hourdez, 2000 -- [105]	*Mamiwata jouinae * comb. nov. (Desbruyères & Hourdez, 2000) --
*Mamiwata*	*Lepidonotopodium williamsae* Pettibone, 1984 [32]	*Mamiwata williamsae* comb. nov. (Pettibone, 1984)
***Photinopolynoe*** **Hiley & Rouse, gen. nov.**	***Branchinotogluma ovata*** Wu, Zhan & Xu, 2019 ** [9]	***Photinopolynoe ovata* comb. nov.** (Wu, Zhan & Xu, 2019) **
*Photinopolynoe*	*Branchinotogluma burkensis* Pettibone, 1989 -- [26]	*Photinopolynoe burkensis * comb. nov. (Pettibone, 1989) --
*Photinopolynoe*	*Branchinotogluma elytropapillata* Zhang, Chen & Qiu, 2018 [8]	*Photinopolynoe elytropapillata* comb. nov. (Zhang, Chen & Qiu, 2018)
*Photinopolynoe*	*Branchinotogluma kaireiensis* Han, Zhou, Chen & Wang, 2023 [10]	*Photinopolynoe kaireiensis* comb. nov. (Han, Zhou, Chen & Wang, 2023)
*Photinopolynoe*	*Branchinotogluma pettiboneae* Wu, Zhan & Xu, 2019 [9]	*Photinopolynoe pettiboneae* comb. nov. (Wu, Zhan & Xu, 2019)
*Photinopolynoe*	*Branchinotogluma robusta* Wu, Zhen, Kou & Xu, 2023 [6]	*Photinopolynoe robusta* comb. nov. (Wu, Zhen, Kou & Xu, 2023)
*Photinopolynoe*	*Branchinotogluma sagamiensis* Jimi, Chen & Fujiwara, 2022 [5]	*Photinopolynoe sagamiensis* comb. nov. (Jimi, Chen & Fujiwara, 2022)
***Stratigos*** **Hiley & Rouse, gen. nov.**	***Branchinotogluma bipapillata*** Zhou, Wang, Zhang & Wang, 2018 ** [7]	***Stratigos bipapillata*** **comb. nov.** (Zhou, Wang, Zhang & Wang, 2018) **
***Themis*** **Hiley & Rouse, gen. nov.**	***Levensteiniella intermedia*** Pettibone, 1990 ** [34]	***Themis intermedia*** **comb. nov.** (Pettibone, 1990) **
*Themis*	*Lepidonotopodium atalantae* Desbruyères & Hourdez, 2000 -- [106]	*Themis atalantae * comb. nov. (Desbruyères & Hourdez, 2000) --
*Themis*	*Levensteiniella iris* Hourdez & Desbruyères, 2003 -- [107]	*Themis iris * comb. nov. (Hourdez & Desbruyères, 2003) --
*Themis*	*Levensteiniella longqiensis* Han, Zhou, Chen & Wang, 2023 [10]	*Themis longqiensis* comb. nov. (Han, Zhou, Chen & Wang, 2023)
*Themis*	*Levensteiniella manusensis* Wu & Xu, 2018 [108]	*Themis manusensis* comb. nov. (Wu & Xu, 2018)
*Themis*	*Levensteiniella pettiboneae* Han, Zhou, Chen & Wang, 2023 [10]	*Themis pettiboneae* comb. nov. (Han, Zhou, Chen & Wang, 2023)
*Themis*	*Levensteiniella raisae* Pettibone, 1989 -- [26]	*Themis raisae * comb. nov. (Pettibone, 1989) --
*Themis*	*Levensteiniella undomarginata* Zhang, Chen & Qiu, 2018 [8]	*Themis undomarginata* comb. nov. (Zhang, Chen & Qiu, 2018)

***Branchinotogluma* Pettibone, 1985 [27], emended**

**Type species:** *Branchinotogluma hessleri* Pettibone, 1985 [27].

**Diagnosis (emended):** Lepidonotopodini with 21 segments. Ten pairs of elytra. Elytra round to oval, large covering dorsum, and delicate with branched veins. Sexual dimorphism. Males possessing compressed posterior four segments (18–21) with greatly modified parapodia differing from one another [27]; parapodia on segment 18 smaller than on remaining segments; parapodia on segment 19 lack notochaetae and neurochaetae; parapodia on segment 20 possess wheel organs (neuropodia flared and flattened distally with radiating neurochaetae in some cases); notopodia on segment 21 with expanded lamellae, thickened acicular lobes fused to cirrophores of dorsal cirri, without notochaetae. Males have notopodia with two groups of branchiae on segments 3–16 and a single group of branchiae on segment 17. Females without modified parapodia; notopodia with two groups of branchiae on segments 3–21. Bilobed prostomium. Males have one pair of very long (extend posteriorly to circa segment 15) ventral papillae on segment 12 followed by five pairs of short and rounded ventral lamellae on segments 13–17. Females without ventral papillae but with six pairs of rounded ventral lamellae on segments 11–16. Pharynx with three dorsal and two ventral border papillae. Two pairs of dorsal and ventral jaws, minute denticles on inner border.

**Remarks:** *Branchinotogluma* is currently a monotypic genus containing the type species *B. hessleri*, which has available DNA data. Apomorphic features for *Branchinotogluma* are six pairs of ventral lamellae on segments 11–16 in females and wheel organs on modified parapodia of segment 20 in males.

***Branchipolynoe* Pettibone, 1984 [98], emended**

**Type species:** *Branchipolynoe symmytilida* Pettibone, 1984 [98].

**Diagnosis (emended):** Free-living or symbiotic species. Lepidonotopodini with 20–21 segments. Ten pairs of elytra, greatly reduced on segments 17 and/or 19 in some cases. Elytra round, oval, or subreniform. Elytra: large covering dorsum, moderately large/medium leaving middle section of dorsum uncovered, or small leaving large portion of dorsum uncovered. Elytra: smooth, thick, opaque, lack micro- and macrotubercles and papillae, without border papillae, with branched veins in some cases. Sexual dimorphism, with males possessing modified parapodia on segments 20–21, 18–21, or 15–21, or without modifications, and females without modified parapodia. Notopodia with arborescent branchiae in two groups, starting on segment 2 or 3. Branchiae either continue to segment 15 with a single group present, 16 with a single group present, 18, 19, or 21, and are progressively reduced in posterior end. Nearly unilobed or bilobed prostomium. Males have five pairs of semi-ovate (or squarish) flat ventral lamellae on segments 13–17, six pairs of short and flat ventral lamellae on segments 13–18, or are without ventral lamellae. Females lack ventral lamellae. In males, one pair of ventral papillae on segment 12 (ranging from short to very long). In females, two pairs of ventral papillae on segments 11–12, one pair of short and squarish ventral papillae on segment 11, or without ventral papillae. Pharynx with five dorsal and five ventral border papillae, four dorsal and four ventral border papillae, or six dorsal and six ventral border papillae. In some cases, two clusters of small papillae on lateral sides of pharynx (each with 4–5 papillae) and two processes at lateral bases, and/or numerous small papillae forming a proximal band. Two pairs of dorsal and ventral jaws without teeth (smooth) or with small teeth (minute denticles). All *Branchipolynoe* species with minute denticles have 21 segments.

**Remarks:** *Branchipolynoe* is expanded to include the six previously described free-living *Branchinotogluma* species: *B. japonicus*, *B. marianus*, *B. nanhaiensis*, *B. nikkoensis*, *B. segonzaci*, and *B. trifurcus* (=*Branchipolynoe japonicus* comb. nov., *Branchipolynoe marianus* comb. nov., *Branchipolynoe nanhaiensis* comb. nov., *Branchipolynoe nikkoensis* comb. nov., *Branchipolynoe segonzaci* comb. nov., and *Branchipolynoe trifurcus* comb. nov.), increasing the total number of known species within *Branchipolynoe* to 16. *Branchipolynoe* also includes two undescribed species used in the analyses here: *Branchinotogluma* cf. *marianus* and *Branchinotogluma* cf. *segonzaci* (=*Branchipolynoe* cf. *marianus* and *Branchipolynoe* cf. *segonzaci*).

Although there are currently no obvious apomorphic features for *Branchipolynoe*, the combination of diagnostic features is sufficient to identify *Branchipolynoe* species. *Branchipolynoe* shares the most diagnostic features with *Photinopolynoe* gen. nov. amongst the Lepidonotopodini branchiate genera. However, *Branchipolynoe* is distinguished from *Photinopolynoe* gen. nov. in that its species have either smooth jaws or a combination of minutely denticled jaws with 21 segments (Table S8). *Photinopolynoe* gen. nov. species primarily have jaws with numerous distinct teeth (often ≥ 30) or a combination of minutely denticled jaws with 20 segments (solely in *Photinopolynoe sagamiensis* comb. nov.) (Table S8).

***Cladopolynoe* Hiley & Rouse, gen. nov.**

**Type species:** *Cladopolynoe tunnicliffeae* comb. nov. (Pettibone, 1988) [29].

**Diagnosis:** Lepidonotopodini with 21 segments. Ten pairs of elytra. Elytra round to oval, smooth. Elytra stiff and opaque, iridescent when fresh. Sexual dimorphism, with males possessing modified parapodia on segments 18–21 or 19–21, and parapodia not modified in females. Notopodia with arborescent branchiae in two groups, starting on segment 3 and continuing to either segment 18 or posterior end. In some cases, single group of branchiae on segment 3. Bilobed prostomium. Females with five pairs of small, squarish (or round) ventral papillae on segments 11–15, ventral lamellae absent. Males with four pairs of long ventral papillae on segments 12–15 and either two pairs of rounded ventral lamellae on segments 16–17 or three pairs on segments 16–18. Pharynx with three dorsal border papillae and two ventral border papillae. Two pairs of dorsal and ventral jaws, each with numerous small teeth (minute denticles) on inner border.

**Remarks:** *Cladopolynoe* gen. nov. includes three already known *Branchinotogluma* species with available DNA data: *B. tunnicliffeae*, *B. jiaolongae*, and *B. sandersi* (=*Cladopolynoe tunnicliffeae* comb. nov. (type species), *Cladopolynoe jiaolongae* comb. nov., and *Cladopolynoe sandersi* comb. nov.). *Cladopolynoe* gen. nov. also includes two undescribed species used in the analyses here: *Branchinotogluma* cf. *sandersi* and *Branchinotogluma* sp. nov. 4 (=*Cladopolynoe* cf. *sandersi* and *Cladopolynoe* sp. nov. 4 (description *in prep*, Hiley et al. [109])).

*Cladopolynoe* gen. nov. shares diagnostic traits with other Lepidonotopodini branchiate genera (e.g., five pairs of ventral papillae on segments 11–15 in females for *Cladopolynoe* gen. nov., *Photinopolynoe* gen. nov. in some cases, and *Stratigos* gen. nov.; four pairs of ventral papillae on segments 12–15 in males for *Cladopolynoe* gen. nov. and *Photinopolynoe* gen. nov. in some cases; pharynx with three dorsal and two ventral border papillae in *Branchinotogluma*, *Cladopolynoe* gen. nov., and *Photinopolynoe* gen. nov. in some cases).

Although there are currently no obvious apomorphic features for *Cladopolynoe* gen. nov., the combination of diagnostic features is sufficient to identify *Cladopolynoe* gen. nov. species. Amongst the Lepidonotopodini branchiate genera, *Cladopolynoe* gen. nov. shares the most diagnostic features with *Photinopolynoe* gen. nov. and *Stratigos* gen. nov., but is distinguished from them as follows: *Cladopolynoe* gen. nov. has a combination of minutely denticled jaws with 21 segments, while the only *Photinopolynoe* gen. nov. species with minutely denticled jaws has 20 segments (all other *Photinopolynoe* gen. nov. species have jaws with numerous distinct teeth) (see Tables S7 and S8). Furthermore, *Cladopolynoe* gen. nov. is distinguished from *Stratigos* gen. nov. in that its species have three dorsal and two ventral border papillae surrounding the pharynx, as opposed to five dorsal and five ventral border papillae (Table S8).

**Etymology:** *Cladopolynoe* gen. nov. is named after the Greek word κλάδος (klados), which translates to branch, because of the arborescent or tree-branching branchiae in this genus.

***Lepidonotopodium* Pettibone, 1983** [33], **emended**

**Type species:** *Lepidonotopodium fimbriatum* Pettibone, 1983 [33].

**Diagnosis (emended):** Lepidonotopodini with 28 segments. Eleven pairs of elytra. Thick elytra, leathery or smooth, overlapping and covering dorsum except first and last pairs or leaving the middle part of dorsum uncovered. Each elytron has two raised smooth macrotubercles on the posterior third or a prominent longitudinal fold in the middle. Elytral surface sometimes covered with numerous round microtubercles and some scattered globular micropapillae. Parapodia not modified on posterior segments. Notopodia without branchiae. Bilobed prostomium. Ventral lamellae absent. Sexual dimorphism, with males possessing two pairs of long ventral papillae on segments 11–12 and females lacking ventral papillae. Pharynx with seven dorsal border papillae and seven ventral border papillae; in some cases, middle papilla on dorsal side is larger than the others and middle papilla on ventral side is smaller than the others. Two pairs of hooked or straight dorsal and ventral jaws, sometimes fused medially. Jaws with either five to seven teeth on the base, or up to twenty teeth increasing in size from the tip to the base.

**Remarks:** *Lepidonotopodium* includes two species, and DNA data are available for both *Lepidonotopodium fimbriatum* (type species) and *Levensteiniella plicata (=Lepidonotopodium plicata* comb. nov.).

*Lepidonotopodium* is unique in having elytra with two raised macrotubercles on the posterior third (*Lepidonotopodium fimbriatum*) or with a longitudinal fold in the middle (*Lepidonotopodium plicata* comb. nov.), both of which are absent in other Lepidonotopodini genera. The presence of either elytral morphology, along with the combination of the remaining diagnostic traits, can be used to identify *Lepidonotopodium* specimens.

***Levensteiniella* Pettibone, 1985 [35], emended**

**Type species:** *Levensteiniella kincaidi* Pettibone, 1985 [35].

**Diagnosis (emended):** Lepidonotopodini with 22–25 segments. Eleven pairs of elytra. Elytra round, oval, or subreniform shape. Elytra large and mainly overlapping, covering dorsum. Elytra with branching veins, opaque. Scattered micropapillae near the posterior and lateral borders and on the surface of elytra. In some cases, numerous scattered foveolae near the posterior and lateral borders of elytra with micropapillae inside. Parapodia not modified on posterior segments. Notopodia without branchiae. Bilobed prostomium. Ventral lamellae absent. Sexual dimorphism, with males possessing two pairs of long ventral papillae on segments 11–12 or three pairs of long ventral papillae on segments 11–13, and females lacking ventral papillae. Pharynx with seven dorsal border papillae and seven ventral border papillae; middle papillae are larger in some cases. Alternatively, pharynx with nine dorsal border papillae and nine ventral border papillae. Two pairs of dorsal and ventral jaws without teeth or serrated with numerous small teeth (minute denticles).

**Remarks:** *Levensteiniella* includes two species, and DNA data are available for both *Levensteiniella kincaidi* (type species) and *Lepidonotopodium okinawae* (=*Levensteiniella okinawae* comb. nov.). *Levensteiniella* also includes the undescribed species *Lepidonotopodium* cf. *riftense* (=*Levensteiniella* cf. *riftense*) used in the analyses here. Due to the placement of *Lepidonotopodium* cf. *riftense* within *Levensteiniella* (Figure 1, Figure 2, Figure A1, Figure A2, Figure A3 and Figure A4), we assign *Lepidonotopodium riftense* to *Levensteiniella incertae sedis* (Table 2); DNA data from *L. riftense* specimens collected at the type locality is needed to confirm its placement.

Although there are presently no obvious apomorphic features for *Levensteiniella*, the combination of diagnostic features is sufficient to identify the *Levensteiniella* species. *Levensteiniella* shares many diagnostic features with *Themis* gen. nov. (e.g., similar segment range; males with two pairs of long ventral papillae on segments 11–12 in some species; pharynx with seven dorsal and seven ventral border papillae in some species). However, *Themis* gen. nov. only has jaws without distinct teeth, while *Levensteiniella* has jaws with (*Levensteiniella okinawae* comb. nov.) or without teeth (*Levensteiniella kincaidi*) (Tables S7 and S8). Also, *Themis* gen. nov. has elytra with obvious macrotubercles along the posterior border (often forming a defined wave-shaped edge), apart from *Levensteiniella pettiboneae* (=*Themis pettiboneae* comb. nov.), which has micropapillae along the posterior border, while *Levensteiniella* has elytra with scattered micropapillae on the surface majority and near the posterior/lateral borders (Tables S7 and S8). Thus, the difference in elytral morphology distinguishes *Levensteiniella* from *Themis* gen. nov.

*Lepidonotopodium minutum* is assigned to *Levensteiniella incertae sedis* (Table 2) based on morphology (Table S7), specifically the presence of 23 segments and posterior half of elytra covered with scattered micropapillae; DNA data from *L. minutum* specimens collected at the type locality is needed to confirm placement.

***Mamiwata* Hiley & Rouse, gen. nov.**

**Type species:** *Mamiwata piscesae* comb. nov. (Pettibone, 1988) [29].

**Diagnosis:** Lepidonotopodini with 24–28 segments. Eleven pairs of elytra. Elytra are oval to subreniform, large and overlapping, covering dorsum, thick, stiff, opaque, without raised macrotubercles. Elytral surface nearly covered with rounded to conical microtubercles and scattered globular micropapillae. Parapodia not modified on posterior segments. Notopodia without branchiae. Bilobed prostomium. Ventral lamellae absent. Sexual dimorphism, with males possessing five pairs of long ventral papillae on segments 11–15 or four pairs of long ventral papillae on segments 12–15, and females lacking ventral papillae. Pharynx with seven dorsal border papillae and seven ventral border papillae; middle papilla on dorsal side is larger than the others in some cases. Two pairs of dorsal and ventral jaws with well-defined teeth (either from five to seven teeth or circa twelve teeth).

**Remarks:** *Mamiwata* gen. nov. includes two previously described *Lepidonotopodium* species containing DNA data: *L. piscesae* and *L. williamsae* (=*Mamiwata piscesae* comb. nov. (type species) and *Mamiwata williamsae* comb. nov.). *Mamiwata* gen. nov. also includes the undescribed species *Lepidonotopodium* cf. *williamsae* (=*Mamiwata* cf. *williamsae*) used in the analyses here.

*Mamiwata* gen. nov. is unique amongst the non-branchiate Lepidonotopodini genera in having males with five pairs of long ventral papillae on segments 11–15 (*Mamiwata piscesae* comb. nov.) or four pairs of long ventral papillae on segments 12–15 (*Mamiwata williamsae* comb. nov.). The combination of notopodia without branchiae and either of these male morphologies allows for the identification of *Mamiwata* gen. nov. males. Although *Mamiwata* gen. nov. females are similar to *Lepidonotopodium* and *Levensteiniella* females, the combination of diagnostic features allows for the identification of *Mamiwata* gen. nov. females. Importantly, *Mamiwata* gen. nov. is distinguished from *Lepidonotopodium* in having different elytral morphology, specifically in lacking two raised macrotubercles or a longitudinal fold on the elytra as in *Lepidonotopodium* (Table S8). Furthermore, *Mamiwata* gen. nov. is distinguished from *Levensteiniella* in having jaws with well-defined teeth, as opposed to jaws without teeth or with numerous serrated teeth (minute denticles) as in *Levensteiniella* (Table S8).

*Lepidonotopodium jouinae* is assigned to *Mamiwata incertae sedis* (Table 2) based on morphology (Table S7), specifically the presence of five pairs of long ventral papillae on segments 11–15 in males and branchiae absent on notopodia, along with the combination of remaining diagnostic features present; DNA data from *L. jouinae* specimens collected at the type locality is needed to confirm placement.

**Etymology:** *Mamiwata* gen. nov. is named after the mermaid-like African water spirit Mami Wata, who is worshiped by many today.

***Photinopolynoe* Hiley & Rouse, gen. nov.**

**Type species:** *Photinopolynoe ovata* comb. nov. (Wu, Zhan & Xu, 2019) [9].

**Diagnosis:** Lepidonotopodini with 20–21 segments. Ten pairs of elytra, reduced on segment 19 in some cases. Elytra round, oval, or subreniform shape, overlapping and covering dorsum. Elytra either smooth, delicate, without tubercles or papillae on surface, with or without numerous tiny globular papillae along posterior and lateral margins, or with numerous small papillae along non-overlapping margin and numerous globular papillae on non-overlapping area of elytral surface. Sexual dimorphism, with males possessing modified parapodia on segments 18–20, 19–21, 20–21, or without modified parapodia; parapodia not modified in females. Notopodia with arborescent branchiae in two groups, starting on segment 3. In males, branchiae continue to segment 16, 17, or 19, with branchiae reduced on posterior segments in most cases. In females, branchiae continue to segment 20 (with a single group present), 16, 18, or 21 (with branchiae greatly reduced on posterior segments, to a small papilla in some cases). Bilobed prostomium. Females lacking ventral lamellae. Males with five pairs of thin and semi-ovate ventral lamellae on segments 13–17; six pairs on segments 13–18; or two pairs of short, rounded ventral lamellae on segments 16–17. Females lacking ventral papillae; with five pairs of short, rounded ventral papillae on segments 11–15; or with seven short pairs of ventral papillae on segments 11–17. Males with one pair of ventral papillae (short or long) on segment 12 or four pairs on segments 12–15. Pharynx with four dorsal and four ventral border papillae; with five dorsal and four ventral border papillae; or with three dorsal and two ventral border papillae; unequal size of border papillae in some cases. Some species have two clusters of papillae (or rounded processes) at the lateral bases of the pharynx. Two pairs of dorsal and ventral jaws, primarily with numerous distinct teeth (often ≥ 30) or with minute denticles on the inner border. *Photinopolynoe sagamiensis* comb. nov., the only species with minute denticles as opposed to distinct teeth, has 20 segments.

**Remarks:** *Photinopolynoe* gen. nov. includes six already known *Branchinotogluma* species with available DNA data: *B. ovata*, *B. elytropapillata*, *B. kaireiensis*, *B. pettiboneae*, *B. robusta*, and *B. sagamiensis* (=*Photinopolynoe ovata* comb. nov. (type species), *Photinopolynoe elytropapillata* comb. nov., *Photinopolynoe kaireiensis* comb. nov., *Photinopolynoe pettiboneae* comb. nov., *Photinopolynoe robusta* comb. nov., and *Photinopolynoe sagamiensis* comb. nov.). *Photinopolynoe* gen. nov. also includes four undescribed species used in the analyses here: *Branchinotogluma* sp. nov. 2–3 and *Branchinotogluma* sp. nov. 5–6 (=*Photinopolynoe* sp. nov. 2–3 and *Photinopolynoe* sp. nov. 5–6, descriptions *in prep*, Hiley et al.).

*Photinopolynoe* gen. nov. have females without ventral papillae (solely in *P. sagamiensis* comb. nov.) or with either five or seven pairs of short ventral papillae on segments 11–15 or 11–17. The latter is an apomorphy for the smaller *Photinopolynoe* gen. nov. clade containing *P. kaireiensis* comb. nov., *P. pettiboneae* comb. nov., and *P. robusta* comb. nov. (see Figure 1 and Figure 2). However, females may also have five pairs of short ventral papillae on segments 11–15, which is a trait shared with *Cladopolynoe* gen. nov. and *Stratigos* gen. nov.

Although there are presently no obvious apomorphic features for *Photinopolynoe* gen. nov., the combination of diagnostic features is sufficient to identify *Photinopolynoe* gen. nov. species (see *Branchipolynoe* and *Cladopolynoe* gen. nov. remarks). Furthermore, *Photinopolynoe* gen. nov. is distinguished from *Stratigos* gen. nov. in having different numbers of border papillae surrounding the pharynx; *Photinopolynoe* gen. nov. has four dorsal and four ventral, five dorsal and four ventral, or three dorsal and two ventral border papillae, while *Stratigos* gen. nov. has five dorsal and five ventral border papillae (Table S8).

*Branchinotogluma burkensis* is assigned to *Photinopolynoe incertae sedis* (Table 2) based on morphology (Table S7), specifically the presence of seven pairs of short ventral papillae on segments 11–17 in females, along with the combination of remaining diagnostic features present; DNA data from *B. burkensis* specimens collected at the type locality is needed to confirm placement.

**Etymology:** *Photinopolynoe* gen. nov. is named after the Greek word φωτεινός (phóteinos), which translates to bright or luminous, because of the beautiful iridescent elytra in this genus.

***Stratigos* Hiley & Rouse, gen. nov.**

**Type species:** *Stratigos bipapillata* comb. nov. (Zhou, Wang, Zhang & Wang, 2018) [7].

**Diagnosis:** Lepidonotopodini with 21 segments. Ten pairs of elytra, reduced on segment 19 in some cases. Elytra oval or subreniform, thick, smooth. Sexual dimorphism, with males possessing modified parapodia on segments 18–21 and parapodia not modified in females. Notopodia with arborescent branchiae in two groups, starting on segment 3. In females, branchiae continue to segment 19 with a single group present. In males, branchiae continue to segment 18 with a single group present. Bilobed prostomium. In females, ventral lamellae absent. In males, four pairs of rounded ventral lamellae on segments 14–17. Females have five pairs of short, rounded ventral papillae on segments 11–15. Males have two long pairs of ventral papillae on segments 12–13. Pharynx with five dorsal and five ventral border papillae. Two pairs of dorsal and ventral hooked jaws, minutely denticled on inner borders.

**Remarks:** *Stratigos* gen. nov. is currently a monotypic genus containing the type species *Stratigos bipapillata* comb. nov., which has available DNA data. *Stratigos* gen. nov. also includes the undescribed species *Branchinotogluma* sp. nov. 1 (=*Stratigos* sp. nov. 1, description *in prep*, Hiley et al.) used in the analyses here.

Apomorphic features for *Stratigos* gen. nov. are two long pairs of ventral papillae on segments 12–13 followed by four pairs of rounded ventral lamellae on segments 14–17 in males (Tables S7 and S8). Furthermore, the combination of diagnostic features is sufficient to identify *Stratigos* gen. nov. species, including females (see *Cladopolynoe* gen. nov. and *Photinopolynoe* gen. nov. remarks).

**Etymology:** *Stratigos* gen. nov. is named after the Stratigos family, lifelong friends of the first author, in recognition of their continued faith and strength following the passing of their beloved son Theodore John Stratigos.

***Themis* Hiley & Rouse, gen. nov**.

**Type species:** *Themis intermedia* comb. nov. (Pettibone, 1990) [34].

**Diagnosis:** Lepidonotopodini with 23–28 segments. Eleven pairs of elytra. Elytra round, oval, or subreniform shape. Elytra large and mainly overlapping, covering dorsum. Elytra thick and stiff or smooth, opaque or translucent, with thick oval or bulbous raised areas or projections (macrotubercles) along posterior border, wave-shaped posterior edge in some species. Micropapillae and/or macropapillae on elytral surface, and micropapillae on posterior and lateral border in some cases. Parapodia not modified on posterior segments. Notopodia without branchiae. Bilobed prostomium. Ventral lamellae absent. Sexual dimorphism likely in all species. If present, males possess one pair of long ventral papillae on segment 11 or two pairs of long (or short and cylindrical, as in *Themis longqiensis* comb. nov.) ventral papillae on segments 11–12; ventral papillae absent in females. Sexual dimorphism not confirmed in *Themis intermedia* comb. nov., but it is likely that the holotype is male given that it possesses one pair of long ventral papillae on segment 11. Pharynx with seven dorsal border papillae and seven ventral border papillae; middle papillae are smaller in some cases. Pharynx sometimes has one pair of rounded papillae on lateral sides. Two pairs of dorsal and ventral jaws (mostly hooked) without distinct teeth.

**Remarks:** *Themis* gen. nov. includes five already known *Levensteiniella* species with available DNA data: *L. intermedia*, *L. longqiensis*, *L. manusenesis*, *L. pettiboneae*, and *L. undomarginata* (*Themis intermedia* comb. nov. (type species), *Themis longqiensis* comb. nov., *Themis manusensis* comb. nov., *Themis pettiboneae* comb. nov., and *Themis undomarginata* comb. nov.). *Themis* gen. nov. also includes two undescribed species used in the analyses here: *Levensteiniella* cf. *iris* (=*Themis* cf. *iris*) and *Themis* sp. nov. (description *in prep*, Hiley et al.). Due to the placement of *Levensteiniella* cf. *iris* within *Themis* gen. nov. (Figure 1, Figure 2, Figure A1, Figure A2, Figure A3 and Figure A4), we assign *Levensteiniella iris* to *Themis incertae sedis* (Table 2); DNA data from *L. iris* specimens collected at the type locality is needed to confirm placement.

Although there are currently no obvious apomorphic features for *Themis* gen. nov., the combination of diagnostic features still supports placement in the genus. *Themis* gen. nov. shares many diagnostic features with *Lepidonotopodium* and Levensteiniella (e.g., similar segment range; eleven pairs of elytra; males with two pairs of long ventral papillae on segments 11–12 in some species; pharynx with seven dorsal and seven ventral border papillae in most species). However, elytral morphologies of *Lepidonotopodium*, *Levensteiniella*, and *Themis* gen. nov. differ from one another. Although all three genera have scattered micropapillae on the elytral surface, *Themis* gen. nov. is unique in having elytra with well-defined macrotubercles or wave-shaped projections along the posterior border in all species except *Themis pettiboneae* comb. nov., which alternatively has micropapillae along the posterior border (Tables S7 and S8).

*Lepidonotopodium atalantae* and *Levensteiniella raisae* are assigned to *Themis incertae sedis* (Table 2) based on morphology (Table S7), given the presence of 23 and 27 segments, respectively, and elytra with macrotubercles along the posterior border in both species (very well-defined in *L. atalantae*). DNA data from *L. atalantae* and *L. raisae* specimens collected at the corresponding type localities is needed to confirm placement.

**Etymology:** *Themis* gen. nov. is named after Themis Michailides, father of the first author, for loving her genuinely and encouraging her career as a scientist since early childhood. It should be noted that there is a red algae genus *Themis* N. Sánchez, A. Vergés, [C. Peteiro] J. Sutherland & J. Brodie, 2014, but the homonym is allowed under the International Code of Zoological Nomenclature.

### 3.3. Mitogenome Organization and Statistics

**Figure A5:**
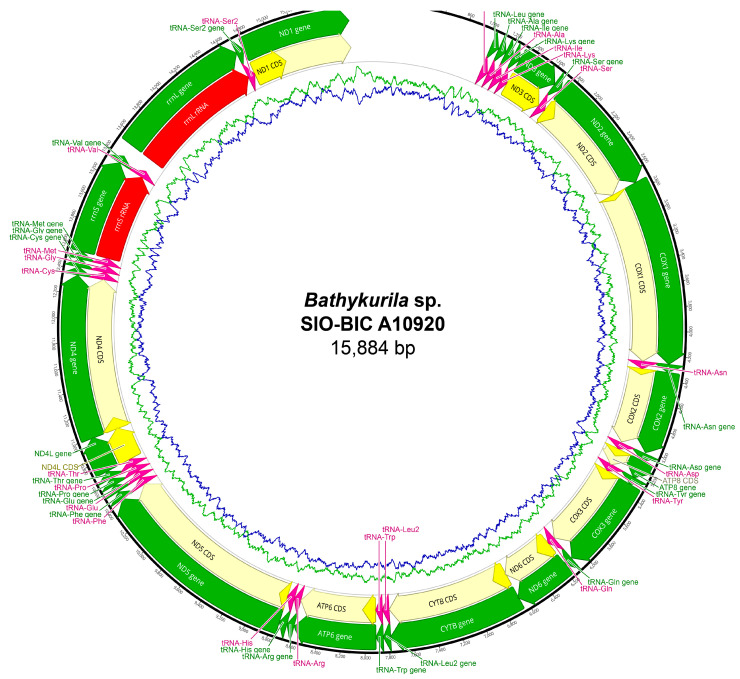
*Bathykurila* sp. (SIO-BIC A10920) complete mitogenome. The thick black circle represents the mitogenome sequence, with numbers indicating base pairs. Green arrows represent genes, yellow arrows represent protein coding sequences (CDS), red arrows represent ribosomal RNAs (rRNAs), and pink arrows represent transfer RNAs (tRNAs). The direction of the arrow represents the direction of the gene. If genes are long enough, their names and descriptions (gene, CDS, rRNA, or tRNA) are written on the corresponding arrows; otherwise, this information is written next to the gene and connected to it via green (gene), yellow (CDS), red (rRNA), or pink (tRNA) lines. The blue line graph plots the GC content and the green line graph plots the AT content of the mitogenome within a sliding window of 45 nucleotide base pairs (nt bp) at each individual nt bp position. Geneious Prime v. 2022.1.1 created by Biomatters was used to generate this figure. Available from https://www.geneious.com (accessed on 8 July 2024).

**Figure A6:**
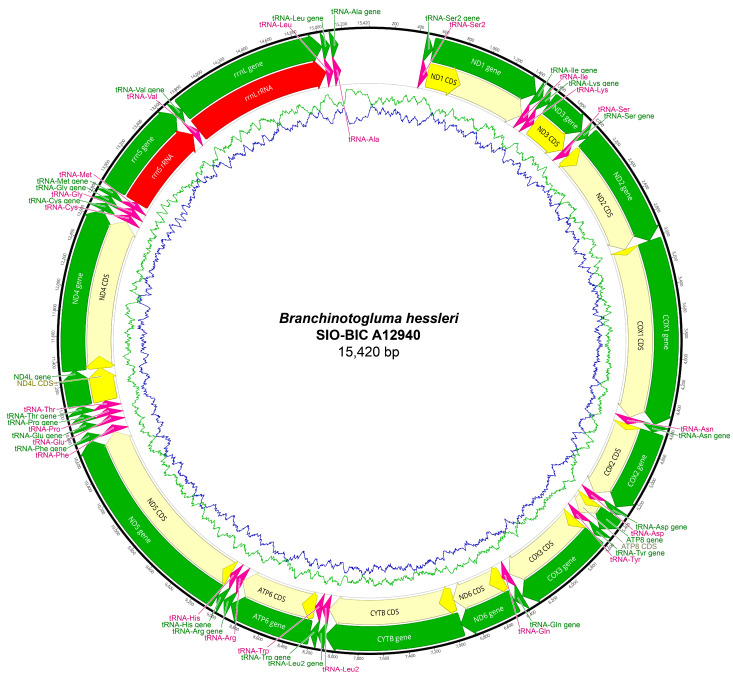
*Branchinotogluma hessleri* (SIO-BIC A12940) complete mitogenome. The thick black circle represents the mitogenome sequence, with numbers indicating base pairs. Green arrows represent genes, yellow arrows represent protein CDS, red arrows represent rRNAs, and pink arrows represent tRNAs. The direction of the arrow represents the direction of the gene. If genes are long enough, their names and descriptions (gene, CDS, rRNA, or tRNA) are written on the corresponding arrows; otherwise, this information is written next to the gene and connected to it via green (gene), yellow (CDS), red (rRNA), or pink (tRNA) lines. The blue line graph plots the GC content and the green line graph plots the AT content of the mitogenome within a sliding window of 45 nt bp at each individual nt bp position. Geneious Prime v. 2022.1.1 created by Biomatters was used to generate this figure. Available from https://www.geneious.com (accessed on 8 July 2024).

**Figure A7:**
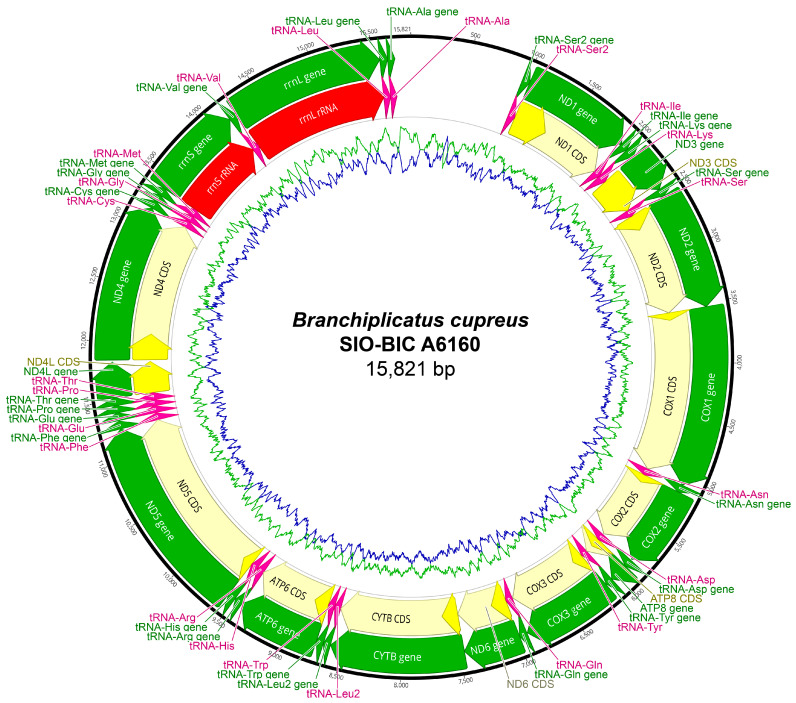
*Branchiplicatus cupreus* (SIO-BIC A6160) complete mitogenome. The thick black circle represents the mitogenome sequence, with numbers indicating base pairs. Green arrows represent genes, yellow arrows represent protein CDS, red arrows represent rRNAs, and pink arrows represent tRNAs. The direction of the arrow represents the direction of the gene. If genes are long enough, their names and descriptions (gene, CDS, rRNA, or tRNA) are written on the corresponding arrows; otherwise, this information is written next to the gene and connected to it via green (gene), yellow (CDS), red (rRNA), or pink (tRNA) lines. The blue line graph plots the GC content and the green line graph plots the AT content of the mitogenome within a sliding window of 45 nt bp at each individual nt bp position. Geneious Prime v. 2022.1.1 created by Biomatters was used to generate this figure. Available from https://www.geneious.com (accessed on 8 July 2024).

**Figure A8:**
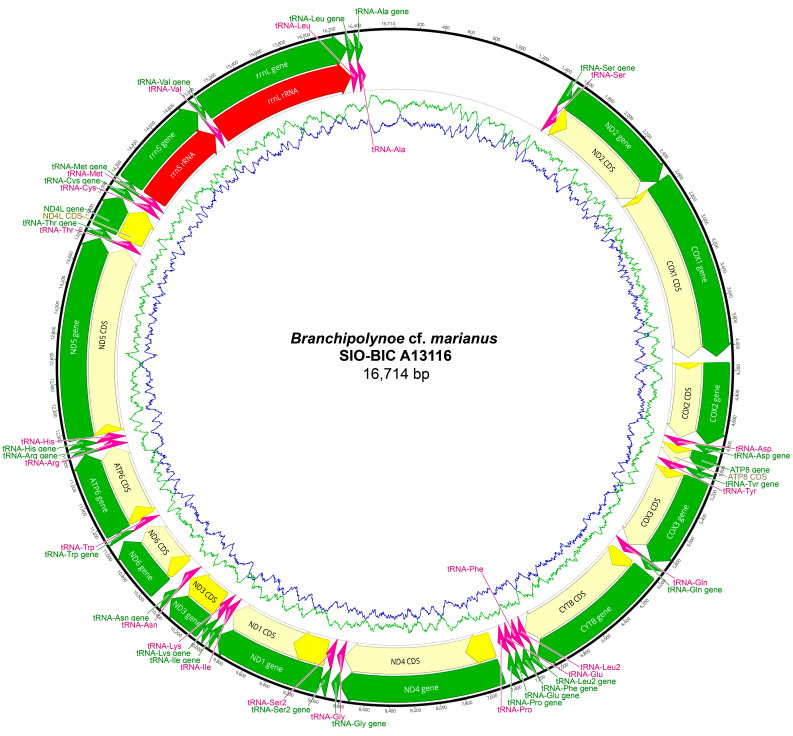
*Branchipolynoe* cf. *marianus* (SIO-BIC A13116) complete mitogenome. The thick black circle represents the mitogenome sequence, with numbers indicating base pairs. Green arrows represent genes, yellow arrows represent protein CDS, red arrows represent rRNAs, and pink arrows represent tRNAs. The direction of the arrow represents the direction of the gene. If genes are long enough, their names and descriptions (gene, CDS, rRNA, or tRNA) are written on the corresponding arrows; otherwise, this information is written next to the gene and connected to it via green (gene), yellow (CDS), red (rRNA), or pink (tRNA) lines. The blue line graph plots the GC content and the green line graph plots the AT content of the mitogenome within a sliding window of 45 nt bp at each individual nt bp position. Geneious Prime v. 2022.1.1 created by Biomatters was used to generate this figure. Available from https://www.geneious.com (accessed on 8 July 2024).

**Figure A9:**
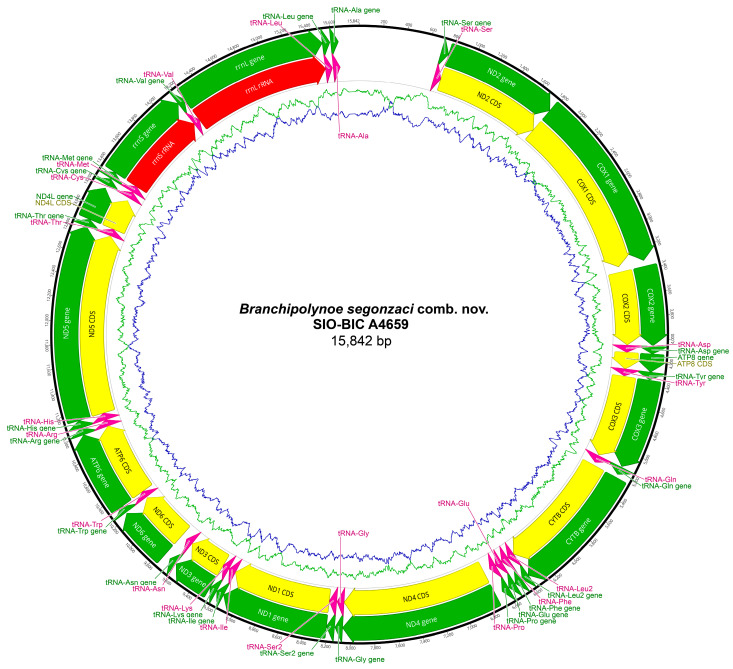
*Branchipolynoe segonzaci* comb. nov. (SIO-BIC A4659) complete mitogenome. The thick black circle represents the mitogenome sequence, with numbers indicating base pairs. Green arrows represent genes, yellow arrows represent protein CDS, red arrows represent rRNAs, and pink arrows represent tRNAs. The direction of the arrow represents the direction of the gene. If genes are long enough, their names and descriptions (gene, CDS, rRNA, or tRNA) are written on the corresponding arrows; otherwise, this information is written next to the gene and connected to it via green (gene), yellow (CDS), red (rRNA), or pink (tRNA) lines. The blue line graph plots the GC content and the green line graph plots the AT content of the mitogenome within a sliding window of 45 nt bp at each individual nt bp position. Geneious Prime v. 2022.1.1 created by Biomatters was used to generate this figure. Available from https://www.geneious.com (accessed on 8 July 2024).

**Figure A10:**
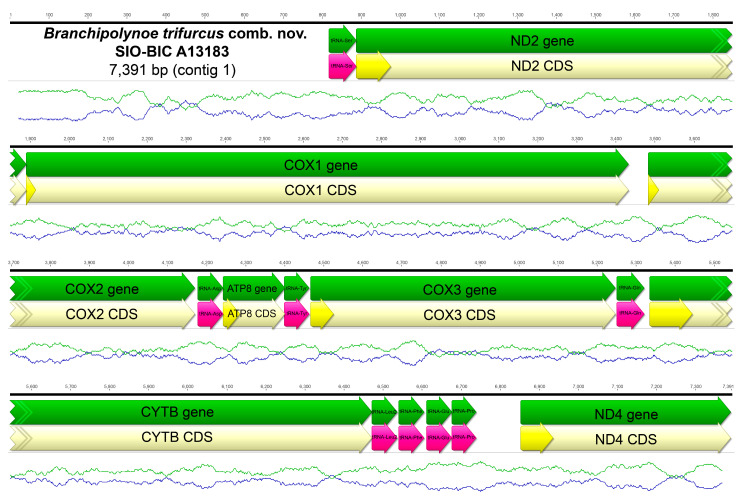
*Branchipolynoe trifurcus* comb. nov. (SIO-BIC A13183) incomplete mitogenome contig 1. The thick black line represents the partial mitogenome sequence, with numbers indicating base pairs. Green arrows represent genes, yellow arrows represent protein CDS, red arrows represent rRNAs, and pink arrows represent tRNAs. The direction of the arrow represents the direction of the gene. Gene names and descriptions (gene, CDS, rRNA, or tRNA) are written on the corresponding arrows. The blue line graph plots the GC content and the green line graph plots the AT content of the mitogenome within a sliding window of 45 nt bp at each individual nt bp position. Geneious Prime v. 2022.1.1 created by Biomatters was used to generate this figure. Available from https://www.geneious.com (accessed on 8 July 2024).

**Figure A11:**
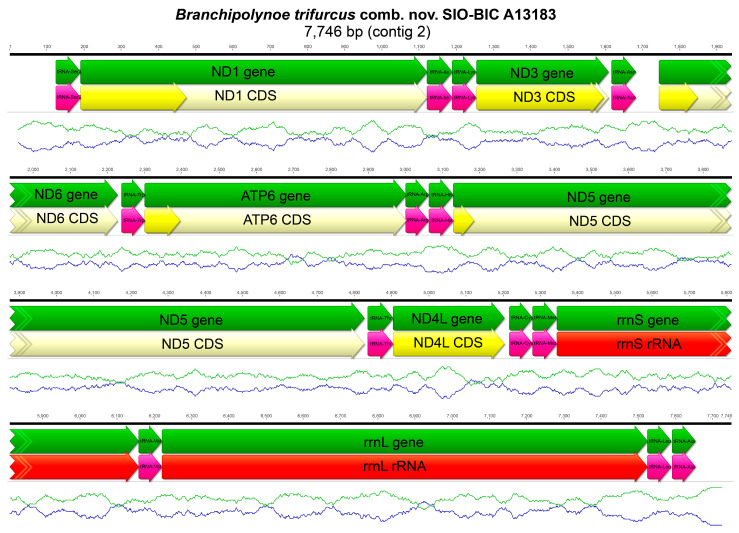
*Branchipolynoe trifurcus* comb. nov. (SIO-BIC A13183) incomplete mitogenome contig 2. The thick black line represents the partial mitogenome sequence, with numbers indicating base pairs. Green arrows represent genes, yellow arrows represent protein CDS, red arrows represent rRNAs, and pink arrows represent tRNAs. The direction of the arrow represents the direction of the gene. Gene names and descriptions (gene, CDS, rRNA, or tRNA) are written on the corresponding arrows. The blue line graph plots the GC content and the green line graph plots the AT content of the mitogenome within a sliding window of 45 nt bp at each individual nt bp position. Geneious Prime v. 2022.1.1 created by Biomatters was used to generate this figure. Available from https://www.geneious.com (accessed on 8 July 2024).

**Figure A12:**
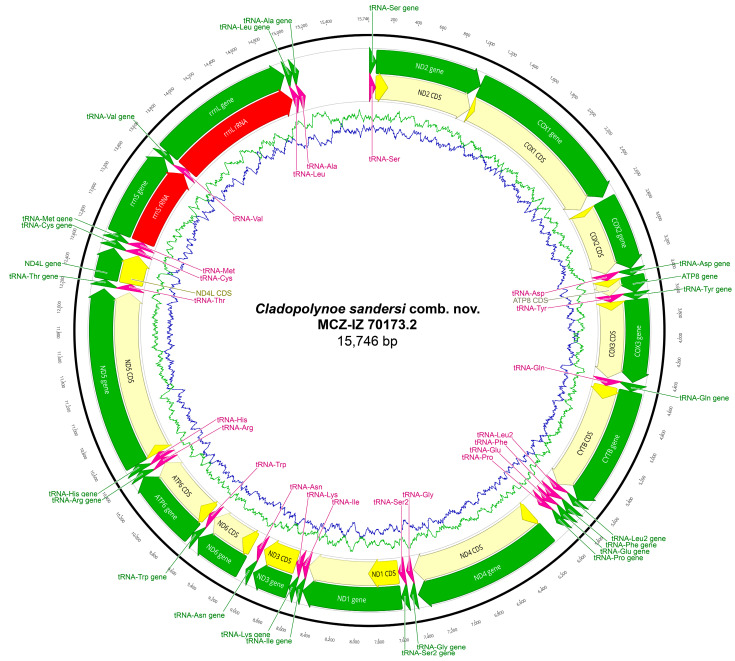
*Cladopolynoe sandersi* comb. nov. (MCZ-IZ 70173.2) complete mitogenome. The thick black circle represents the mitogenome sequence, with numbers indicating base pairs. Green arrows represent genes, yellow arrows represent protein CDS, red arrows represent rRNAs, and pink arrows represent tRNAs. The direction of the arrow represents the direction of the gene. If genes are long enough, their names and descriptions (gene, CDS, rRNA, or tRNA) are written on the corresponding arrows; otherwise, this information is written next to the gene and connected to it via green (gene), yellow (CDS), red (rRNA), or pink (tRNA) lines. The blue line graph plots the GC content and the green line graph plots the AT content of the mitogenome within a sliding window of 45 nt bp at each individual nt bp position. Geneious Prime v. 2022.1.1 created by Biomatters was used to generate this figure. Available from https://www.geneious.com (accessed on 8 July 2024).

**Figure A13:**
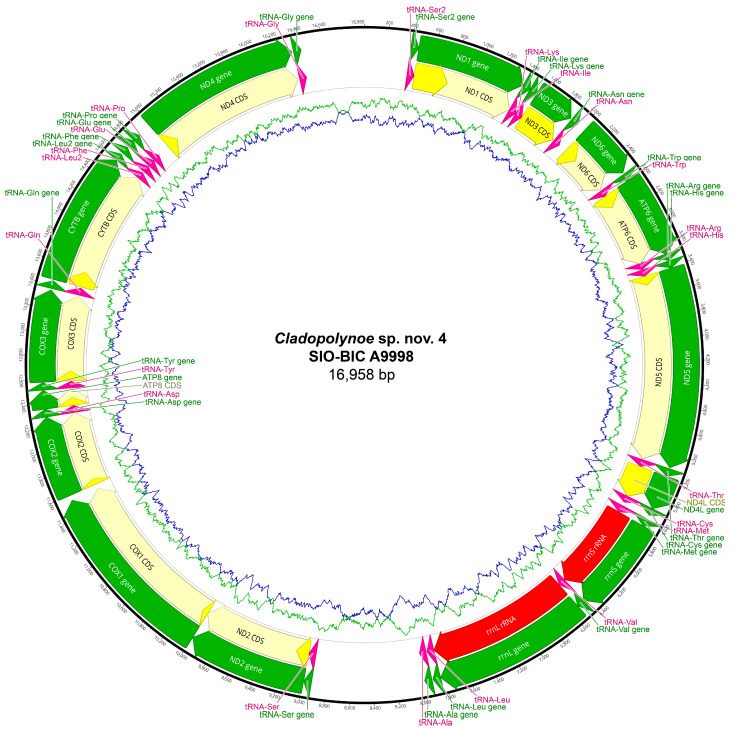
*Cladopolynoe* sp. nov. 4 (SIO-BIC A9998) complete mitogenome. The thick black circle represents the mitogenome sequence, with numbers indicating base pairs. Green arrows represent genes, yellow arrows represent protein CDS, red arrows represent rRNAs, and pink arrows represent tRNAs. The direction of the arrow represents the direction of the gene. If genes are long enough, their names and descriptions (gene, CDS, rRNA, or tRNA) are written on the corresponding arrows; otherwise, this information is written next to the gene and connected to it via green (gene), yellow (CDS), red (rRNA), or pink (tRNA) lines. The blue line graph plots the GC content and the green line graph plots the AT content of the mitogenome within a sliding window of 45 nt bp at each individual nt bp position. Geneious Prime v. 2022.1.1 created by Biomatters was used to generate this figure. Available from https://www.geneious.com (accessed on 8 July 2024).

**Figure A14:**
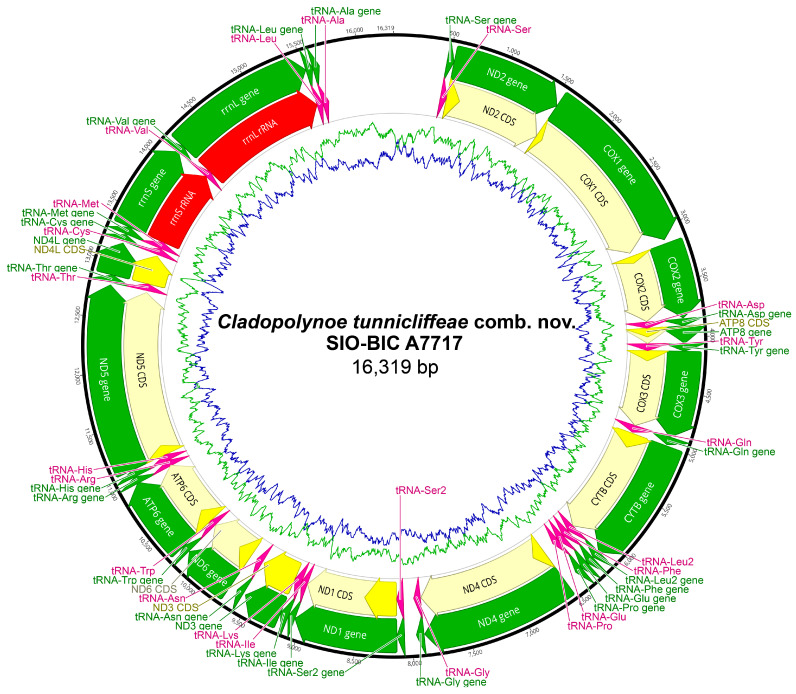
*Cladopolynoe tunnicliffeae* comb. nov. (SIO-BIC A7717) complete mitogenome. The thick black circle represents the mitogenome sequence, with numbers indicating base pairs. Green arrows represent genes, yellow arrows represent protein CDS, red arrows represent rRNAs, and pink arrows represent tRNAs. The direction of the arrow represents the direction of the gene. If genes are long enough, their names and descriptions (gene, CDS, rRNA, or tRNA) are written on the corresponding arrows; otherwise, this information is written next to the gene and connected to it via green (gene), yellow (CDS), red (rRNA), or pink (tRNA) lines. The blue line graph plots the GC content and the green line graph plots the AT content of the mitogenome within a sliding window of 45 nt bp at each individual nt bp position. Geneious Prime v. 2022.1.1 created by Biomatters was used to generate this figure. Available from https://www.geneious.com (accessed on 8 July 2024).

**Figure A15:**
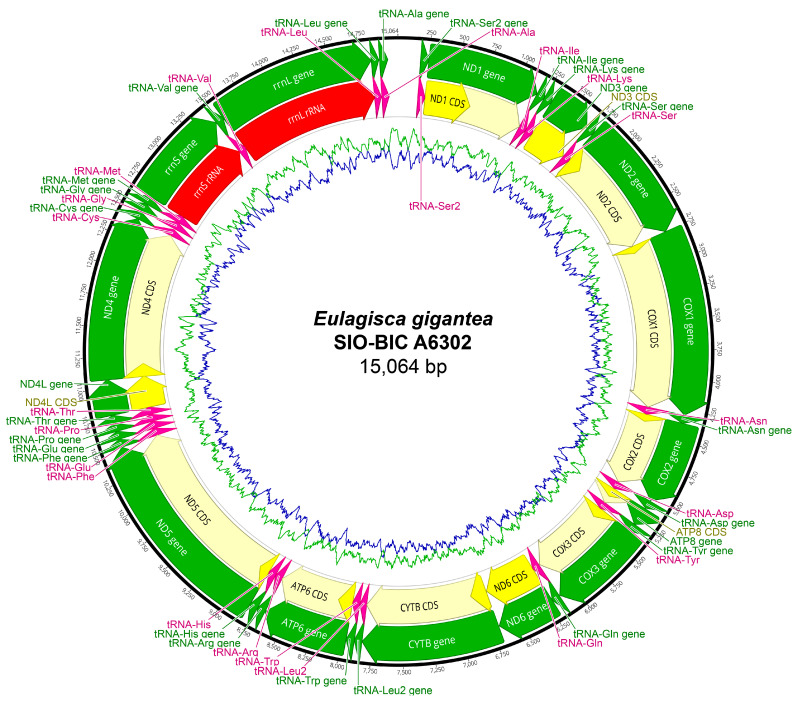
*Eulagisca gigantea* (SIO-BIC A6302) complete mitogenome. The thick black circle represents the mitogenome sequence, with numbers indicating base pairs. Green arrows represent genes, yellow arrows represent protein CDS, red arrows represent rRNAs, and pink arrows represent tRNAs. The direction of the arrow represents the direction of the gene. If genes are long enough, their names and descriptions (gene, CDS, rRNA, or tRNA) are written on the corresponding arrows; otherwise, this information is written next to the gene and connected to it via green (gene), yellow (CDS), red (rRNA), or pink (tRNA) lines. The blue line graph plots the GC content and the green line graph plots the AT content of the mitogenome within a sliding window of 45 nt bp at each individual nt bp position. Geneious Prime v. 2022.1.1 created by Biomatters was used to generate this figure. Available from https://www.geneious.com (accessed on 8 July 2024).

**Figure A16:**
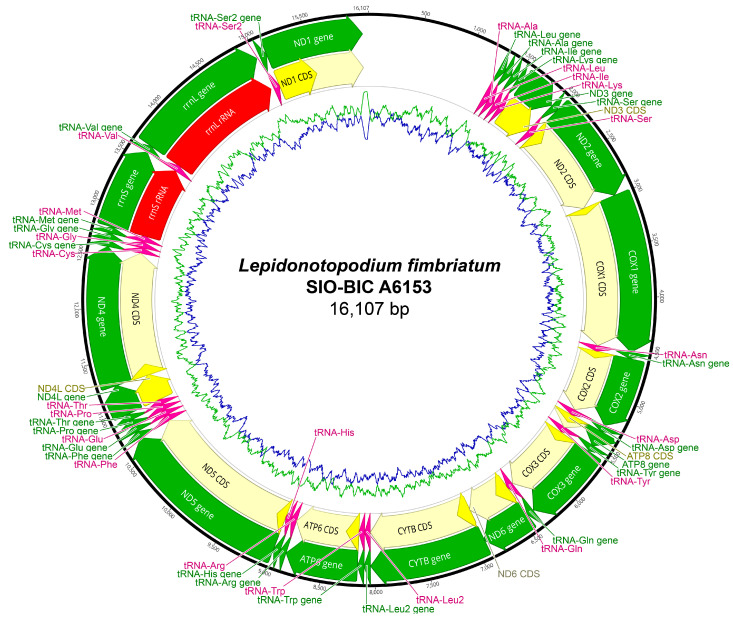
*Lepidonotopodium fimbriatum* (SIO-BIC A6153) complete mitogenome. The thick black circle represents the mitogenome sequence, with numbers indicating base pairs. Green arrows represent genes, yellow arrows represent protein CDS, red arrows represent rRNAs, and pink arrows represent tRNAs. The direction of the arrow represents the direction of the gene. If genes are long enough, their names and descriptions (gene, CDS, rRNA, or tRNA) are written on the corresponding arrows; otherwise, this information is written next to the gene and connected to it via green (gene), yellow (CDS), red (rRNA), or pink (tRNA) lines. The blue line graph plots the GC content and the green line graph plots the AT content of the mitogenome within a sliding window of 45 nt bp at each individual nt bp position. Geneious Prime v. 2022.1.1 created by Biomatters was used to generate this figure. Available from https://www.geneious.com (accessed on 8 July 2024).

**Figure A17:**
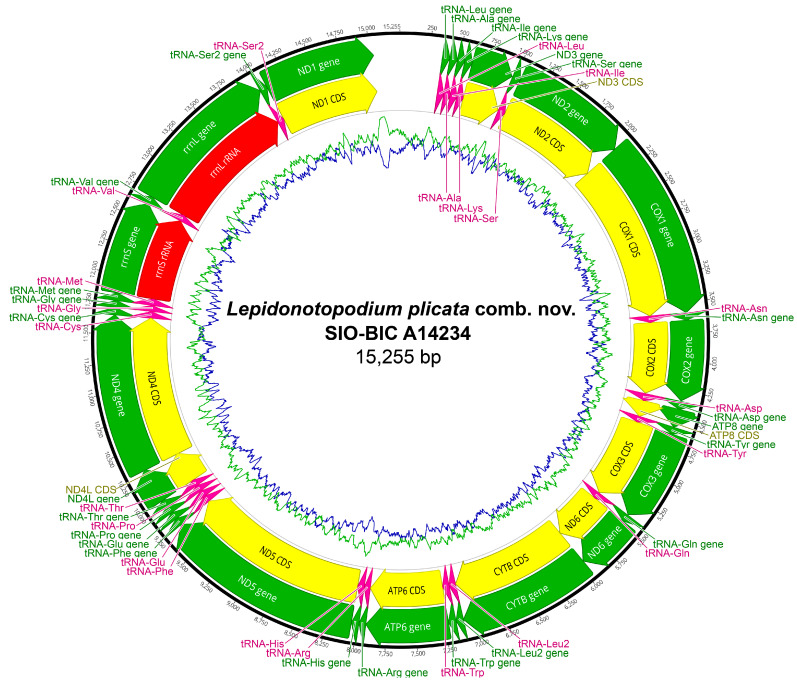
*Lepidonotopodium plicata* comb. nov. (SIO-BIC A14234) complete mitogenome. The thick black circle represents the mitogenome sequence, with numbers indicating base pairs. Green arrows represent genes, yellow arrows represent protein CDS, red arrows represent rRNAs, and pink arrows represent tRNAs. The direction of the arrow represents the direction of the gene. If genes are long enough, their names and descriptions (gene, CDS, rRNA, or tRNA) are written on the corresponding arrows; otherwise, this information is written next to the gene and connected to it via green (gene), yellow (CDS), red (rRNA), or pink (tRNA) lines. The blue line graph plots the GC content and the green line graph plots the AT content of the mitogenome within a sliding window of 45 nt bp at each individual nt bp position. Geneious Prime v. 2022.1.1 created by Biomatters was used to generate this figure. Available from https://www.geneious.com (accessed on 8 July 2024).

**Figure A18:**
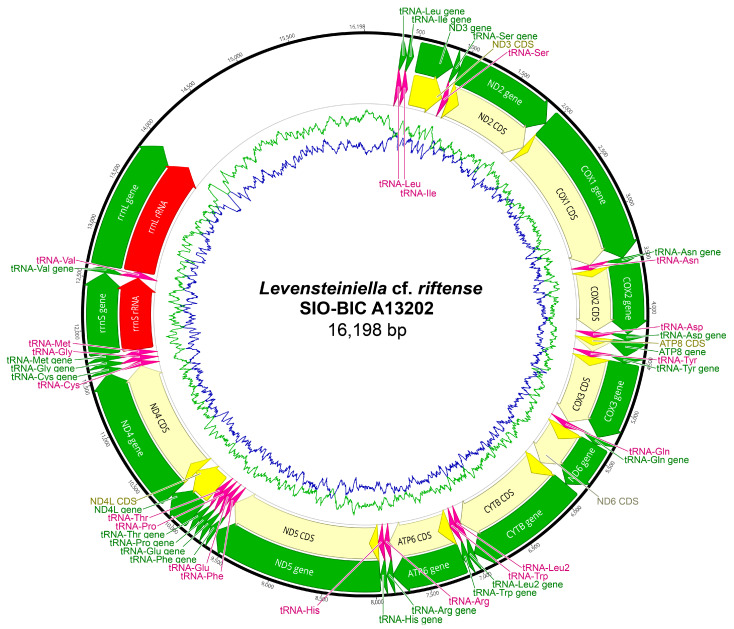
*Levensteiniella* cf. *riftense* (SIO-BIC A13202) mitogenome; missing genes include *NADH dehydrogenase subunit 1* (*ND1*), *tRNA-Ala*, *tRNA-Lys*, and *tRNA-Ser2*. The thick black circle represents the mitogenome sequence, with numbers indicating base pairs. Green arrows represent genes, yellow arrows represent protein CDS, red arrows represent rRNAs, and pink arrows represent tRNAs. The direction of the arrow represents the direction of the gene. If genes are long enough, their names and descriptions (gene, CDS, rRNA, or tRNA) are written on the corresponding arrows; otherwise, this information is written next to the gene and connected to it via green (gene), yellow (CDS), red (rRNA), or pink (tRNA) lines. The blue line graph plots the GC content and the green line graph plots the AT content of the mitogenome within a sliding window of 45 nt bp at each individual nt bp position. Geneious Prime v. 2022.1.1 created by Biomatters was used to generate this figure. Available from https://www.geneious.com (accessed on 8 July 2024).

**Figure A19:**
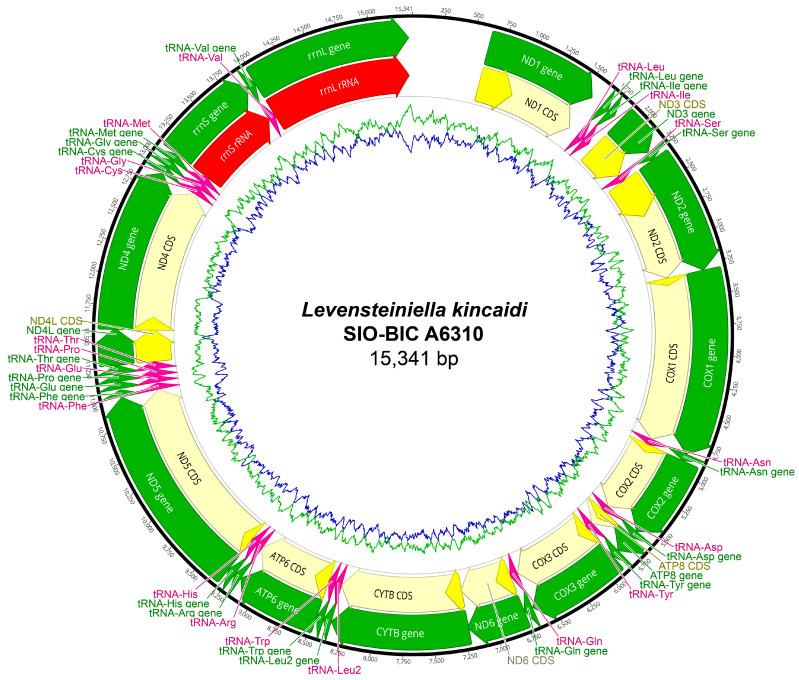
*Levensteiniella kincaidi* (SIO-BIC A6310) mitogenome; missing genes include *tRNA-Ala*, *tRNA-Lys*, and *tRNA-Ser2*. The thick black circle represents the mitogenome sequence, with numbers indicating base pairs. Green arrows represent genes, yellow arrows represent protein CDS, red arrows represent rRNAs, and pink arrows represent tRNAs. The direction of the arrow represents the direction of the gene. If genes are long enough, their names and descriptions (gene, CDS, rRNA, or tRNA) are written on the corresponding arrows; otherwise, this information is written next to the gene and connected to it via green (gene), yellow (CDS), red (rRNA), or pink (tRNA) lines. The blue line graph plots the GC content and the green line graph plots the AT content of the mitogenome within a sliding window of 45 nt bp at each individual nt bp position. Geneious Prime v. 2022.1.1 created by Biomatters was used to generate this figure. Available from https://www.geneious.com (accessed on 8 July 2024).

**Figure A20:**
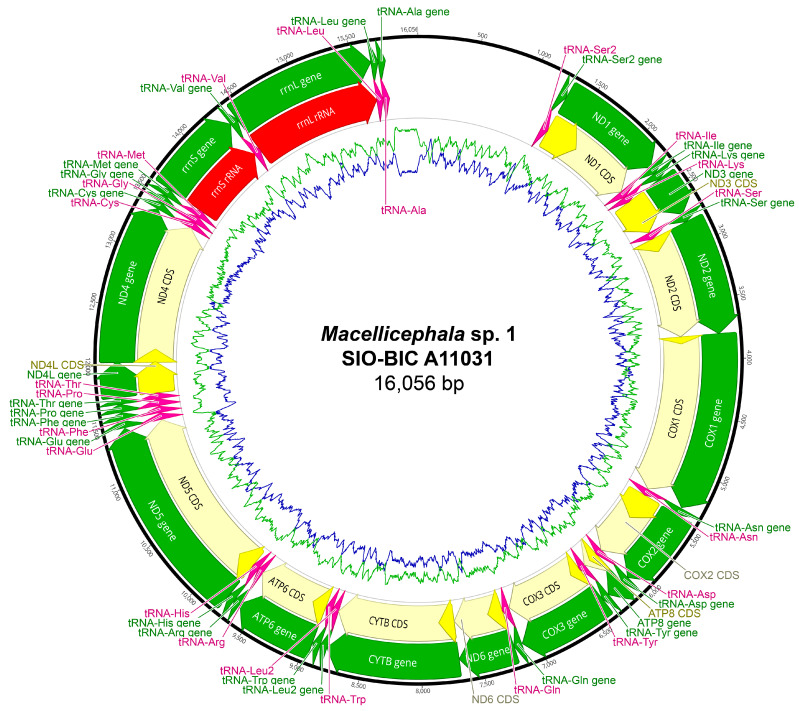
*Macellicephala* sp. 1 (SIO-BIC A11031) complete mitogenome. The thick black circle represents the mitogenome sequence, with numbers indicating base pairs. Green arrows represent genes, yellow arrows represent protein CDS, red arrows represent rRNAs, and pink arrows represent tRNAs. The direction of the arrow represents the direction of the gene. If genes are long enough, their names and descriptions (gene, CDS, rRNA, or tRNA) are written on the corresponding arrows; otherwise, this information is written next to the gene and connected to it via green (gene), yellow (CDS), red (rRNA), or pink (tRNA) lines. The blue line graph plots the GC content and the green line graph plots the AT content of the mitogenome within a sliding window of 45 nt bp at each individual nt bp position. Geneious Prime v. 2022.1.1 created by Biomatters was used to generate this figure. Available from https://www.geneious.com (accessed on 8 July 2024).

**Figure A21:**
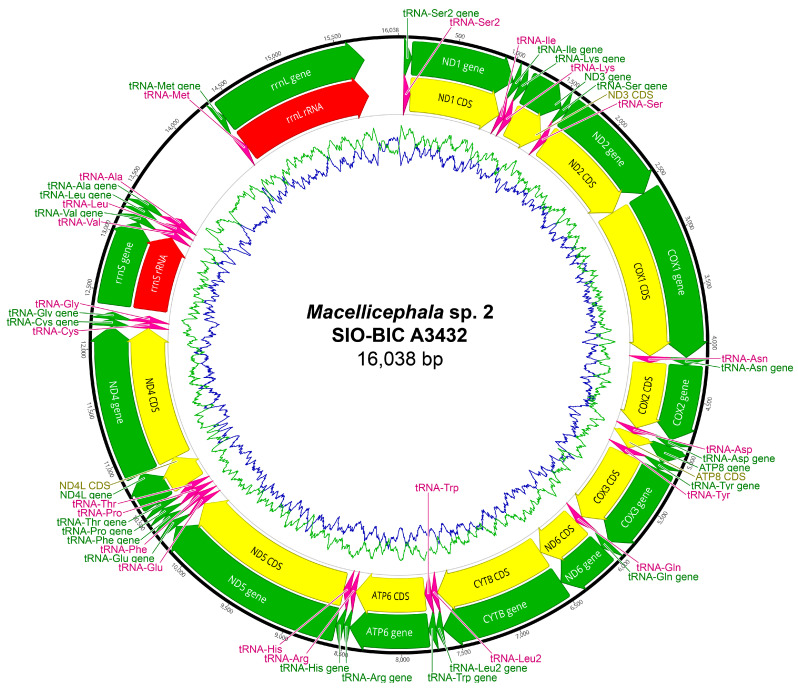
*Macellicephala* sp. 2 (SIO-BIC A3432) complete mitogenome. The thick black circle represents the mitogenome sequence, with numbers indicating base pairs. Green arrows represent genes, yellow arrows represent protein CDS, red arrows represent rRNAs, and pink arrows represent tRNAs. The direction of the arrow represents the direction of the gene. If genes are long enough, their names and descriptions (gene, CDS, rRNA, or tRNA) are written on the corresponding arrows; otherwise, this information is written next to the gene and connected to it via green (gene), yellow (CDS), red (rRNA), or pink (tRNA) lines. The blue line graph plots the GC content and the green line graph plots the AT content of the mitogenome within a sliding window of 45 nt bp at each individual nt bp position. Geneious Prime v. 2022.1.1 created by Biomatters was used to generate this figure. Available from https://www.geneious.com (accessed on 8 July 2024).

**Figure A22:**
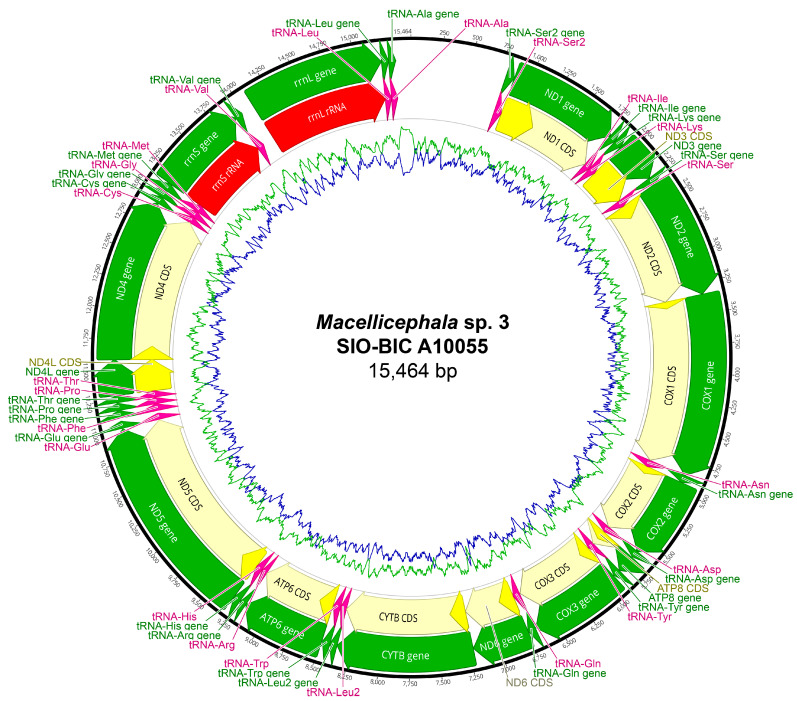
*Macellicephala* sp. 3 (SIO-BIC A10055) complete mitogenome. The thick black circle represents the mitogenome sequence, with numbers indicating base pairs. Green arrows represent genes, yellow arrows represent protein CDS, red arrows represent rRNAs, and pink arrows represent tRNAs. The direction of the arrow represents the direction of the gene. If genes are long enough, their names and descriptions (gene, CDS, rRNA, or tRNA) are written on the corresponding arrows; otherwise, this information is written next to the gene and connected to it via green (gene), yellow (CDS), red (rRNA), or pink (tRNA) lines. The blue line graph plots the GC content and the green line graph plots the AT content of the mitogenome within a sliding window of 45 nt bp at each individual nt bp position. Geneious Prime v. 2022.1.1 created by Biomatters was used to generate this figure. Available from https://www.geneious.com (accessed on 8 July 2024).

**Figure A23:**
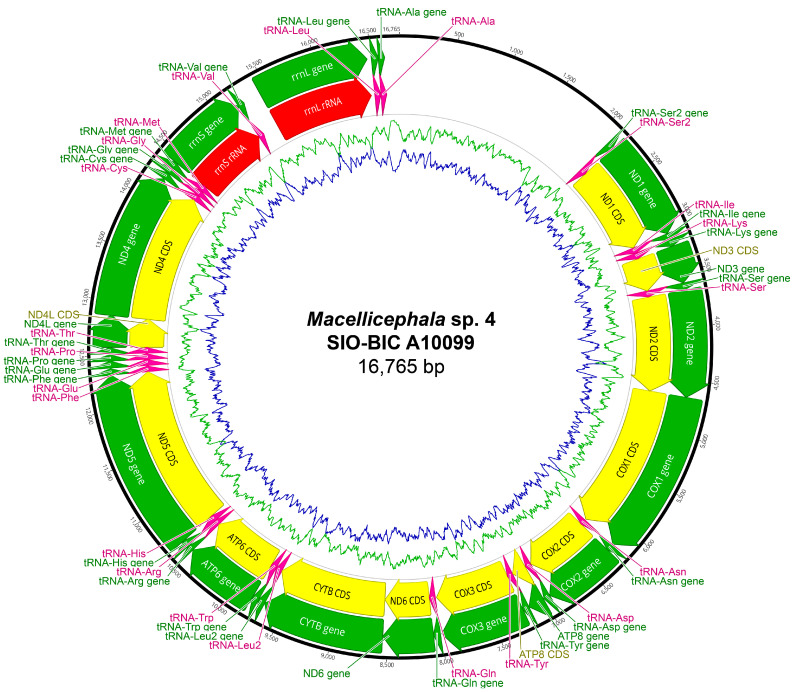
*Macellicephala* sp. 4 (SIO-BIC A10099) complete mitogenome. The thick black circle represents the mitogenome sequence, with numbers indicating base pairs. Green arrows represent genes, yellow arrows represent protein CDS, red arrows represent rRNAs, and pink arrows represent tRNAs. The direction of the arrow represents the direction of the gene. If genes are long enough, their names and descriptions (gene, CDS, rRNA, or tRNA) are written on the corresponding arrows; otherwise, this information is written next to the gene and connected to it via green (gene), yellow (CDS), red (rRNA), or pink (tRNA) lines. The blue line graph plots the GC content and the green line graph plots the AT content of the mitogenome within a sliding window of 45 nt bp at each individual nt bp position. Geneious Prime v. 2022.1.1 created by Biomatters was used to generate this figure. Available from https://www.geneious.com (accessed on 8 July 2024).

**Figure A24:**
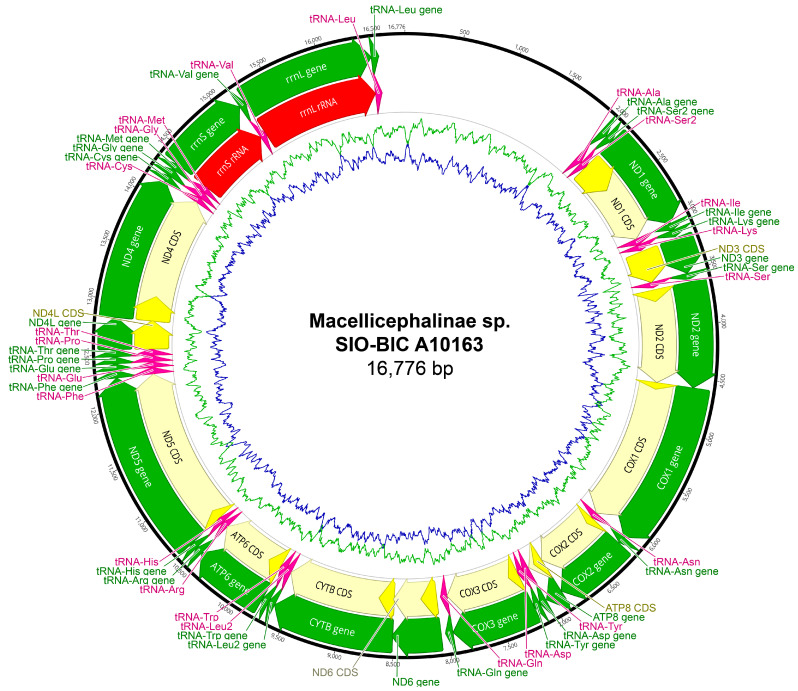
Macellicephalinae sp. (SIO-BIC A10163) complete mitogenome. The thick black circle represents the mitogenome sequence, with numbers indicating base pairs. Green arrows represent genes, yellow arrows represent protein CDS, red arrows represent rRNAs, and pink arrows represent tRNAs. The direction of the arrow represents the direction of the gene. If genes are long enough, their names and descriptions (gene, CDS, rRNA, or tRNA) are written on the corresponding arrows; otherwise, this information is written next to the gene and connected to it via green (gene), yellow (CDS), red (rRNA), or pink (tRNA) lines. The blue line graph plots the GC content and the green line graph plots the AT content of the mitogenome within a sliding window of 45 nt bp at each individual nt bp position. Geneious Prime v. 2022.1.1 created by Biomatters was used to generate this figure. Available from https://www.geneious.com (accessed on 8 July 2024).

**Figure A25:**
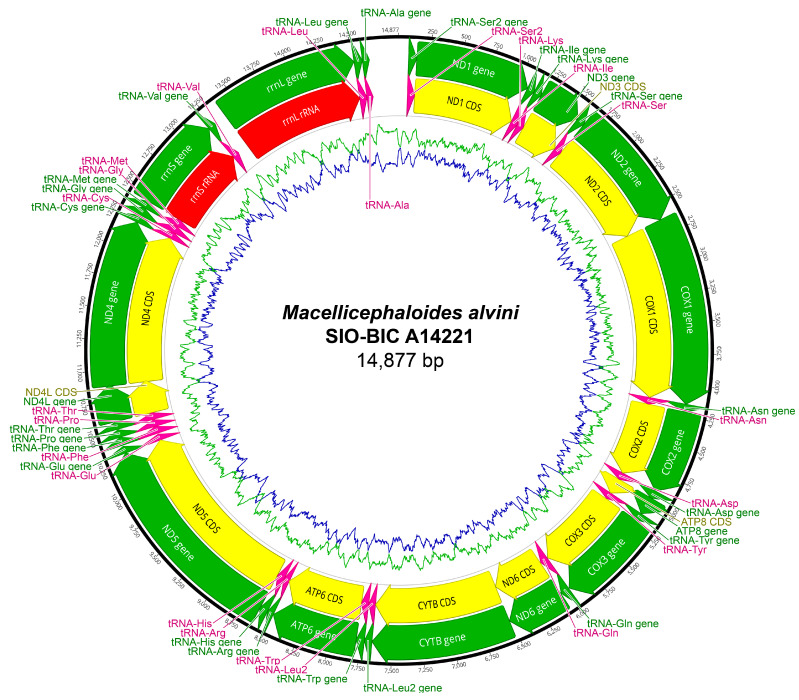
*Macellicephaloides alvini* (SIO-BIC A14221) complete mitogenome. The thick black circle represents the mitogenome sequence, with numbers indicating base pairs. Green arrows represent genes, yellow arrows represent protein CDS, red arrows represent rRNAs, and pink arrows represent tRNAs. The direction of the arrow represents the direction of the gene. If genes are long enough, their names and descriptions (gene, CDS, rRNA, or tRNA) are written on the corresponding arrows; otherwise, this information is written next to the gene and connected to it via green (gene), yellow (CDS), red (rRNA), or pink (tRNA) lines. The blue line graph plots the GC content and the green line graph plots the AT content of the mitogenome within a sliding window of 45 nt bp at each individual nt bp position. Geneious Prime v. 2022.1.1 created by Biomatters was used to generate this figure. Available from https://www.geneious.com (accessed on 8 July 2024).

**Figure A26:**
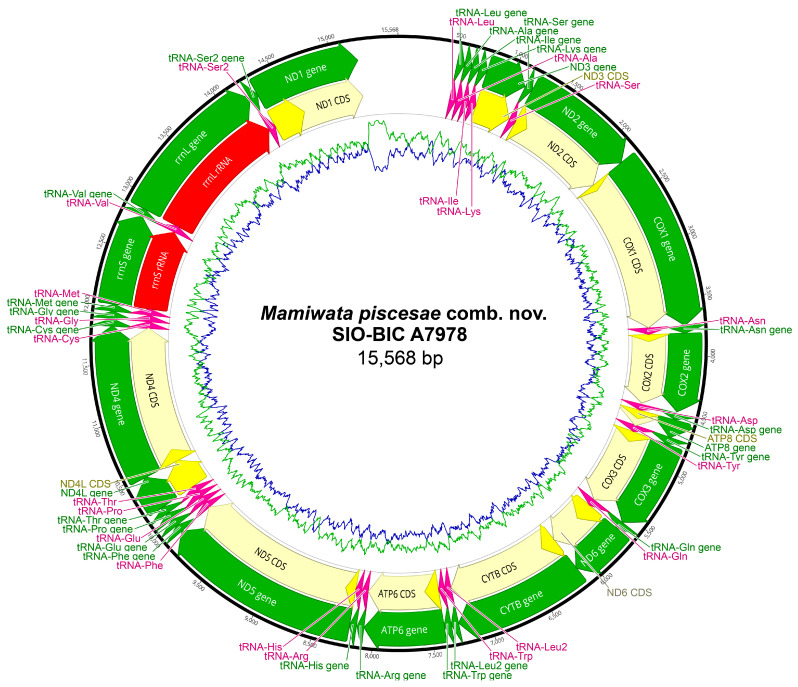
*Mamiwata piscesae* comb. nov. (SIO-BIC A7978) complete mitogenome. The thick black circle represents the mitogenome sequence, with numbers indicating base pairs. Green arrows represent genes, yellow arrows represent protein CDS, red arrows represent rRNAs, and pink arrows represent tRNAs. The direction of the arrow represents the direction of the gene. If genes are long enough, their names and descriptions (gene, CDS, rRNA, or tRNA) are written on the corresponding arrows; otherwise, this information is written next to the gene and connected to it via green (gene), yellow (CDS), red (rRNA), or pink (tRNA) lines. The blue line graph plots the GC content and the green line graph plots the AT content of the mitogenome within a sliding window of 45 nt bp at each individual nt bp position. Geneious Prime v. 2022.1.1 created by Biomatters was used to generate this figure. Available from https://www.geneious.com (accessed on 8 July 2024).

**Figure A27:**
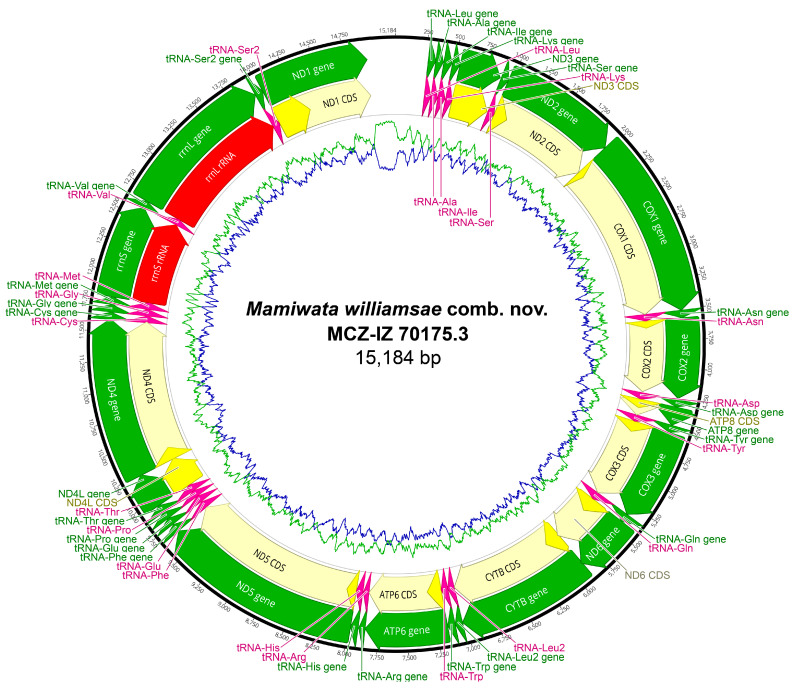
*Mamiwata williamsae* comb. nov. (MCZ-IZ 70175.3) complete mitogenome. The thick black circle represents the mitogenome sequence, with numbers indicating base pairs. Green arrows represent genes, yellow arrows represent protein CDS, red arrows represent rRNAs, and pink arrows represent tRNAs. The direction of the arrow represents the direction of the gene. If genes are long enough, their names and descriptions (gene, CDS, rRNA, or tRNA) are written on the corresponding arrows; otherwise, this information is written next to the gene and connected to it via green (gene), yellow (CDS), red (rRNA), or pink (tRNA) lines. The blue line graph plots the GC content and the green line graph plots the AT content of the mitogenome within a sliding window of 45 nt bp at each individual nt bp position. Geneious Prime v. 2022.1.1 created by Biomatters was used to generate this figure. Available from https://www.geneious.com (accessed on 8 July 2024).

**Figure A28:**
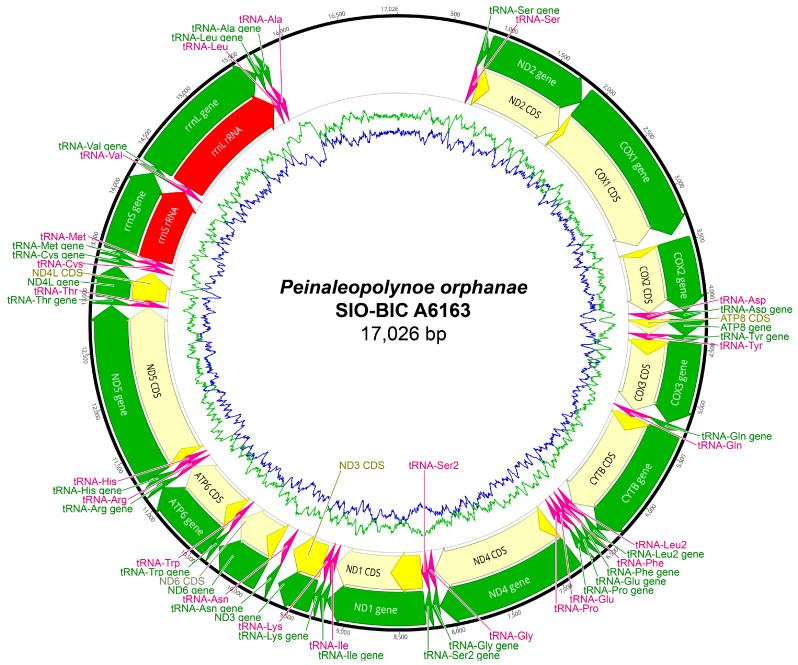
*Peinaleopolynoe orphanae* (SIO-BIC A6163) complete mitogenome. The thick black circle represents the mitogenome sequence, with numbers indicating base pairs. Green arrows represent genes, yellow arrows represent protein CDS, red arrows represent rRNAs, and pink arrows represent tRNAs. The direction of the arrow represents the direction of the gene. If genes are long enough, their names and descriptions (gene, CDS, rRNA, or tRNA) are written on the corresponding arrows; otherwise, this information is written next to the gene and connected to it via green (gene), yellow (CDS), red (rRNA), or pink (tRNA) lines. The blue line graph plots the GC content and the green line graph plots the AT content of the mitogenome within a sliding window of 45 nt bp at each individual nt bp position. Geneious Prime v. 2022.1.1 created by Biomatters was used to generate this figure. Available from https://www.geneious.com (accessed on 8 July 2024).

**Figure A29:**
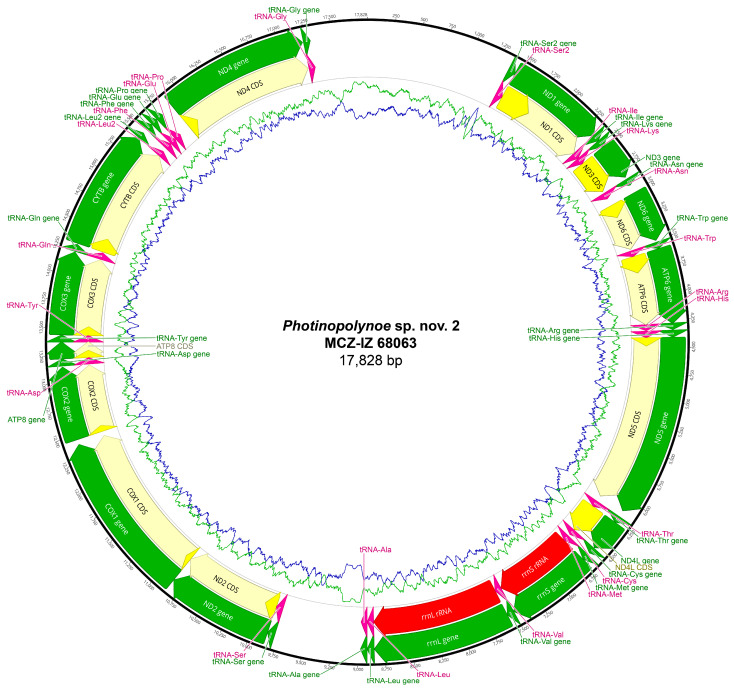
*Photinopolynoe* sp. nov. 2 (MCZ-IZ 68063) complete mitogenome. The thick black circle represents the mitogenome sequence, with numbers indicating base pairs. Green arrows represent genes, yellow arrows represent protein CDS, red arrows represent rRNAs, and pink arrows represent tRNAs. The direction of the arrow represents the direction of the gene. If genes are long enough, their names and descriptions (gene, CDS, rRNA, or tRNA) are written on the corresponding arrows; otherwise, this information is written next to the gene and connected to it via green (gene), yellow (CDS), red (rRNA), or pink (tRNA) lines. The blue line graph plots the GC content and the green line graph plots the AT content of the mitogenome within a sliding window of 45 nt bp at each individual nt bp position. Geneious Prime v. 2022.1.1 created by Biomatters was used to generate this figure. Available from https://www.geneious.com (accessed on 8 July 2024).

**Figure A30:**
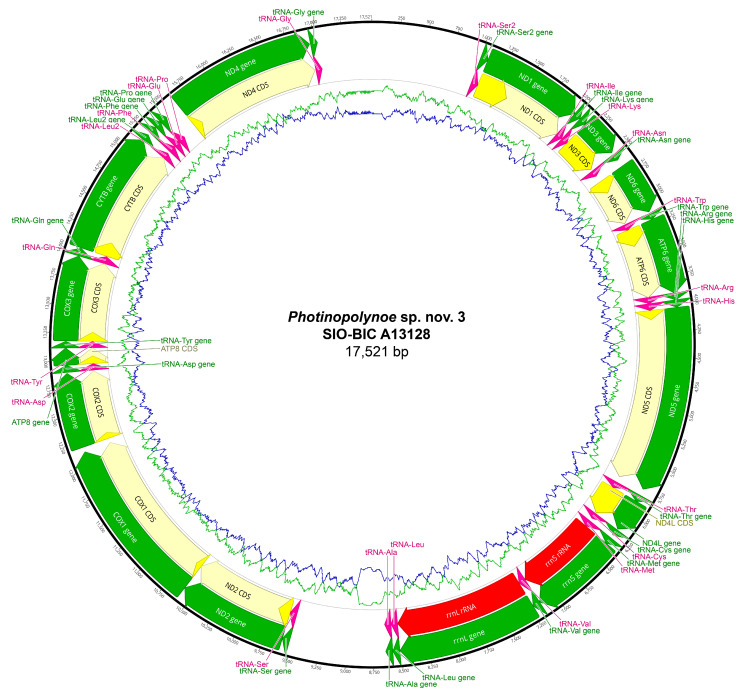
*Photinopolynoe* sp. nov. 3 (SIO-BIC A13128) complete mitogenome. The thick black circle represents the mitogenome sequence, with numbers indicating base pairs. Green arrows represent genes, yellow arrows represent protein CDS, red arrows represent rRNAs, and pink arrows represent tRNAs. The direction of the arrow represents the direction of the gene. If genes are long enough, their names and descriptions (gene, CDS, rRNA, or tRNA) are written on the corresponding arrows; otherwise, this information is written next to the gene and connected to it via green (gene), yellow (CDS), red (rRNA), or pink (tRNA) lines. The blue line graph plots the GC content and the green line graph plots the AT content of the mitogenome within a sliding window of 45 nt bp at each individual nt bp position. Geneious Prime v. 2022.1.1 created by Biomatters was used to generate this figure. Available from https://www.geneious.com (accessed on 8 July 2024).

**Figure A31:**
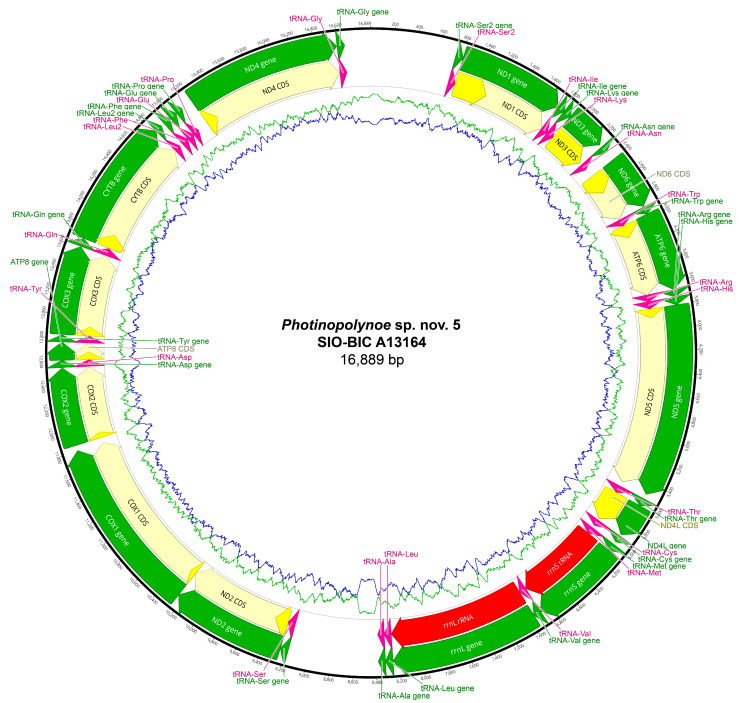
*Photinopolynoe* sp. nov. 5 (SIO-BIC A13164) complete mitogenome. The thick black circle represents the mitogenome sequence, with numbers indicating base pairs. Green arrows represent genes, yellow arrows represent protein CDS, red arrows represent rRNAs, and pink arrows represent tRNAs. The direction of the arrow represents the direction of the gene. If genes are long enough, their names and descriptions (gene, CDS, rRNA, or tRNA) are written on the corresponding arrows; otherwise, this information is written next to the gene and connected to it via green (gene), yellow (CDS), red (rRNA), or pink (tRNA) lines. The blue line graph plots the GC content and the green line graph plots the AT content of the mitogenome within a sliding window of 45 nt bp at each individual nt bp position. Geneious Prime v. 2022.1.1 created by Biomatters was used to generate this figure. Available from https://www.geneious.com (accessed on 8 July 2024).

**Figure A32:**
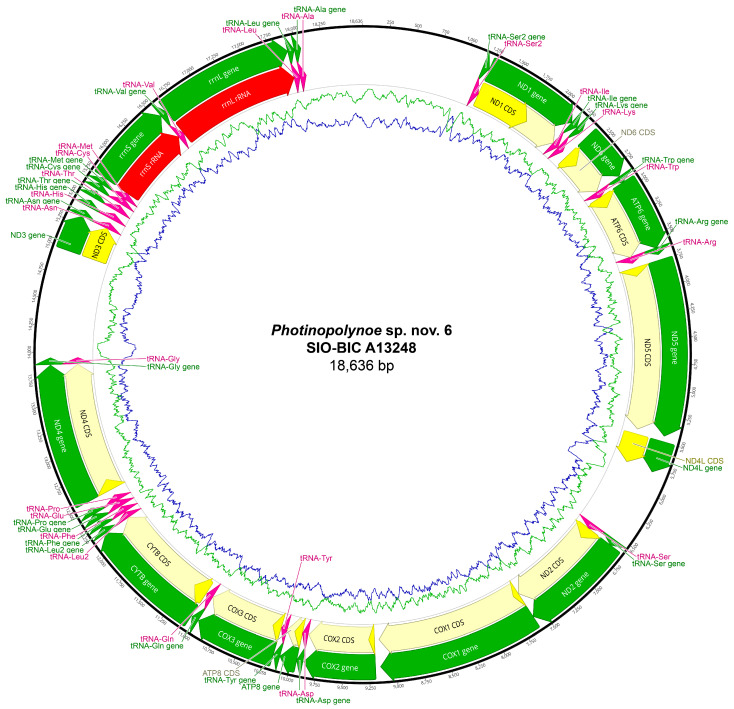
*Photinopolynoe* sp. nov. 6 (SIO-BIC A13248) complete mitogenome. The thick black circle represents the mitogenome sequence, with numbers indicating base pairs. Green arrows represent genes, yellow arrows represent protein CDS, red arrows represent rRNAs, and pink arrows represent tRNAs. The direction of the arrow represents the direction of the gene. If genes are long enough, their names and descriptions (gene, CDS, rRNA, or tRNA) are written on the corresponding arrows; otherwise, this information is written next to the gene and connected to it via green (gene), yellow (CDS), red (rRNA), or pink (tRNA) lines. The blue line graph plots the GC content and the green line graph plots the AT content of the mitogenome within a sliding window of 45 nt bp at each individual nt bp position. Geneious Prime v. 2022.1.1 created by Biomatters was used to generate this figure. Available from https://www.geneious.com (accessed on 8 July 2024).

**Figure A33:**
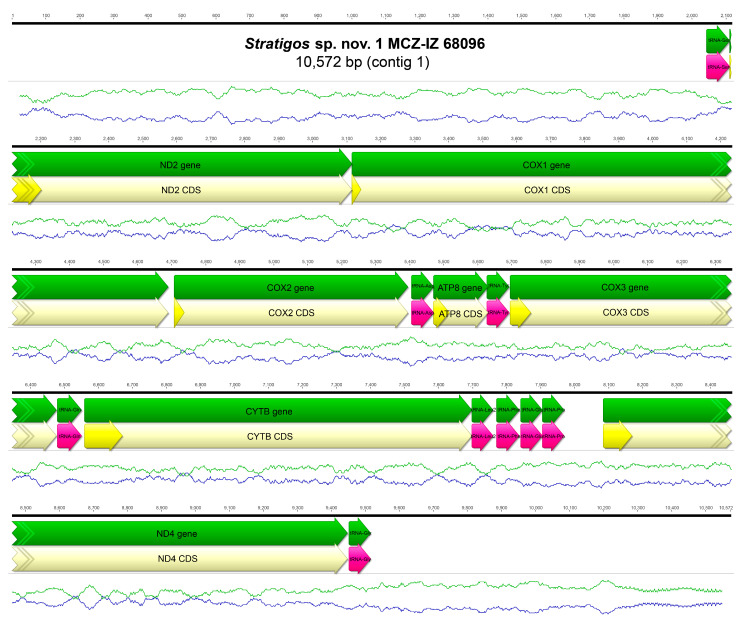
*Stratigos* sp. nov. 1 (MCZ-IZ 68096) incomplete mitogenome contig 1. The thick black line represents the partial mitogenome sequence, with numbers indicating base pairs. Green arrows represent genes, yellow arrows represent protein CDS, red arrows represent rRNAs, and pink arrows represent tRNAs. The direction of the arrow represents the direction of the gene. Gene names and descriptions (gene, CDS, rRNA, or tRNA) are written on the corresponding arrows. The blue line graph plots the GC content and the green line graph plots the AT content of the mitogenome within a sliding window of 45 nt bp at each individual nt bp position. Geneious Prime v. 2022.1.1 created by Biomatters was used to generate this figure. Available from https://www.geneious.com (accessed on 8 July 2024).

**Figure A34:**
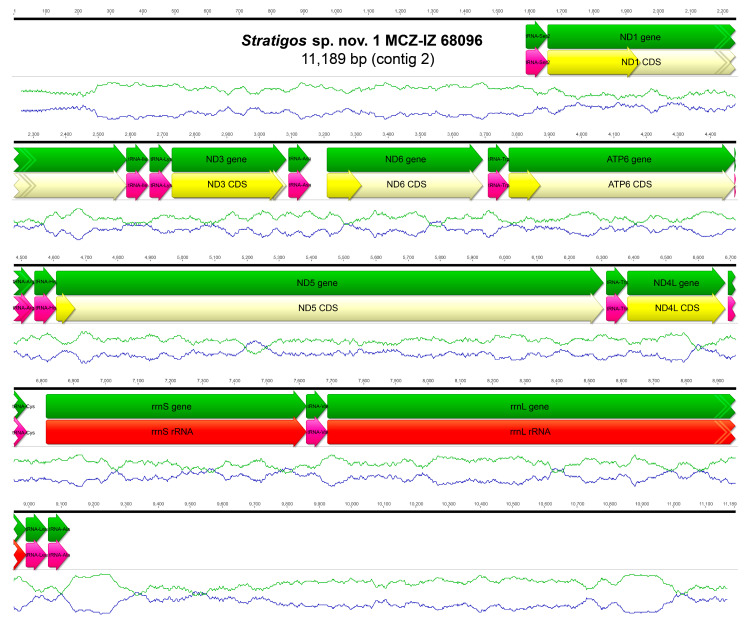
*Stratigos* sp. nov. 1 (MCZ-IZ 68096) incomplete mitogenome contig 2. The thick black line represents the partial mitogenome sequence, with numbers indicating base pairs. Green arrows represent genes, yellow arrows represent protein CDS, red arrows represent rRNAs, and pink arrows represent tRNAs. The direction of the arrow represents the direction of the gene. Gene names and descriptions (gene, CDS, rRNA, or tRNA) are written on the corresponding arrows. The blue line graph plots the GC content and the green line graph plots the AT content of the mitogenome within a sliding window of 45 nt bp at each individual nt bp position. Geneious Prime v. 2022.1.1 created by Biomatters was used to generate this figure. Available from https://www.geneious.com (accessed on 8 July 2024).

**Figure A35:**
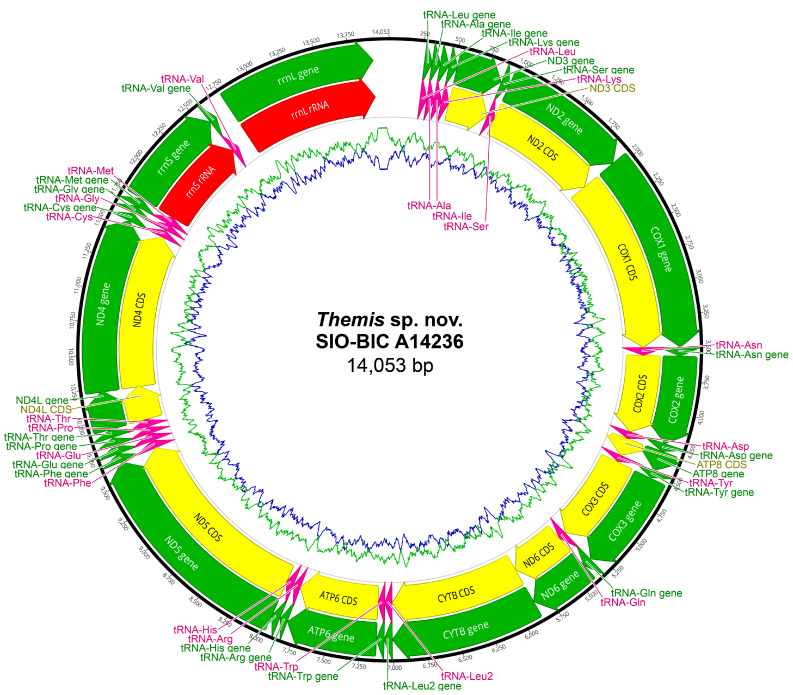
*Themis* sp. nov. (SIO-BIC A14236) mitogenome; missing genes include *ND1* and *tRNA-Ser2*. The thick black circle represents the mitogenome sequence, with numbers indicating base pairs. Green arrows represent genes, yellow arrows represent protein CDS, red arrows represent rRNAs, and pink arrows represent tRNAs. The direction of the arrow represents the direction of the gene. If genes are long enough, their names and descriptions (gene, CDS, rRNA, or tRNA) are written on the corresponding arrows; otherwise, this information is written next to the gene and connected to it via green (gene), yellow (CDS), red (rRNA), or pink (tRNA) lines. The blue line graph plots the GC content and the green line graph plots the AT content of the mitogenome within a sliding window of 45 nt bp at each individual nt bp position. Geneious Prime v. 2022.1.1 created by Biomatters was used to generate this figure. Available from https://www.geneious.com (accessed on 8 July 2024).

**Figure A36:**
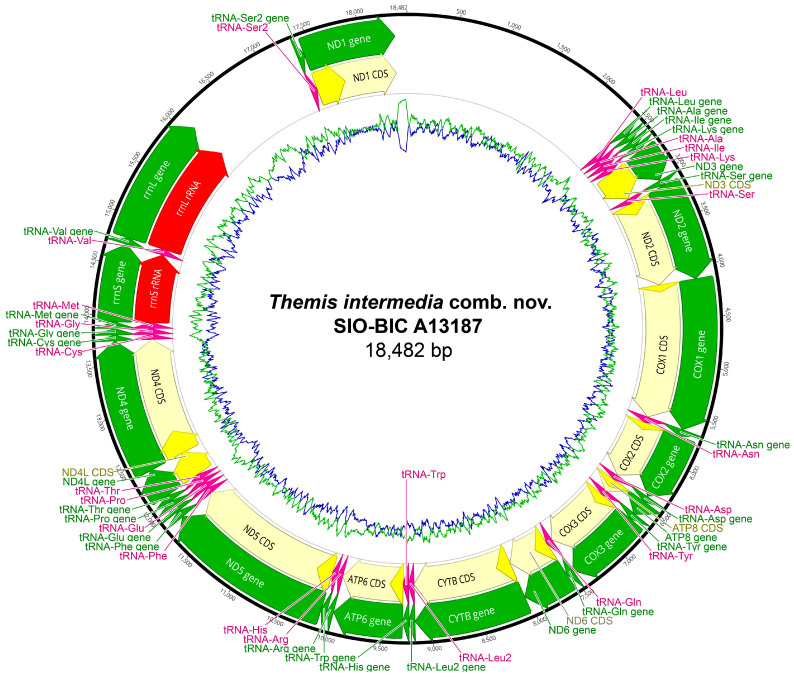
*Themis intermedia* comb. nov. (SIO-BIC A13187) complete mitogenome. The thick black circle represents the mitogenome sequence, with numbers indicating base pairs. Green arrows represent genes, yellow arrows represent protein CDS, red arrows represent rRNAs, and pink arrows represent tRNAs. The direction of the arrow represents the direction of the gene. If genes are long enough, their names and descriptions (gene, CDS, rRNA, or tRNA) are written on the corresponding arrows; otherwise, this information is written next to the gene and connected to it via green (gene), yellow (CDS), red (rRNA), or pink (tRNA) lines. The blue line graph plots the GC content and the green line graph plots the AT content of the mitogenome within a sliding window of 45 nt bp at each individual nt bp position. Geneious Prime v. 2022.1.1 created by Biomatters was used to generate this figure. Available from https://www.geneious.com (accessed on 8 July 2024).

**Figure A37:**
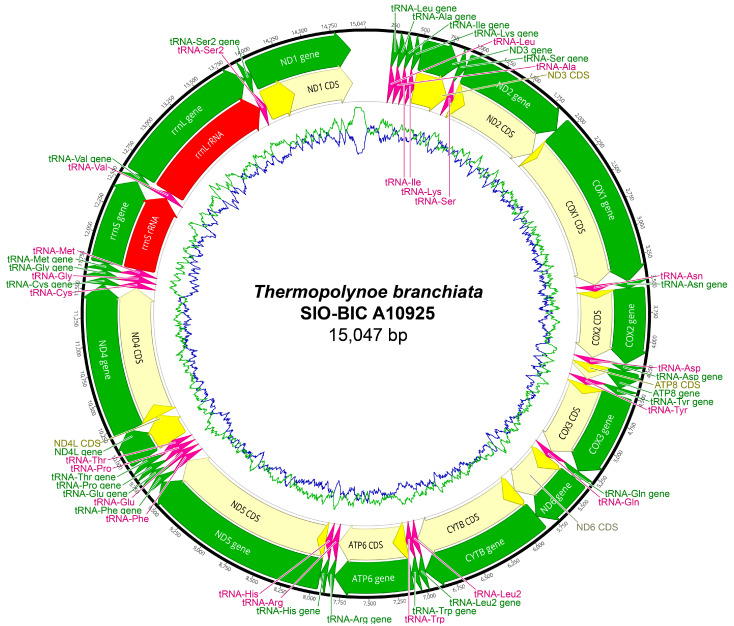
*Thermopolynoe branchiata* (SIO-BIC A10925) complete mitogenome. The thick black circle represents the mitogenome sequence, with numbers indicating base pairs. Green arrows represent genes, yellow arrows represent protein CDS, red arrows represent rRNAs, and pink arrows represent tRNAs. The direction of the arrow represents the direction of the gene. If genes are long enough, their names and descriptions (gene, CDS, rRNA, or tRNA) are written on the corresponding arrows; otherwise, this information is written next to the gene and connected to it via green (gene), yellow (CDS), red (rRNA), or pink (tRNA) lines. The blue line graph plots the GC content and the green line graph plots the AT content of the mitogenome within a sliding window of 45 nt bp at each individual nt bp position. Geneious Prime v. 2022.1.1 created by Biomatters was used to generate this figure. Available from https://www.geneious.com (accessed on 8 July 2024).

Mitogenome lengths (14,053–18,636 bp), nucleotide content, skews, and codon representation are summarized in Tables S3–S5. General mitogenome organization is depicted in Figure A5, Figure A6, Figure A7, Figure A8, Figure A9, Figure A10, Figure A11, Figure A12, Figure A13, Figure A14, Figure A15, Figure A16, Figure A17, Figure A18, Figure A19, Figure A20, Figure A21, Figure A22, Figure A23, Figure A24, Figure A25, Figure A26, Figure A27, Figure A28, Figure A29, Figure A30, Figure A31, Figure A32, Figure A33, Figure A34, Figure A35, Figure A36 and Figure A37. From the 31 novel mitogenomes sequenced and assembled here, that of *Stratigos* sp. nov. 1 (Figure A33 and Figure A34) and *Branchipolynoe trifurcus* comb. nov. (Figure A10 and Figure A11) produced two incomplete mitochondrial contigs (Tables S1 and S3). For the first of these species, all 37 mitochondrial genes except *tRNA-Met* were present in both contigs; for the second, all genes except *tRNA-Gly* were present in both contigs, although *ND4* was also incomplete in contig 1. Assembly into a single contig was successful for all other species, although the following loci were missing (Table S3): *ND1*, *tRNA-Ala*, *tRNA-Lys*, and *tRNA-Ser2* for *Levensteiniella* cf. *riftense* (Figure A18); *tRNA-Ala*, *tRNA-Lys*, and *tRNA-Ser2* for *Levensteiniella kincaidi* (Figure A19); and *ND1* and *tRNA-Ser2* for *Themis* sp. nov. (Figure A35).

For all 31 polynoids, the mitogenome GC content was less than the AT content; GC content ranged from 27.61 to 42.11%, with *Macellicephala* sp. 4 having the lowest and *Themis intermedia* comb. nov. having the highest GC content (Table S3). Overall mitogenome GC skew ranged from −0.4195 (*Thermopolynoe branchiata*) to −0.1593 (Macellicephalinae sp.); AT skew ranged from −0.1301 (*Macellicephala* sp. 3) to −0.0011 (*Stratigos* sp. nov. 1, contig 2) (Table S3). The GC/AT contents and skews of each of the 13 PCGs and 2 rRNAs per taxon can be found in Table S4.

Over- and under-represented codons for each species (according to RSCU analysis of mitochondrial PCGs) are detailed in Table S5. The following 13 codons were under-represented in all 31 polynoids: GCG (alanine), AAG (lysine), CTG and TTG (leucine), start codon ATG (methionine), CCG (proline), CAG (glutamine), ACG (threonine), TGG (tryptophan), and four of the eight serine-coding codons (AGC, AGG, AGT, and TCG). Other commonly under-represented codons included: GAG (glutamic acid), under-represented in all polynoids except Macellicephalinae sp.; CGG (arginine), under-represented in all polynoids except *Themis* sp. nov.; GTG and GTC (valine), under-represented in 30 and 18 species, respectively; and termination codon TAG, under-represented in 27 taxa. Preferred codons included: AAA (lysine) and CAA (glutamic acid), which were over-represented in all 31 polynoids; TCT (serine), across all except *Themis intermedia* comb. nov.; GAA (glutamine) for all except *Macellicephala* sp. 3 and Macellicephalinae sp.; as well as start codon ATA (methionine), TGA (tryptophan), CGA (arginine), TCC (serine), GTA (valine), GGA (glycine), CTT (leucine), CCT (proline), and termination codon TAA, each of which was found to be over-represented in at least 70% of polynoids.

As expected, the predominant start codon for all PCGs was ATG, yet alternative start codons were occasionally used:

- ATA: *Branchiplicatus cupreus* (*ND4*); *Branchipolynoe* cf. *marianus* (*ND6*); Macellicephalinae sp. (*ND2*, *ND3*, and *ND6*);
- ATC: *Macellicephala* sp. 2 (*ND5*);
- ATT: *Macellicephala* sp. 2 (*ATP8*);
- CTG: *Macellicephala* sp. 3 and *Macellicephaloides alvini* (*ND3*);
- GTG: *Macellicephala* sp. 4 (*ND3*); *Thermopolynoe branchiata* (*ATP8*);
- TTG: Macellicephalinae sp. (*ND1* and *ND4L*).

*CYTB* and *ND2* were exclusively terminated using TAA, whereas a combination of TAA and TAG were used for all other loci. The first of these was the most common for all loci except *ATP6*, the only locus for which TAG was predominant. In many cases across the 13 PCGs, TAA stop codons were completed by the addition of 3′ A residues to the messenger RNA (mRNA) [110].

### 3.4. Aphroditiformia Mitochondrial Gene Order Rearrangement

**Figure 3:**
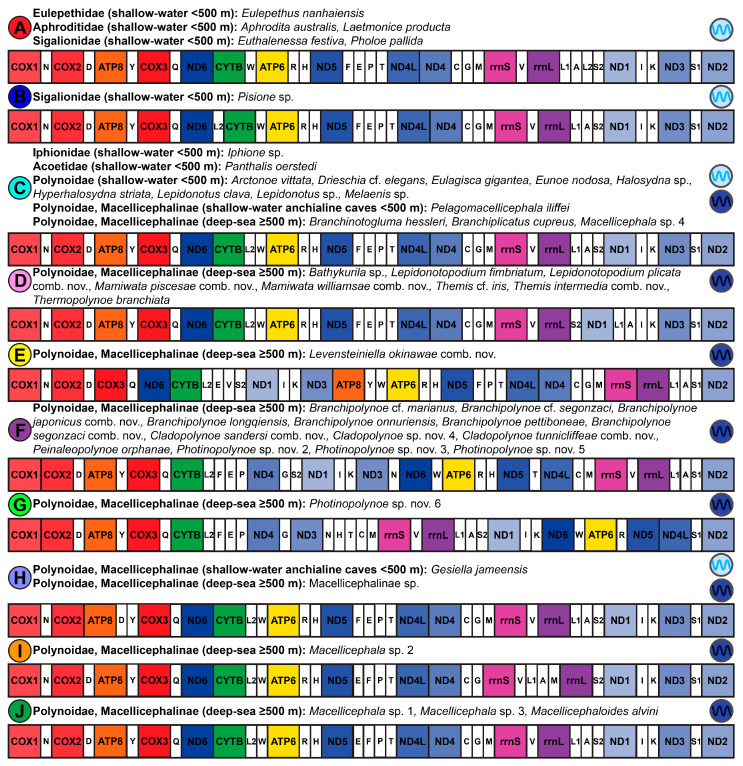
Mitochondrial gene orders, including the 13 protein-coding genes (PCGs), two ribosomal RNA genes (rRNAs), and 22 transfer RNA genes (tRNAs), present in Aphroditiformia. The light blue wave symbol indicates that the gene order is present in shallow-water scaleworms, and the dark blue wave symbol indicates that the gene order is present in deep-sea scaleworms.

Ten mitochondrial gene orders were present within Aphroditiformia when considering the 13 PCGs, two rRNAs, and 22 tRNAs (Figure 3). Two of these (gene orders A and B) are exclusive to shallow-water aphroditiforms, and two others (gene orders C and H) are shared by taxa spanning different depths, while the remaining six gene orders are exclusive to deep-sea lineages. Many gene orders are characteristic of individual terminals, including gene orders B (*Pisione* sp.), E (*Levensteiniella okinawae* comb. nov.), G (*Photinopolynoe* sp. nov. 6), and I (*Macellicephala* sp. 2), or present in relatively small clades (gene orders H and J, present in 2 and 3 terminals, respectively).

**Figure 4:**
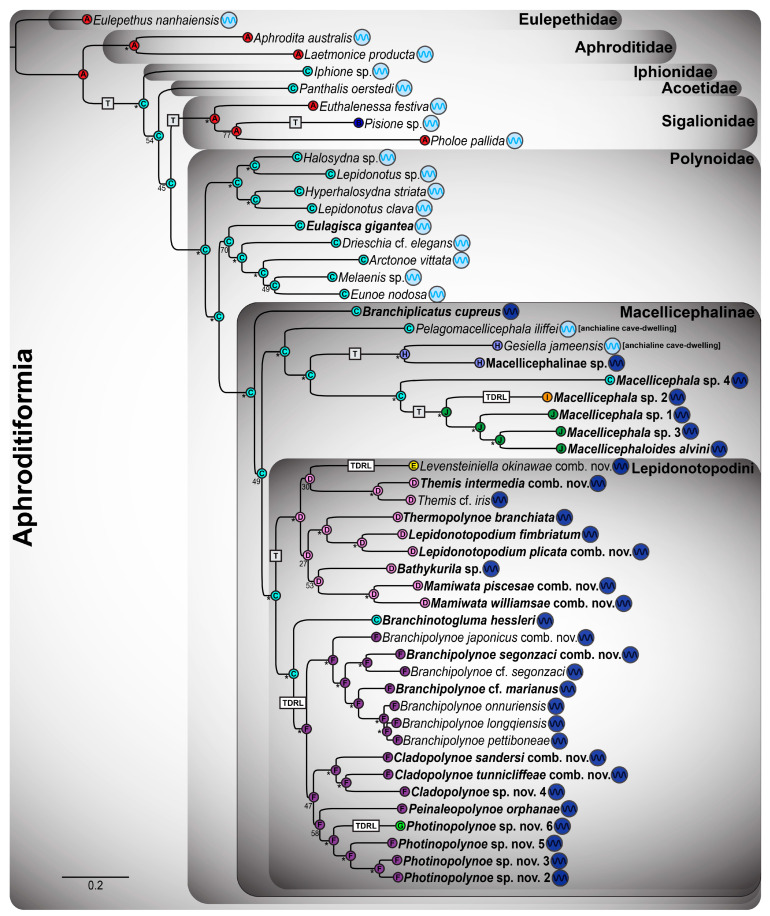
TreeREx ancestral state reconstructions of mitochondrial gene order and most parsimonious gene order rearrangement scenarios, based on the 13 PCGs, two rRNAs, and 22 tRNAs. Mitochondrial gene orders are indicated at both ancestral nodes and tips. Colors and letters inside the circles correspond to the gene orders identified in Figure 3. Gene order rearrangement scenarios are listed on corresponding branches. Numbers next to nodes are ML bootstrap percentages. Key: * indicates support values ≥ 95%, T = transposition, TDRL = tandem duplication-random loss. Light blue wave symbols at the tips of taxa identify shallow-water scaleworms, and dark blue wave symbols identify deep-sea scaleworms. Taxon names set in bold denote the scaleworm species for which mitogenomes were assembled in this study. Taxa that had missing or incomplete mitochondrial genes were excluded.

**Figure 5:**
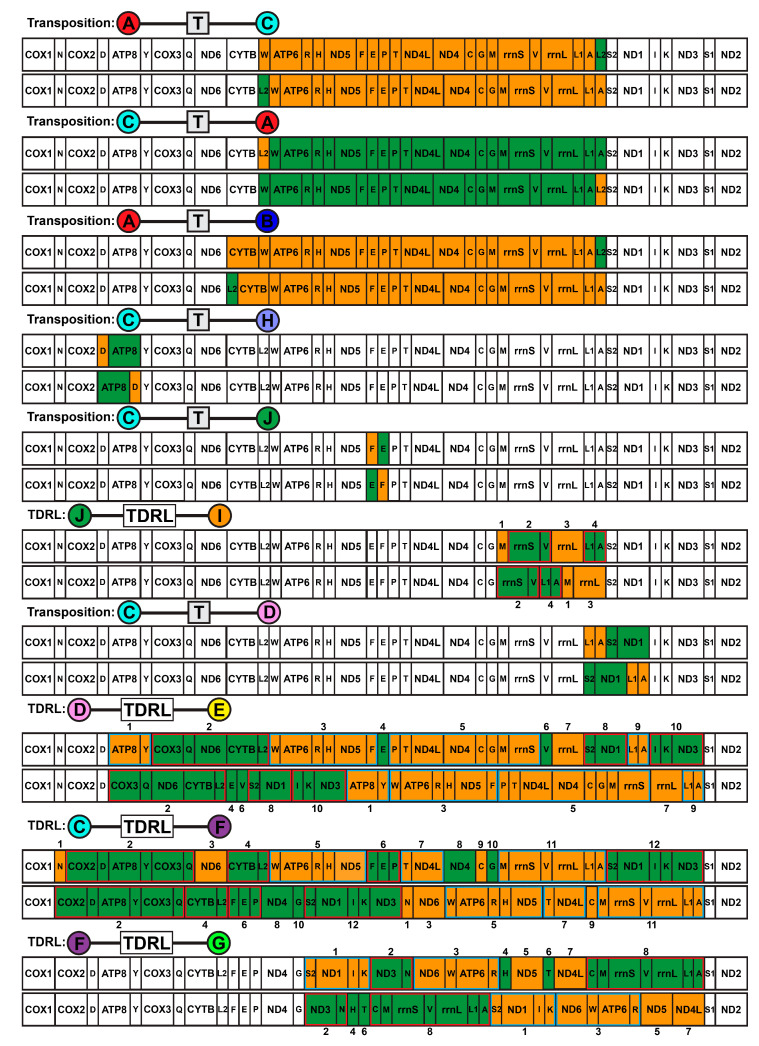
CREx results for visualization of the most parsimonious gene order rearrangement scenarios in Aphroditiformia, based on the 13 PCGs, two rRNAs, and 22 tRNAs. Color and letter inside the circles correspond to the gene orders identified in Figure 3. Key: T = transposition, TDRL = tandem duplication-random loss. Genes moved to the right by a transposition are highlighted in orange, and genes moved to the left by a transposition are highlighted in green. Single genes moved to the right by a TDRL are highlighted in orange. Blocks containing multiple genes moved to the right by a TDRL are highlighted in orange with a blue border. Single genes moved to the left by a TDRL are highlighted in green. Blocks containing multiple genes moved to the left by a TDRL are highlighted in green with a red border. Gene blocks involved in TDRLs are identified by consecutive numbers in the initial gene order on top; movement of these gene blocks is visualized by the new placement of these numbered gene blocks in the rearranged gene order underneath.

The TreeREx results of the Aphroditiformia 37-gene mitochondrial gene order dataset indicate that three transpositions occurred within shallow-water aphroditiforms, while three transpositions and four TDRLs occurred within Macellicephalinae (deep-sea and anchialine cave-dwelling polynoids) (Figure 4). Within shallow-water scaleworms, an early transposition event gave rise to gene order C from gene order A, which is the ancestral gene order among Aphroditiformia. The reverse transposition occurred later to reinstate gene order A among some members of Sigalionidae, thus rendering taxa with gene order A polyphyletic. Within Macellicephalinae, transpositions gave rise to gene orders D, H, and J, all of which derive from gene order C, inferred as ancestral for Polynoidae, Macellicephalinae, and Lepidonotopodini. Instances of TDRLs resulted in the emergence of gene orders E, F, G, and I. Of these, only gene order F derives directly from the ancestral gene order C. All other TDRLs occur on branches inferred to have already attained derived gene orders states through transpositions or through a TDRL in the case of the evolution of gene order G from gene order F. Gene movements associated with these events (reconstructed using CREx), are shown in Figure 5.

**Figure A38:**
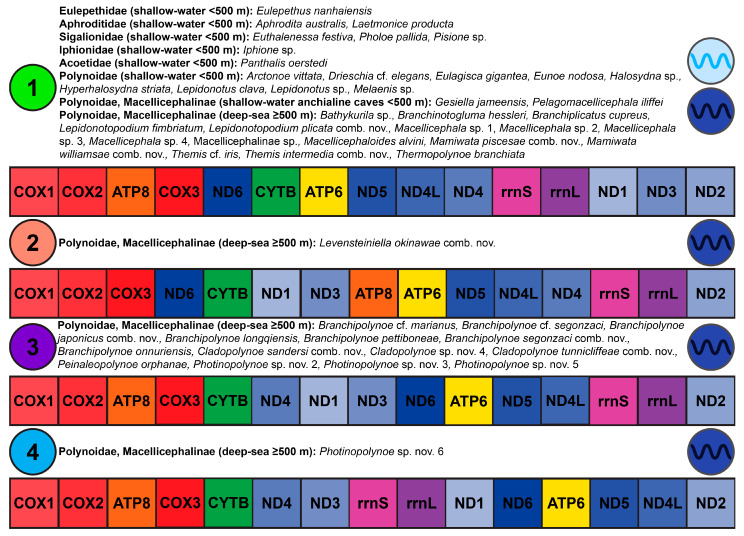
Mitochondrial gene orders, including the 13 protein-coding genes (PCGs) and two rRNAs, present in Aphroditiformia. The light blue wave symbol indicates that the gene order is present in shallow-water scaleworms, and the dark blue wave symbol indicates that the gene order is present in deep-sea scaleworms.

**Figure A39:**
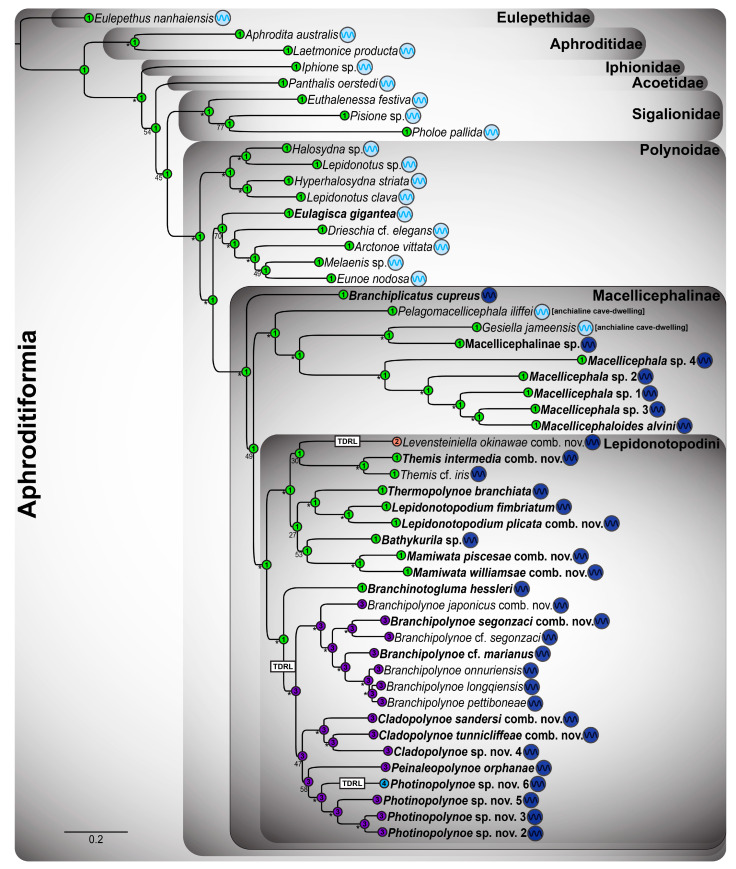
TreeREx ancestral state reconstructions of mitochondrial gene order and most parsimonious gene order rearrangement scenarios, based on the 13 PCGs and two rRNAs. Mitochondrial gene orders are indicated at ancestral nodes and at the tips of extant taxa. Colors and numbers inside the circles correspond to the gene orders identified in Figure A38. Gene order rearrangement scenarios are listed on corresponding branches. Numbers next to nodes are ML bootstrap percentages. Key: * indicates support values ≥ 95%, TDRL = tandem duplication-random loss. Light blue wave symbols at the tips of taxa identify shallow-water scaleworms, and dark blue wave symbols identify deep-sea scaleworms. Taxon names set in bold denote the scaleworm species for which mitogenomes were assembled in this study. Taxa that had missing or incomplete mitochondrial genes were excluded from this analysis.

**Figure A40:**
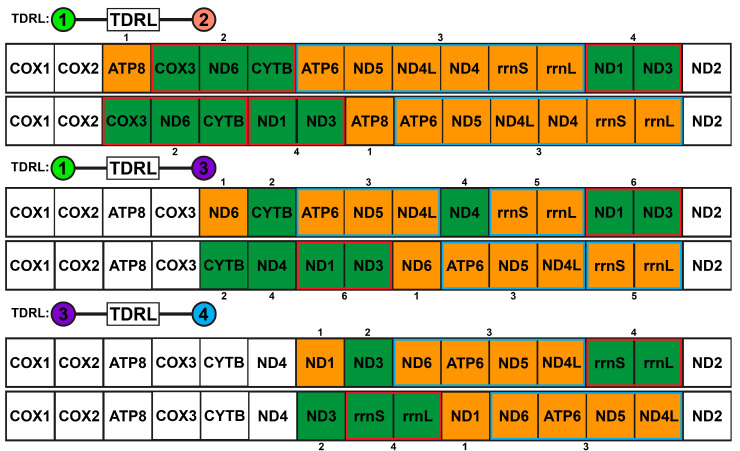
CREx results for visualization of the most parsimonious gene order rearrangement scenarios in Aphroditiformia, based on the 13 PCGs and two rRNAs. Color and number inside the circles correspond to the gene orders identified in Figure A38. Key: TDRL = tandem duplication-random loss. Single genes moved to the right by a TDRL are highlighted in orange. Blocks containing multiple genes moved to the right by a TDRL are highlighted in orange with a blue border. Single genes moved to the left by a TDRL are highlighted in green. Blocks containing multiple genes moved to the left by a TDRL are highlighted in green with a red border. Gene blocks involved in TDRLs are identified by consecutive numbers in the initial gene order on top; movement of these gene blocks is visualized by the new placement of these numbered gene blocks in the rearranged gene order underneath.

Four mitochondrial gene orders were present within Aphroditiformia when considering only the 13 PCGs and two rRNAs (Figure A38). Gene order 1 was shared by all shallow-water aphroditiforms in addition to 16 deep-sea macellicephalins. Gene orders 2–4 were exclusively present in deep-sea macellicephalins. Inference of gene rearrangement events with TreeREx indicated that three TDRLs occurred within Lepidonotopodini (Figure A39) along the same branches as in the 37-gene analysis (see Figure 4). Gene movements involved in the production of these different mitogenome configurations (inferred with CREx) are shown in Figure A40.

### 3.5. Polynoidae Selection of Mitochondrial PCGs

**Figure 6:**
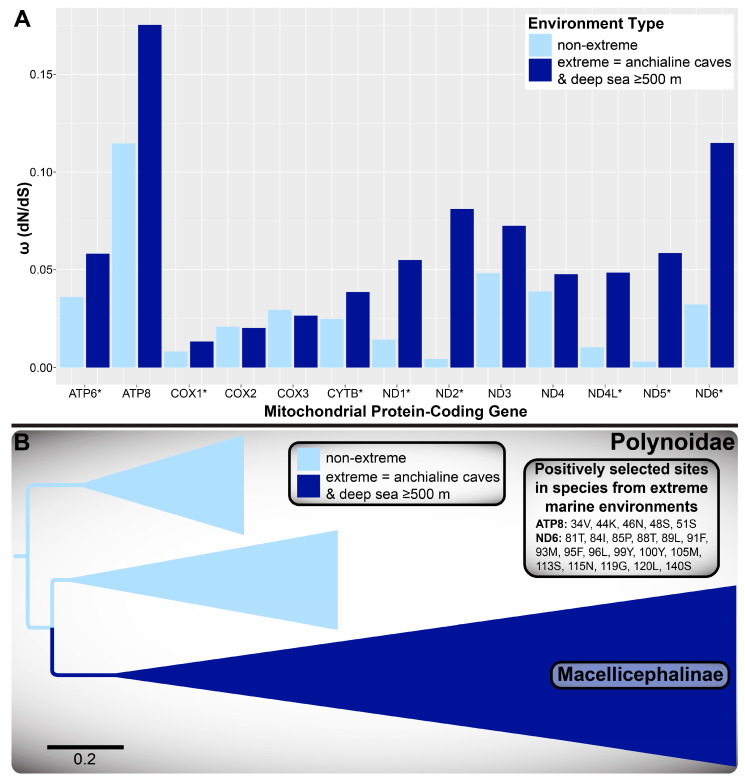
PAML codeml results for mitochondrial PCGs in Polynoidae from non-extreme vs. extreme marine environments, which were defined as shallow anchialine caves and deep-sea habitats. (**A**) Branch model results showing the comparison of ω (dN/dS) values in non-extreme vs. extreme polynoid species for each mitochondrial PCG. * indicates that the alternative hypothesis (different ω for non-extreme and extreme polynoids) is significantly better than the null hypothesis (same ω), with *p* < 0.01. (**B**) Branch-site model A results for mitochondrial PCGs in non-extreme vs. extreme Polynoidae, showing mitochondrial genes and amino acid sites which are under positive selection (ω > 1) in polynoids from extreme marine environments.

**Figure A41:**
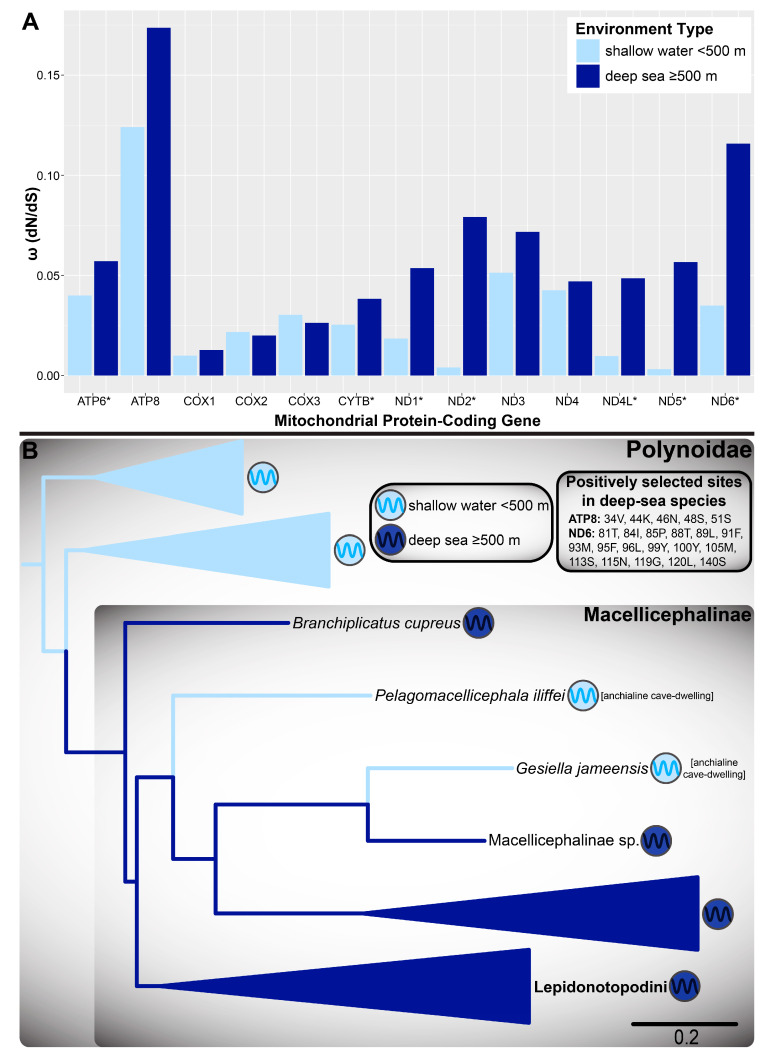
PAML codeml results for mitochondrial PCGs in shallow-water vs. deep-sea Polynoidae. (**A**) Branch model results showing the comparison of ω (dN/dS) values in shallow-water vs. deep-sea polynoid species for each mitochondrial PCG. Key: * indicates that the alternative hypothesis (different ω for shallow-water and deep-sea polynoids) is significantly better than the null hypothesis (same ω), with *p* < 0.01. (**B**) Branch-site model A results for mitochondrial PCGs in shallow-water vs. deep-sea Polynoidae, showing mitochondrial genes and amino acid sites which are under positive selection (ω > 1) in deep-sea polynoids. Amino acid sites were only considered as undergoing positive selection if the likelihood ratio tests (LRTs) comparing null vs. alternative hypotheses had *p* < 0.01, and the Bayes Empirical Bayes (BEB) values for the sites were ≥0.99. The light blue wave symbol indicates shallow-water scaleworms, and the dark blue wave symbol indicates deep-sea scaleworms.

Table S9 provides the PAML codeml results for branch model and branch-site model A tests, along with results for the LRTs with two different input trees: (1) *Deep* = deep-sea species labeled as foreground branches, and (2) *Extreme* = anchialine cave-dwelling and deep-sea species (otherwise referred to as polynoids from extreme environments) labeled as foreground branches. When using the *Deep* input tree, the branch model tests recovered significant differences in ω values between background and foreground branches for *ATP6*, *CYTB*, *ND1*, *ND2*, *ND4L*, *ND5*, and *ND6*, all of which exhibited higher ω values for deep-sea polynoids (Figure A41A, Table S9). These results indicate that relaxed purifying selection is prevalent in deep-sea polynoid mitogenomes, characterizing the evolution of seven of the 13 mitochondrial PCGs. Furthermore, the branch-site model A tests indicated that *ATP8* and *ND6* were evolving under positive selection, a signal that was detectable in 22 amino acid sites (Figure A41B, Table S9). Results obtained with the *Extreme* input tree were highly similar. Significantly greater ω values were recovered for polynoids from extreme environments for the same set of loci, in addition to *COX1* (Figure 6A, Table S9). The branch-site model A tests corroborated the same 22 amino acid sites within *ATP8* and *ND6* as evolving under positive selection (Figure 6B, Table S9).

**Figure 7:**
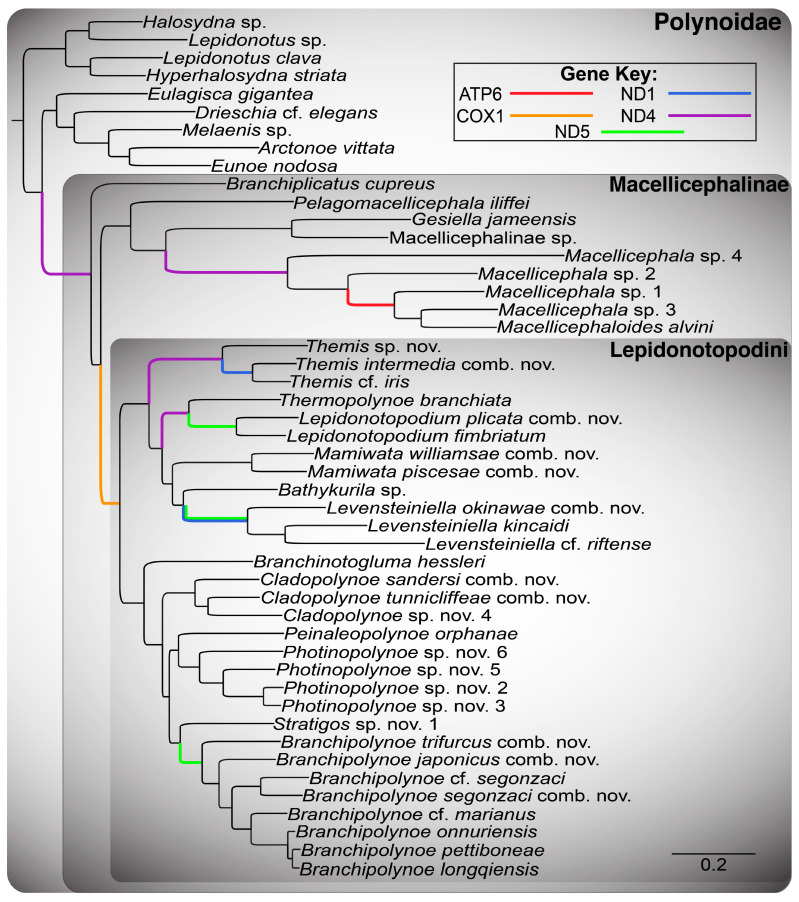
Hypothesis Testing using Phylogenies (HyPhy) adaptive Branch-Site Random Effects Likelihood (aBSREL) results for mitochondrial PCGs in non-extreme vs. extreme Polynoidae.

**Figure A42:**
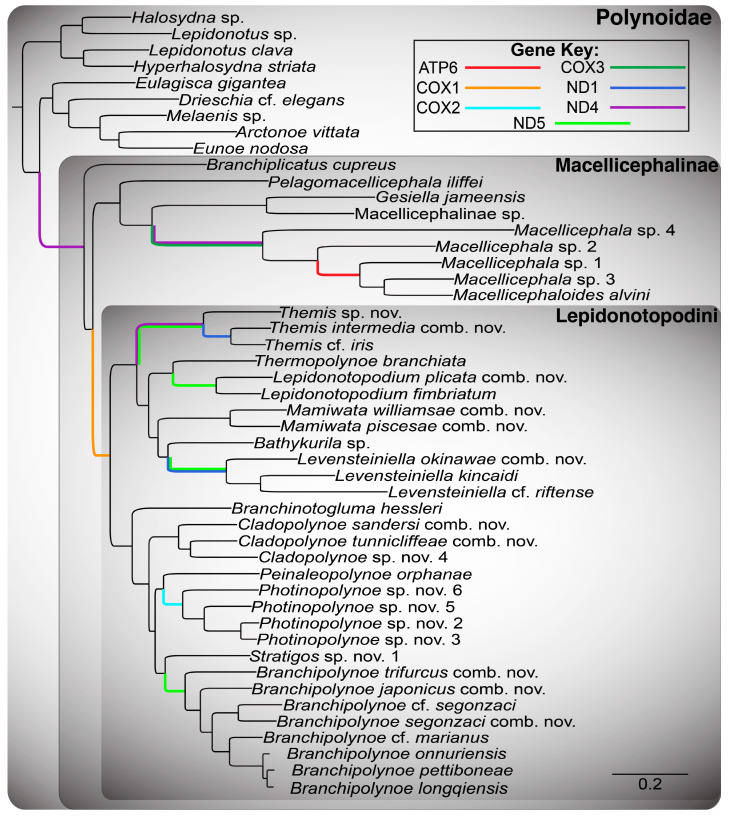
Hypothesis Testing using Phylogenies (HyPhy) adaptive Branch-Site Random Effects Likelihood (aBSREL) results for mitochondrial PCGs in shallow-water vs. deep-sea Polynoidae.

HyPhy aBSREL results using the *Deep* (Figure A42) and *Extreme* (Figure 7) input trees are highly congruent, identifying ten distinct foreground branches as having evolved under positive selection (nine in common, one unique to each analysis). Notably, these set of branches included those giving rise to lineages with derived ecological preferences: the origin of Macellicephalinae (i.e., polynoids from extreme environments) and Lepidonotopodini (i.e., polynoids known exclusively from deep-sea CBEs). Positive selection was detected in loci-encoding subunits of NADH dehydrogenase (across seven/eight distinct phylogenetic branches), cytochrome c oxidase (one/three branches), and ATP synthase (one branch).

On the other hand, BUSTED suggested no evidence of gene-wide positive selection in foreground lineages, regardless of the input tree, while MEME supported episodic positive selection of 12 amino acid sites (amongst *ATP6*, *ATP8*, *COX1*, *ND3*, *ND4L*, *ND5*, and *ND6*) when using the *Deep* input tree and seven amino acid sites (amongst *COX1*, *ND3*, *ND4*, *ND4L*, *ND5*, and *ND6*) when using the *Extreme* input tree (Table 3).

**Table 3:** HyPhy Mixed Effects Model of Evolution (MEME) results for mitochondrial PCGs, including codon sites evolving under episodic positive selection, corresponding *p*-values, and estimated number of branches under diversifying selection obtained from the four analytical replicates. If there was variation across replicates, a range is provided for the listed *p*-value or number of branches. Only results with *p* < 0.01 were considered as significant and are listed in this table. If the number of branches listed for a given codon site is 0, this indicates that the codon is under pervasive diversifying selection, likely applying to all foreground branches.

Gene	Codon Site	*p*-Values (Deep Sea ≥ 500 m)	# Branches (Deep Sea ≥ 500 m)	*p*-Values (Extreme Marine Environments)	# Branches (Extreme Marine Environments)
*ATP6*	44	0.0071–0.0072	2	-	-
*ATP8*	34	0.0075–0.0091	0	-	-
*COX1*	113	0.0002	3	0.0002	3
	481	0.0064	6	0.0012	6
*ND3*	77	0.0001	6	0.0002	6
*ND4*	164	-	-	0.0095	4
*ND4L*	101	0.0002	1	0.0007	0
*ND5*	2	0.0076–0.0077	1	-	-
	64	0.0089–0.0090	7	-	-
	181	0.0024–0.0055	3	-	-
	440	0.0004	5	0.0002	4–6
	496	0.0030–0.0031	5	-	-
*ND6*	166	0.0002–0.0003	1	0.0002–0.0003	1

## 4. Discussion

In this study, we generated a wealth of both mitochondrial and nuclear genetic data for polynoids, focusing mostly on deep-sea taxa. Also, we bioinformatically assembled 31 novel polynoid mitogenomes (Table S1) and used these data to explore their phylogenetic relationships and molecular evolution, paving the way for a stable taxonomy and a more thorough understanding of molecular evolution among poorly studied species from extreme marine environments.

Our taxonomic sampling greatly expands that of previous efforts, resulting in the most comprehensive phylogenetic study of deep-sea Polynoidae to date. Our efforts resulted in the inclusion of 46 of the 55 currently known Lepidonotopodinae species, as well as a further 14 undescribed taxa. The only Lepidonotopodini species whose placement remains uncertain due to a lack of available specimens are *Bathykurila guaymasensis*, *Bathykurila zenkevitchi* (Uschakov, 1955), *Branchinotogluma burkensis*, *Lepidonotopodium atalantae*, *Lepidonotopodium jouinae*, *Lepidonotopodium minutum*, *Lepidonotopodium riftense*, *Levensteiniella iris*, and *Levensteiniella raisae*; but even among these, we included DNA data for specimens of *Lepidonotopodium* cf. *riftense* and *Levensteiniella* cf. *iris*, which matched the morphology of the corresponding descriptions but were not collected from the type localities (hence labeled cf.). Additionally, inclusion of 18 genetic loci, as opposed to the six genes previously used [2,5,6,10], allowed for further resolution of taxonomic issues involving non-monophyly for taxa, particularly regarding genera.

The phylogenetic analyses conducted herein were unable to resolve the relationships between the deep-sea polynoid nominal subfamilies Macellicephalinae and Lepidonotopodinae. Although some analyses recovered the reciprocal monophyly of these two clades (Figure 2 and Figure A2), most results were not consistent with them. In many analyses, Lepidonotopodinae taxa were recovered nested within Macellicephalinae (Figure 1, Figure A1 and Figure A3), in agreement with previous transcriptome-based results [3]; in others, Macellicephalinae was placed inside Lepidonotopodinae (Figure A4). These results are entirely driven by *Branchiplicatus cupreus*, a ‘rogue’ taxon that is found in alternative positions with low support values. Given the otherwise congruent results of the 18NT-All-Taxa (Figure 1), 6NT (Figure A1), and 18NT (Figure A3) ML and BI phylogenies, and the general agreement of these with the phylogeny of Gonzalez et al. [3], Lepidonotopodinae is here regarded as a junior synonym of Macellicephalinae. This emended diagnosis of Macellicephalinae includes a selection of polynoids from extreme marine environments, defined as shallow anchialine caves and deep-sea ecosystems ≥ 500 m, which form a well-supported clade across all 18-gene ML and BI phylogenetic analyses (Figure 1, Figure 2, Figure A3 and Figure A4). In turn, the tribe name Lepidonotopodini is applied to a consistently well-supported macellicephalin clade including species inhabiting deep-sea CBEs, which includes both non-branchiate genera (*Bathykurila*, *Lepidonotopodium*, *Levensteiniella*, *Mamiwata* gen. nov., and *Themis* gen. nov.) and arborescent branchiate genera (*Branchinotogluma*, *Branchipolynoe*, *Cladopolynoe* gen. nov., *Peinaleopolynoe*, *Photinopolynoe* gen. nov., *Stratigos* gen. nov., and *Thermopolynoe*) (Figure 1, Figure 2, Figure A1, Figure A2, Figure A3 and Figure A4). The only branchiate genus not included in Lepidonotopodini is *Branchiplicatus*, containing solely *Branchiplicatus cupreus* (plicate branchiae), which we exclude from the tribe due to its unresolved phylogenetic position (Figure 1, Figure 2, Figure A1, Figure A2, Figure A3 and Figure A4). Further efforts should focus on finding a robust phylogenetic placement (and taxonomy) for *B. cupreus*, which is left as Macellicephalinae *incertae sedis*.

As in previous studies [2,5,6,10], *Lepidonotopodium* was recovered as non-monophyletic. In this study, all analyses recovered *Lepidonotopodium* species in three distinct well-supported clades within Lepidonotopodini (Figure 1, Figure 2, Figure A1, Figure A2, Figure A3 and Figure A4):

(1) Clade “*Lepidonotopodium*” containing *Lepidonotopodium fimbriatum* (type species) and *Lepidonotopodium plicata* comb. nov.;
(2) Clade “*Mamiwata* gen. nov.” containing *Mamiwata piscesae* comb. nov. (type species), *Mamiwata* cf. *williamsae*, and *Mamiwata williamsae* comb. nov.;
(3) Clade “*Levensteiniella*” containing *Levensteiniella kincaidi* (type species), *Levensteiniella* cf. *riftense*, and *Levensteiniella okinawae* comb. nov.

Furthermore, *Levensteiniella* was newly recovered as non-monophyletic, with corresponding species present in three distinct, well-supported clades within Lepidonotopodini (Figure 1, Figure 2, Figure A1, Figure A2, Figure A3 and Figure A4):

(1) Clade “*Lepidonotopodium*” (see above);
(2) Clade “*Levensteiniella*” (see above);
(3) Clade “*Themis* gen. nov.” containing *Themis intermedia* comb. nov. (type species), *Themis* cf. *iris, Themis longqiensis* comb. nov.*, Themis manusensis* comb. nov.*, Themis pettiboneae* comb. nov.*, Themis undomarginata* comb. nov.*,* and *Themis* sp. nov.

Although *Branchinotogluma* was recovered as non-monophyletic in previous Lepidonotopodinae phylogenetic studies [2,4,5,6,9,10], taxonomic revisions had not addressed the issue, further complicated by the description of new *Branchinotogluma* species. In this study, all phylogenetic trees produced recovered *Branchinotogluma* species in five different well-supported phylogenetic positions within Lepidonotopodini (Figure 1, Figure 2, Figure A1, Figure A2, Figure A3 and Figure A4):

(1) “*Branchinotogluma*” containing only *Branchinotogluma hessleri* (type species);
(2) Clade “*Cladopolynoe* gen. nov.” containing *Cladopolynoe tunnicliffeae* comb. nov. (type species), *Cladopolynoe* cf. *sandersi, Cladopolynoe jiaolongae* comb. nov.*, Cladopolynoe sandersi* comb. nov.*,* and *Cladopolynoe* sp. nov. 4;
(3) Clade “*Photinopolynoe* gen. nov.” containing *Photinopolynoe ovata* comb. nov. (type species), *Photinopolynoe elytropapillata* comb. nov., *Photinopolynoe kaireiensis* comb. nov., *Photinopolynoe pettiboneae* comb. nov., *Photinopolynoe robusta* comb. nov., *Photinopolynoe sagamiensis* comb. nov., *Photinopolynoe* sp. nov. 2–3, and *Photinopolynoe* sp. nov. 5–6;
(4) Clade “*Stratigos* gen. nov.” containing *Stratigos bipapillata* comb. nov. (type species) and *Stratigos* sp. nov. 1;
(5) Clade “*Branchipolynoe*” containing *Branchipolynoe* cf. *marianus*, *Branchipolynoe* cf. *segonzaci*, *Branchipolynoe japonicus* comb. nov., *Branchipolynoe marianus* comb. nov., *Branchipolynoe nanhaiensis* comb. nov., *Branchipolynoe nikkoensis* comb. nov., *Branchipolynoe segonzaci* comb. nov., and *Branchipolynoe trifurcus* comb. nov., in addition to 10 symbiotic *Branchipolynoe* species.

Resolution of non-monophyly in *Lepidonotopodium* and *Levensteiniella* required the amendment of both genera and the erection of *Mamiwata* gen. nov. and *Themis* gen. nov. to include species corresponding to these clades (Figure 1, Figure 2, Figure A1, Figure A2, Figure A3 and Figure A4). Furthermore, non-monophyly in *Branchinotogluma* was resolved by reducing it to a monotypic genus containing only the type species *B. hessleri*, erecting *Cladopolynoe* gen. nov., *Photinopolynoe* gen. nov., and *Stratigos* gen. nov. for the taxa in these corresponding clades, and expanding *Branchipolynoe* to include the free-living species that form a grade with respect to the ten symbiotic species (Figure 1, Figure 2, Figure A1, Figure A2, Figure A3 and Figure A4).

In this study, taxonomic revisions were not made for the following Lepidonotopodini genera: *Bathykurila*, *Peinaleopolynoe*, and *Thermopolynoe*. The need for taxonomic revisions of *Bathykurila* was not investigated here, owing to the lack of DNA sequence data for the two currently known *Bathykurila* species, *B. zenkevitchi* (type species) and *B. guaymasensis*. In future studies, phylogenetic placement of *B. guaymasensis* and *B. zenkevitchi* specimens collected from the corresponding type localities (Guaymas Basin and Kuril–Kamchatka Trench, respectively) will be critical for delineating *Bathykurila* and assessing whether the genus is monophyletic. *Peinaleopolynoe* was recovered as a monophyletic group in all phylogenetic analyses, containing *P. sillardi* (type species), *P. elvisi, P. goffrediae, P. mineoi, P. orphanae,* and *P. santacatalina* (Figure 1, Figure 2, Figure A1, Figure A2, Figure A3 and Figure A4); thus, taxonomic revisions for *Peinaleopolynoe* were not necessary. Finally, *Thermopolynoe branchiata* was recovered as sister species to clade “*Lepidonotopodium*” across all analyses conducted and with consistently high support values (Figure 1, Figure 2, Figure A1, Figure A2, Figure A3 and Figure A4). Given that *Thermopolynoe* is monotypic, containing only *T. branchiata*, the current phylogenetic placement does not require taxonomic revision.

Analysis of the 31 polynoid mitogenomes newly assembled herein revealed insights into their mitogenome organization, nucleotide statistics, and codon usage. All 31 polynoid mitogenomes exhibited a lower GC than AT content (Table S3), consistent with aphroditiform mitogenomes sourced from other studies [4,102,111,112,113,114,115]. Furthermore, novel mitogenomes exhibited strongly negative GC skews, as well as moderately negative AT skews (Tables S3 and S4), with skew ranges similar to those previously reported for Aphroditiformia (e.g., −0.364 to −0.190 GC skews and −0.220 to −0.025 AT skews across the 16 mitogenomes assembled in Zhang et al. [4]). This indicates that a higher frequency of C compared to G is a common pattern in scaleworm mitogenomes [4,102,111,112,113,115]; although some exceptions have been reported (e.g., *Eunoe nodosa* has a positive GC skew [114]).

RSCU analysis of novel polynoid mitogenomes (Table S5) highlighted synonymous codon usage biases in mitochondrial PCGs, which are likely to arise from “silent” mutations that do not alter the specified amino acid, coupled with overall biased mutational patterns [116,117,118]. Overall, 18 codons were found to be under-represented in the mitogenomes of all/most aphroditiforms, with 13 others showing evidence of over-representation. In this study, we did not compare codon usage and amino acid composition between shallow-water and deep-sea scaleworms, but an in-depth comparative transcriptomics study by Zhang et al. [119] revealed statistical differences between groups. Furthermore, a previous study by Yang et al. [120] showed that a change in amino acid composition to have a more abundant concentration of basic polar amino acids can be considered an adaptation to environments with low temperatures, characteristic of both anchialine cave systems and deep-sea habitats.

Additionally, the start and stop codons of mitochondrial PCGs also showed variability herein. For instance, ATG was the predominant start codon, but ATT and ATA were also present amongst the 13 PCGs. Also, the alternative start codons ATC, GTG, TTG, and CTG were observed in some cases, indicating flexibility in the initiation of mitochondrial protein synthesis. Alternative start codons have been previously documented in other annelid mitogenomes, including interstitial *Ophryotrocha* Claparède & Mecznikow, 1869 species within the family Dorvilleidae Chamberlin, 1919 [121]; shell-boring *Polydora* Bosc, 1802 of the family Spionidae Grube, 1850 [122]; leeches of the family Glossiphoniidae Vaillant, 1890 [123]; and *Micronephthys minuta* (Théel, 1879) within the family Nephtyidae Grube, 1850 [124]; as well as other invertebrates, including weevils [125], amphipods [126], and lice [127]. Regarding stop codons, either TAA (several which were incomplete and reflected as T in the gene sequence) or TAG were used. The widespread occurrence of TAA stop codons that are completed post-transcriptionally by the addition of 3′ A residues to the mRNA reflects diversity in the termination of protein synthesis in these mitogenomes [110]. This TAA phenomenon is also common in other scaleworm mitogenomes [4,102,111,112,113,114,115], various annelid mitogenomes (e.g., Chrysopetalidae Ehlers, 1864 [128] and Spionidae [122]), and various invertebrate mitogenomes (e.g., Coenobitidae Dana, 1851 and Albuneidae Stimpson, 1858 [129]).

Mitochondrial gene order analyses revealed increased rearrangement events within polynoids from extreme marine environments (Macellicephalinae) compared to shallow-water aphroditiforms. When considering the 37-gene dataset, ten mitochondrial gene orders were present within Aphroditiformia, seven of which were exclusive to macellicephalins (Figure 3). Gene orders G and I–J, exclusively present in deep-sea polynoids, were newly documented herein (Figure 3), while gene orders A–F and H had been previously recovered [4,102,111,112,113,114,115]. When considering the 15-gene dataset, four gene orders were present, three of which were exclusive to Lepidonotopodini taxa (Figure A38), and one of which (gene order 4, found in *Photinopolynoe* sp. nov. 6) was newly discovered here (Figure A38). Gene order 1 (Figure A38), which matches the proposed putative ground pattern of mitochondrial PCGs and rRNAs in Pleistoannelida [130,131], was shared by shallow and deep taxa, suggesting a conserved mitochondrial gene order that has persisted despite environmental differences. The TreeREx analysis of the 37-gene dataset identified three transpositions that occurred within shallow-water aphroditiforms and three transpositions plus four TDRLs that occurred within Macellicephalinae (Figure 4 and Figure 5). That of the 15-gene dataset identified three TDRLs that occurred within Lepidonotopodini (Figure A39 and Figure A40).

As in this study, other annelid mitogenome studies have revealed diverse gene orders deviating from conserved ancestral ones [4,121,122,132,133,134,135]. Interestingly, Struck et al. [131] found that base composition variables—including biases in nucleotide or amino acid frequencies in mitogenomes, especially GC skew—and increased substitution rates were better predictors of mitochondrial gene order evolution than environment type (e.g., shallow, deep, extreme) across Annelida. Future studies should reassess the relative importance of these intrinsic and extrinsic factors using a comprehensively sampled dataset of more closely related taxa, such as the Aphroditiformia dataset presented here.

The selection analyses of mitochondrial PCGs in Polynoidae provided valuable insight into the evolutionary processes shaping mitogenomic adaptation in macellicephalin polynoids native to extreme marine environments. Overall, the selection results provided support for the hypothesis that anchialine cave-dwelling and deep-sea macellicephalin polynoids share mitogenomic adaptations that allow them to cope with similar environmental stressors, as indicated by a signal of relaxed purifying selection in over half of the mitochondrial PCGs (Figure 6A, Table S9). Interestingly, relaxed purifying selection of mitochondrial PCGs has been documented in several other deep-sea invertebrates [128,136,137,138,139] in addition to deep-sea aphroditiforms [4], suggesting that this pattern of mitochondrial evolution is a common mechanism for adaptation to extreme marine environments. Furthermore, all analyses performed detected evidence of positive selection across various loci encoding for subunits of ATP synthase and NADH dehydrogenase, as well as some signal for positive selection in *COX1* (Figure 6B and Figure 7 and Table 3 and Table S9). Similar patterns of positive selection have been detected in mitochondrial PCGs across lineages of diverse deep-sea taxonomic groups, such as sea cucumbers [136,140], vesicomyid bivalves [141], sea anemones [142], bathymodiolin mussels [138], alvinocaridid shrimps [139,143], a deep-sea crab [144], endothermic opah species [145], asteroids [146], chrysopetalid annelids [128], and corals [137]. Overall, these results provide evidence that episodic positive selection of amino acid sites in mitochondrial PCGs, which encode for inner mitochondrial membrane (IMM) proteins that constitute the invaluable oxidative phosphorylation (OXPHOS) complexes [147,148,149], are essential for adaptation to life in the deep sea across a wide array of metazoans. Our results indicate that this unique history of mitochondrial molecular evolution is also shared by anchialine cave-dwelling lineages. Therefore, it can be posited that these positively selected sites are important for preserving energy production and metabolic function across a range of extreme marine habitats.

Overall, this study reveals a dual pattern of relaxed purifying selection and episodic positive selection in mitogenomes of macellicephalin polynoids, which reflects a complex interplay between conservation of essential functions and the need for innovation in response to environmental extremes. Future studies should further explore polynoid selection in nuclear PCGs through comparative omics methods using assembled and annotated genomes and transcriptomes.

## 5. Conclusions

This study not only addresses taxonomic revisions of several non-monophyletic groups within deep-sea polynoids, but also offers insight into the mitochondrial evolutionary mechanisms that have allowed for polynoids to thrive in extreme marine environments. Our efforts, supported through the assembly of 31 novel mitogenomes, reveal that polynoids from extreme environments have experienced increased rates of gene order rearrangements, relaxed purifying selection in eight PCGs, and positive selection along a proportion of lineages across several PCGs (notably in loci encoding subunits of NADH dehydrogenase). These results are indicative of a dynamic history of molecular evolution associated with transitions to extreme marine environments, as well as the presence of shared metabolic adaptations suited for both anchialine caves and deep-sea habitats. We also provided further evidence that the positive selection of mitochondrial PCGs is a landmark of adaptation to life in the deep sea.

Finally, our comprehensive phylogenetic study of deep-sea Polynoidae allowed for a long overdue taxonomic revision of the clade. Consequently, Macellicephalinae was emended to include all genera previously classified in Lepidonotopodinae, along with the five genera newly erected: *Cladopolynoe* gen. nov., *Mamiwata* gen. nov., *Photinopolynoe* gen. nov., *Stratigos* gen. nov., and *Themis* gen. nov. Furthermore, we applied the tribe name Lepidonotopodini to a consistently well-supported macellicephalin clade inhabiting deep-sea CBEs, which includes both non-branchiate genera and arborescent branchiate genera. These efforts pave the way for a robust and stable taxonomy and an evolutionary framework for Lepidonotopodini polynoids.

## Figures and Tables

**Table 1:** Type locality and collection details for polynoid specimens sequenced in this study.

Species	Specimen Voucher	Type Locality	Collection Locality	Collection Depth (m)	Collection Date	Ship and Gear
*Bathykurila* sp.	SIO-BIC A10920	N/A	California, Rosebud Whalefall off San Diego	845	20 June 2014	R/V *Western Flyer* and ROV *Doc Ricketts*
*Branchinotogluma* cf. *marianus* (Pettibone, 1989) [26]	SIO-BIC A13116	N/A	W. Pacific Ocean, North Fiji Basin, White Lady	1990–1991	30 May 2005	R/V *Melville* and ROV *Jason II*
*Branchinotogluma hessleri* Pettibone, 1985 [27]	SIO-BIC A12940	Pacific Ocean, off western Mexico, 21° N	Mexico, Gulf of California, Alarcon Rise	2299	20 April 2015	R/V *Western Flyer* and ROV *Doc Ricketts*
*Branchinotogluma marianus* (Pettibone, 1989) [26]	SIO-BIC A16396	W. Pacific Ocean, Mariana Back-Arc Basin	W. Pacific Ocean, Mariana Back-Arc Basin	3278	11 December 2016	R/V *Falkor* and ROV *SuBastian*
*Branchinotogluma sandersi* Pettibone, 1985 [27]	MCZ-IZ 70173.2	Galapagos Rift, Rose Garden vents	Galapagos Rift, Tempus Fugit Vent Field	2568	19 June 2015	E/V *Nautilus* and ROV *Hercules*
*Branchinotogluma segonzaci* (Miura & Desbruyères, 1995) [28]	SIO-BIC A13118	W. Pacific Ocean, Lau Back-Arc Basin, Vai Lili	W. Pacific Ocean, Lau Back-Arc Basin, Tui Malila	1891	20 May 2005	R/V *Melville* and ROV *Jason II*
*Branchinotogluma segonzaci*	SIO-BIC A4659	W. Pacific Ocean, Lau Back-Arc Basin, Vai Lili	W. Pacific Ocean, Lau Back-Arc Basin, Tui Malila	1891	20 May 2005	R/V *Melville* and ROV *Jason II*
*Branchinotogluma* sp. nov. 1	MCZ-IZ 68096	N/A	Galapagos Rift, Tempus Fugit Vent Field	2599	20 June 2015	E/V *Nautilus* and ROV *Hercules*
*Branchinotogluma* sp. nov. 2	MCZ-IZ 68063	N/A	Galapagos Rift, Tempus Fugit Vent Field	2563	19 June 2015	E/V *Nautilus* and ROV *Hercules*
*Branchinotogluma* sp. nov. 3	SIO-BIC A13128	N/A	Costa Rica, Jaco Scar	1834	7 January 2019	R/V *Falkor* and ROV *SuBastian*
*Branchinotogluma* sp. nov. 4	SIO-BIC A9998	N/A	Mexico, Gulf of California, Pescadero Basin	3666	18 November 2018	R/V *Falkor* and ROV *SuBastian*
*Branchinotogluma* sp. nov. 5	SIO-BIC A13164	N/A	California, Malibu, Point Dume	723	9 October 2018	R/V *Falkor* and ROV *SuBastian*
*Branchinotogluma* sp. nov. 6	SIO-BIC A13248	N/A	Costa Rica, Parrita Scar	1364	11 January 2019	R/V *Falkor* and ROV *SuBastian*
*Branchinotogluma trifurcus* (Miura & Desbruyères, 1995) [28]	SIO-BIC A13183	W. Pacific Ocean, North Fiji Basin, White Lady	W. Pacific Ocean, Lau Back-Arc Basin, Kilo Moana	2623–2657	18 May 2005	R/V *Melville* and ROV *Jason II*
*Branchinotogluma tunnicliffeae* (Pettibone, 1988) [29]	SIO-BIC A7717	Juan de Fuca Ridge, Axial Seamount	Juan de Fuca Ridge, South Cleft	2250	31 July 2016	R/V *Western Flyer* and ROV *Doc Ricketts*
*Branchiplicatus cupreus* Pettibone, 1985 [30]	SIO-BIC A6160	Pacific Ocean off western Mexico, 21° N	Mexico, Gulf of California, Pescadero Basin	3676–3756	18 April 2015	R/V *Western Flyer* and ROV *Doc Ricketts*
*Eulagisca gigantea* Monro, 1939 [31]	SIO-BIC A6302	Antarctic Ocean	S. Atlantic Ocean, N. South Sandwich Islands	153–420	5 October 2011	R/V *Nathaniel Palmer* and Blake Trawl
*Lepidonotopodium* cf. *riftense* Pettibone, 1984 [32]	SIO-BIC A13202	N/A	S. East Pacific Rise, 31° S	2237	29 March 2005	R/V *Atlantis* and HOV *Alvin*
*Lepidonotopodium fimbriatum* Pettibone, 1983 [33]	SIO-BIC A6153	Pacific Ocean off western Mexico, 21° N	Mexico, Gulf of California, Alarcon Rise	2300	21 April 2015	R/V *Western Flyer* and ROV *Doc Ricketts*
*Lepidonotopodium piscesae* Pettibone, 1988 [29]	SIO-BIC A7978	Juan de Fuca Ridge, Axial Seamount	Juan de Fuca Ridge, South Cleft	2250	31 July 2016	R/V *Western Flyer* and ROV *Doc Ricketts*
*Lepidonotopodium williamsae* Pettibone, 1984 [32]	MCZ-IZ 70175.3	Galapagos Rift, Rose Garden vents	Galapagos Rift, Tempus Fugit Vent Field	2562	19 June 2015	E/V *Nautilus* and ROV *Hercules*
*Levensteiniella intermedia* Pettibone, 1990 [34]	SIO-BIC A13187	E. Pacific Ocean, off northern California, Gorda Ridge	Juan de Fuca Ridge, Axial Seamount, Middle North Rift	1850	10 August 2016	R/V *Western Flyer* and ROV *Doc Ricketts*
*Levensteiniella kincaidi* Pettibone, 1985 [35]	SIO-BIC A6310	Pacific Ocean, off western Mexico, 21°N	Mexico, Gulf of California, Pescadero Basin	3676	18 April 2015	R/V *Western Flyer* and ROV *Doc Ricketts*
*Levensteiniella plicata Hourdez & Desbruyères*, 2000 [36]	SIO-BIC A14234	N. East Pacific Rise, Biovent	S. East Pacific Rise, 38°S German Flats	2216	22 March 2005	R/V *Atlantis* and HOV *Alvin*
*Macellicephala* sp. 1	SIO-BIC A11031	N/A	Monterey Submarine Canyon, Ruby Whalefall	2898	19 November 2009	R/V *Western Flyer* and ROV *Doc Ricketts*
*Macellicephala* sp. 2	SIO-BIC A3432	N/A	Mexico, Gulf of California, Alarcon Rise	2449	27 April 2012	R/V *Western Flyer* and ROV *Doc Ricketts*
*Macellicephala* sp. 3	SIO-BIC A10055	N/A	Costa Rica, Jaco Summit	730–820	6 January 2019	R/V *Falkor* and ROV *SuBastian*
*Macellicephala* sp. 4	SIO-BIC A10099	N/A	Costa Rica, Subduction Plume	3601	14 January 2019	R/V *Falkor* and ROV *SuBastian*
Macellicephalinae sp.	SIO-BIC A10163	N/A	Costa Rica, Las Gemelas Seamount	571	20 January 2019	R/V *Falkor* and ROV *SuBastian*
*Macellicephaloides alvini* Pettibone, 1989 [37]	SIO-BIC A14221	Mexico, Gulf of California, Guaymas Basin	Mexico, Gulf of California, Pescadero Basin	3677	4 November 2021	R/V *Falkor* and ROV *SuBastian*
*Peinaleopolynoe orphanae Hatch & Rouse*, in Hatch et al. 2020 [2]	SIO-BIC A6163	Mexico, Gulf of California, Pescadero Basin	Mexico, Gulf of California, Pescadero Basin	3676–3756	18 April 2015	R/V *Western Flyer* and ROV *Doc Ricketts*
*Themis* gen. nov. sp. nov.	SIO-BIC A14236	N/A	S. Pacific Ocean, Tonga-Tofua Arc, Niuatahi	1568	20 June 2018	R/V *Sonne* and ROV *Quest*
*Thermopolynoe branchiata* Miura, 1994 [38]	SIO-BIC A10925	W. Pacific Ocean, North Fiji Basin, White Lady	W. Pacific Ocean, Lau Back-Arc Basin, Kilo Moana	2623–2657	18 May 2005	R/V *Melville* and ROV *Jason II*

## Data Availability

Publicly available datasets on NCBI GenBank were analyzed in this study. See Table S1 for accession numbers. Additionally, raw reads used to assemble the mitogenomes were deposited in the NCBI GenBank SRA database under the BioProject ID PRJNA994258, which includes BioSample accession numbers SAMN36418903-SAMN36418933.

## Supplementary Materials

The following supporting information can be downloaded at: https://www.mdpi.com/article/10.3390/biology13120979/s1, **Table S1:** Collection data, vouchers, and GenBank accession numbers (*cytochrome c oxidase subunit I* (*COX1*), *16S ribosomal RNA* (*16S rRNA*), *18S ribosomal RNA* (*18S rRNA*), *28S ribosomal RNA* (*28S rRNA*), *histone h3* (*H3*), *cytochrome b* (*CYTB*), mitochondrial genome (mitogenome)) for all DNA sequences used in this study. Specimen vouchers and sequences newly reported in this study are set in bold. GenBank sequences with an asterisk next to them were assembled from Sanger sequencing data. All remaining GenBank sequences were bioinformatically assembled and extracted from genome skimming data; **Table S2:** Genome skimming details and data statistics for all 31 specimens sequenced via Next Generation Sequencing (NGS) in this study, including total number of paired-end raw reads, total length of paired-end raw reads, individual length of raw reads, number of paired-end reads post-Trimmomatic, and if applicable, number of paired-end reads post-downsampling; **Table S3:** MitoFinder parameters (metagenomic assembler and transfer ribosomal RNA (tRNA) annotator) and resulting mitogenome statistics (circularization, mitogenome length in base pairs (bp), mitogenome coverage, GC content (%), AT content (%), and incomplete or missing genes); in addition to GC and AT skew calculations for all 31 specimens sequenced via NGS in this study. Incomplete mitochondrial genes are denoted by an asterisk; **Table S4:** GC content (%), AT content (%), and GC and AT skew calculations for each of the 15 mitochondrial genes (13 protein-coding genes (PCGs) and two rRNAs) corresponding to all 31 final mitogenome assemblies; **Table S5:** Frequencies (%) of each codon present in the mitochondrial PCGs for a given amino acid and the calculated relative synonymous codon usage (RSCU) values (listed in parentheses) corresponding to the 13 PCGs of all 31 scaleworm species skimmed in this study. RSCU values greater than or equal to 1.6 are set in bold; RSCU values less than or equal to 0.6 are underlined; **Table S6:** Outgroups and best-fit models selected for all maximum likelihood (ML) and Bayesian inference (BI) phylogenies generated; **Table S7:** Descriptive information for several morphological traits within Lepidonotopodini genera revised or erected herein, which includes *Branchinotogluma*, *Branchipolynoe*, *Cladopolynoe* gen. nov., *Lepidonotopodium*, *Levensteiniella*, *Mamiwata* gen. nov., *Photinopolynoe* gen. nov., *Stratigos* gen. nov., and *Themis* gen. nov. The genus placement for species highlighted in orange (referred to as cf. in this study) is not certain due to lack of DNA data from the corresponding type locality. However, classification is suggested given that specimen morphology matches that of the original species descriptions. The genus placement for species highlighted in red is not certain due to lack of DNA data altogether; therefore, classification is suggested based on morphology only; **Table S8:** Morphological diagnostic characters of the 12 Lepidonotopodini genera; **Table S9:** PAML codeml results using the control variable CodonFreq = 7 (FMutSel) for the branch model and branch-site model A tests with two different input trees: (1) deep-sea species ≥ 500 m labeled as foreground branches and (2) anchialine cave-dwelling and deep-sea species ≥ 500 m (otherwise referred to as species from extreme marine environments) labeled as foreground branches; **File S1:** Example PAML codeml control file for branch model alternative hypothesis; **File S2:** Example PAML codeml control file for branch model null hypothesis; **File S3:** Example PAML codeml control file for branch-site model A alternative hypothesis; **File S4:** Example PAML codeml control file for branch-site model A null hypothesis.
